# Visible-light-driven SAQS-catalyzed aerobic oxidative dehydrogenation of alkyl 2-phenylhydrazinecarboxylates†

**DOI:** 10.1039/d2ra05842a

**Published:** 2022-10-24

**Authors:** Van Hieu Tran, Hee-Kwon Kim

**Affiliations:** Department of Nuclear Medicine, Molecular Imaging & Therapeutic Medicine Research Center, Jeonbuk National University Medical School and Hospital Jeonju 54907 Republic of Korea hkkim717@jbnu.ac.kr; Research Institute of Clinical Medicine of Jeonbuk National University-Biomedical Research Institute of Jeonbuk National University Hospital Jeonju 54907 Republic of Korea

## Abstract

Azo compounds are useful molecules with a wide range of applications in organic chemistry. Here, a novel visible-light-driven oxidative dehydrogenation of alkyl 2-phenylhydrazinecarboxylates is used for the synthesis of azo compounds. This synthetic method was conducted under an aerobic environment with mild reaction conditions. Sodium anthraquinone sulfonate (SAQS) was employed as the crucial organic photocatalyst in a visible-light-driven reaction to generate various azo compounds in high yields. In addition, aerobic transformation of hydrazobenzenes to azobenzenes using visible light was successfully carried out under SAQS-mediated reaction conditions. This procedure is a practical and promising synthetic approach to produce useful azo compounds.

## Abstract 2

Efficient and facile aerobic oxidative dehydrogenation for the synthesis of azo compounds has been achieved *via* visible-light-driven reaction in the presence of sodium anthraquinone sulfonate and K_2_CO_3_.

## Graphical abstract

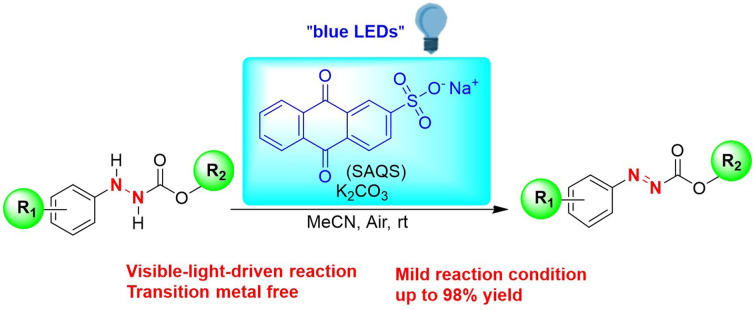

## Introduction

Azo compounds are widely used in materials science, medicinal chemistry, and organic chemistry. Azo compounds have been employed as pigments,^1^ dyes,^2^ metallochromic indicators,^3^ and medicinal agents.^4^

Alkyl 2-phenylazocarboxylates, well known azo compounds with an alkoxy carbonyl group, have received significant attention for potential uses in organic chemistry. They have been used as a catalyst for the Mitsunobu reaction^5,6^ or as building blocks for synthetic chemistry.^7^ And several phenylazocarboxylate salts have participated in cycloaddition reactions. Additionally, alkyl 2-phenylazocarboxylates were also used for Mizoroki–Heck reactions.^8,9^

**Scheme 1:**
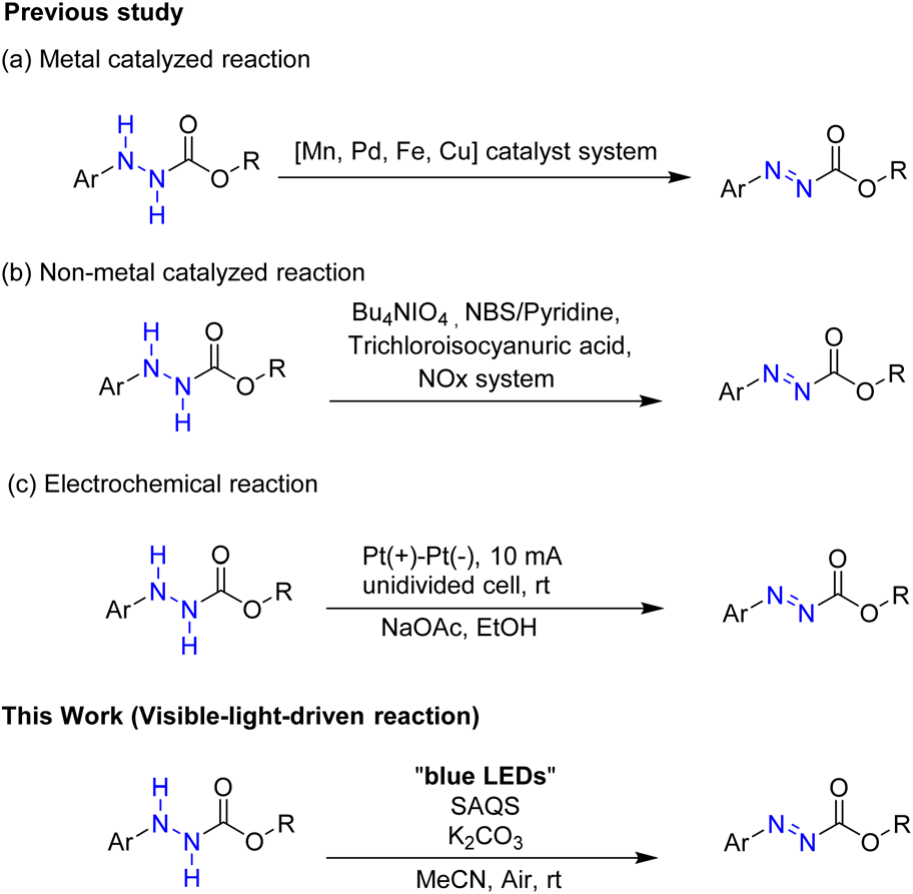
Synthetic methods for oxidation of alkyl 2-phenylhydrazinecarboxylates.

Various methods for synthesis of alkyl 2-phenylazocarboxylates have been reported (Scheme 1). They were produced from hydrazine derivatives *via* reactions utilizing various metal oxidants such as MnO_2_,^10^ Pb(OAc)_4_,^11^ Pd/C,^12^ and iron phthalocyanine^13^ or using an HZIF@TCPP-Fe/Fe system.^14^ Several reaction systems using copper have been developed to prepare alkyl 2-phenylazocarboxylate compounds: CuCl and DMAP,^15^ Cu(OAc)_2_·H_2_O and 1,10-Phen,^16^ and PdCl_2_/CuI.^17^

Besides, several non-metal catalytic systems using *n*-Bu_4_NIO_4_,^18^ NBS and pyridine,^19^ trichloroisocyanuric acid,^20^ and NO*x*^21^ to afford alkyl 2-phenylazocarboxylates have been reported. In another method, electrochemical dehydrogenation of hydrazines was developed to give azo compounds.^22^

Visible-light-driven reaction systems have received significant attention as a promising strategy in organic synthesis due to high synthetic yields and wide tolerance of functional groups.^23–29^ Moreover, many visible-light-driven reactions are achieved under mild reaction conditions to produce desired products.^30–32^ Photocatalysts play an important role in visible-light-driven reaction systems to increase reaction efficiency.^33^ Besides, several photo-dehydrogenation reactions were reported.^34–36^

Anthraquinone (AQ) derivatives, which are useful components in medicines, were employed as organic catalysts in photo-redox or photooxygenation reactions to produce reactive oxygen species (ROS).^37^ In particular, sodium anthraquinone sulfonate (SAQS) has been effectively used as a beneficial catalyst for numerous chemical transformations such as oxidation of alcohols, asymmetric aldol reactions, and bromination.^38–44^

We are interested in development of highly efficient and practical synthetic procedures to prepare azo compounds such as alkyl 2-phenylazocarboxylates and reasoned that a reaction using visible light could be promising to achieve oxidation and dehydrogenation.

In this paper, we describe a novel visible-light-driven reaction using SAQS as an organo-photocatalyst to produce azo compounds under mild reaction conditions (Scheme 1).

## Results and discussion

As shown in Table 1, ethyl 2-phenylhydrazine-1-carboxylate was used as a model substrate and the reaction was carried out in the presence of a photocatalyst (0.03 equiv.) and additive in MeCN (2 mL) in an aerobic environment under irradiation with blue LEDs.

**Table 1:** Screening of reaction conditions for synthesis of ethyl (*E*)-2-phenyldiazene-1-carboxylate^a^

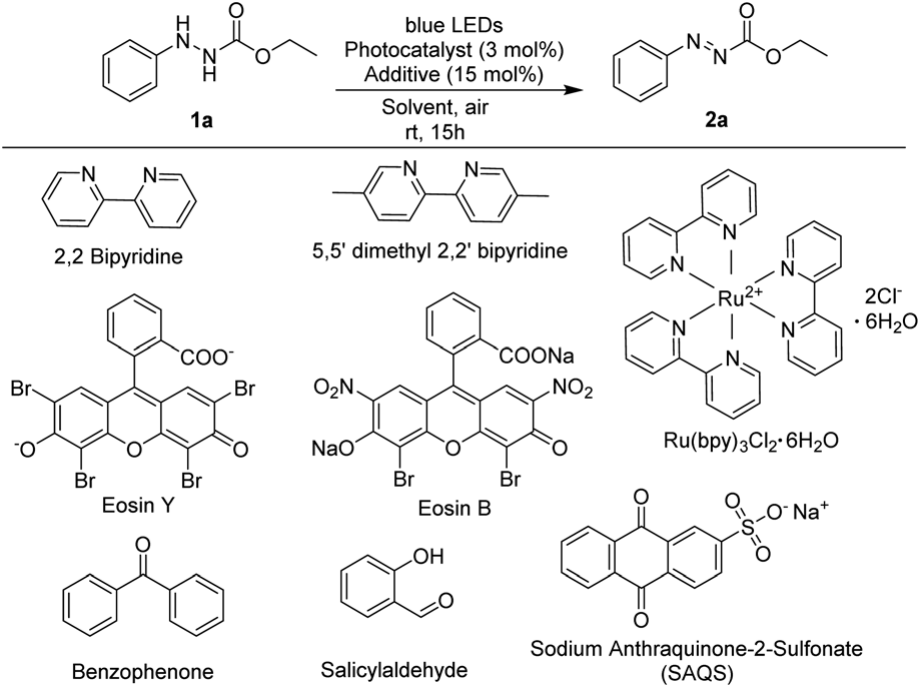				
Entry	Photocatalyst	Additive	Solvent	Yield^b^ (%)
1	—	K_2_CO_3_	MeCN	NR^c^
2	2,2-Bipyridine	K_2_CO_3_	MeCN	5
3	5.5′-Dimethyl			
	2,2′-Bipyridine	K_2_CO_3_	MeCN	7
4	Ru(bpy)_3_Cl_2_6H_2_O	K_2_CO_3_	MeCN	82
5	Eosin Y	K_2_CO_3_	MeCN	78
6	Eosin B	K_2_CO_3_	MeCN	83
7	Benzophenone	K_2_CO_3_	MeCN	71
8	Salicyladehyde	K_2_CO_3_	MeCN	NR^c^
9	SAQS	K_2_CO_3_	MeCN	97

a Reaction conditions: compound 1a (1.0 mmol), photocatalyst (0.03 mmol), additive (0.15 mmol), solvent (2 mL), room temperature, irradiation with 5 W blue LEDs for 15 h.

b Isolated yield after purification by flash column chromatography.

c No reaction.

Several commercially available photocatalysts were screened to investigate their efficiency in the oxidation reaction (Table 1, entries 2–9). When 2,2-bipyridine or 5,5-dimethyl-2,2-bipyridine was utilized as a photocatalyst, the target product was produced in less than 10% yield (5–7%) (Table 1, entries 2 and 3). Reactions using photocatalysts such as Eosin Y, Ru(bpy)_3_Cl_2_·6H_2_O, benzophenone, and Eosin B produced the desired products in yields ranging from 71% to 85% (Table 1, entries 4–7) whereas utilization of salicylaldehyde did not produce the target product. However, in reaction using sodium anthraquinone sulfonate (SAQS), ethyl (*E*)-2-phenyldiazene-1-carboxylate, the desired product, was produced in 97% yield in 15 hours (Table 1, entry 9).

To optimize the reaction conditions, a variety of bases was utilized as additives. In the presence of DIPEA, Et_3_N, DMAP, and NaHCO_3_, reactions proceeded in low yields (4–34%) (Table S1,† entries 1–5). Cs_2_CO_3_ and DBU increased the reaction performance to yields of 65% and 76%, respectively (Table S1,† entries 6 and 7). Interestingly, the target product was generated in good yield in the absence of base. K_2_CO_3_ was a useful additive as the target product was obtained in 97% yield, suggesting that the efficiency of the reaction was significantly affected by K_2_CO_3_.

Second, solvent was a crucial factor influencing the efficiency of the reaction. Performing the reaction in toluene, DCE, CH_2_Cl_2_, or 1,4-dioxane led to ethyl (*E*)-2-phenyldiazene-1-carboxylate in less than 20% yield (Table S1,† entries 9–12). The reaction in DMF and THF yielded the target product in 61% and 65%, respectively (Table S1,† entries 13–14). Remarkably, MeCN was the optimal solvent for this oxidation process, with 97% reaction yield. Thus, MeCN was selected as the solvent for further experiments.

Next, the influence of light on the reaction was studied (Table S1,† entries 15–19). When the reaction was carried out in the dark, the target product was not formed. Green LEDs, white LEDs, and compact fluorescent lights (CFLs) accelerated the visible-light-driven process and showed yields of 65%, 72%, and 70%, respectively, which were less effective oxidation reactions than using blue LEDs (97%). This finding implies that blue LEDs are more suitable for such oxidation. Additionally, reaction under ambient conditions using sunlight was also conducted, and the target product was obtained in 88% yield (Table S1,† entry 19).

A number of reactions with varied amounts of photocatalyst and additive was carried out to optimize reaction conditions (Table S2†). Better reaction efficiency was observed when using 3 mol% SAQS (Table S2,† entries 1–5). Varying the amount of base revealed that the most efficient oxidation resulted when 15% K_2_CO_3_ was employed (Table S2,† entries 3, 6–11).

The substrate scope of oxidative dehydrogenation of arylhydrazides was investigated after optimal conditions were determined (Table 2). First, ethyl 2-phenylhydrazine-1-carboxylate was converted to ethyl (*E*)-2-phenyldiazene-1-carboxylate (2a) in high yield. Several ethyl (*E*)-2-aryldiazene-1-carboxylates with electron-donating groups including methyl, ethyl, and methoxy groups at the *para* position were successfully synthesized in high yields using the visible-light-driven procedure (2b–2d). The reaction method was also effective with substrates containing electron-withdrawing groups such as chloro, bromo, and nitro groups to yield the products (2e–2g). Furthermore, visible-light-driven oxidation of substrates with a substituent at the *ortho* or *meta* position occurred readily to produce the target products (2h and 2i). Using this procedure, di-substituted substrates with dimethyl or dichloro groups were successfully converted to the corresponding products in high yields (95% and 93%, respectively) (2j and 2k), and reactions of substrates containing hetero rings and naphthalene successfully generated the desired products (2l and 2m).

**Table 2:** Scope of oxidation of alkyl 2-phenylhydrazinecarboxylates^a^

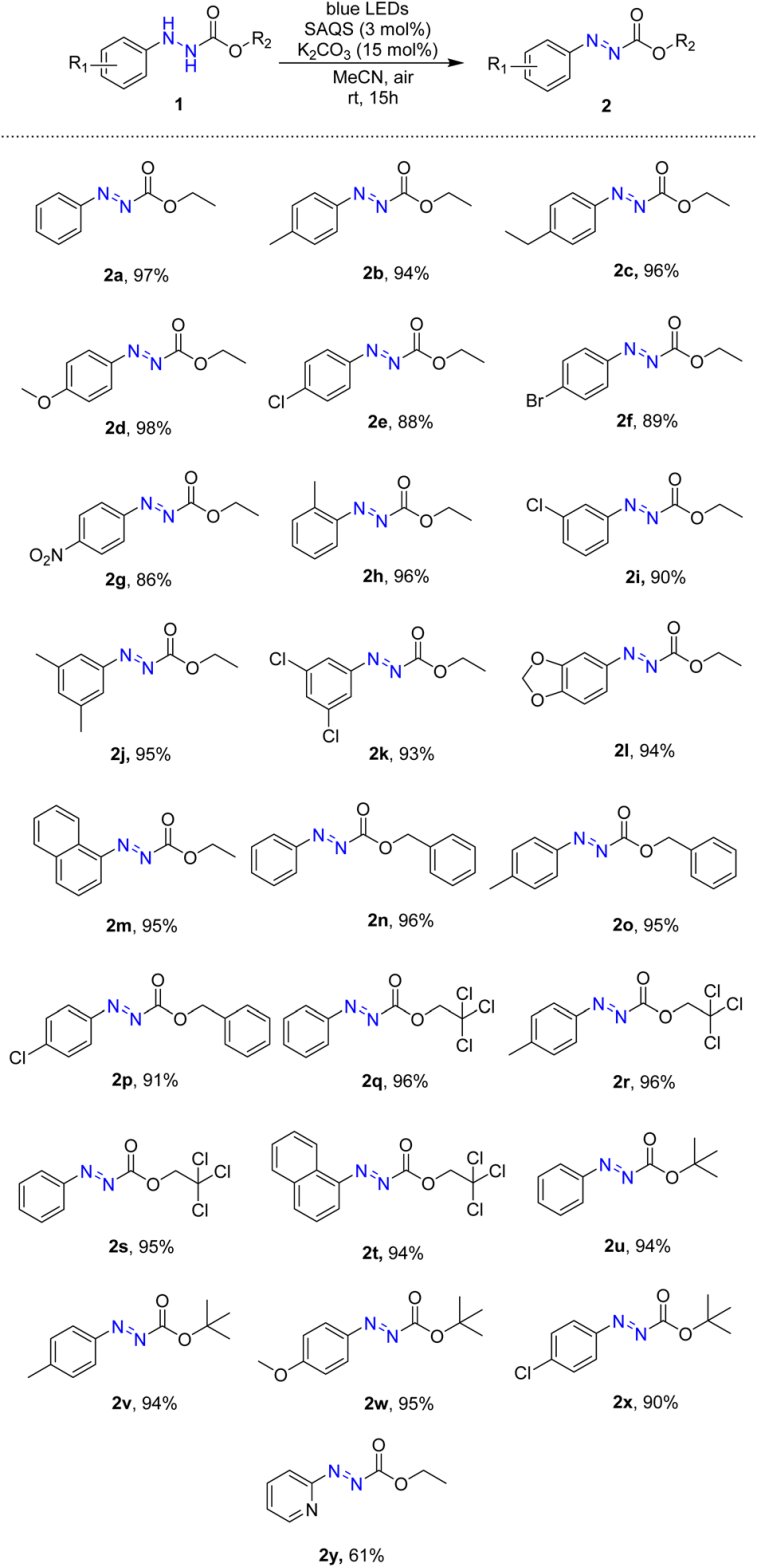

a Reaction conditions: compound 1 (1.0 mmol), SAQS (0.03 mmol), K_2_CO_3_ (0.15 mmol), MeCN (2 mL), room temperature, irradiation with 5 W blue LEDs for 15 h.

Various alkyl 2-phenylhydrazine-1-carboxylates containing benzyl, trichloroethyl, and *tert*-butyl groups were tested for this method. Under visible light, benzyl carboxylates of arylhydrazine were converted to the corresponding azo compounds in high yields (91–96%) (2n–2q). Oxidation with trichloroethyl carboxylate substrates resulted in generation of the corresponding products in 94–96% yields (2r–2t). Additionally, several *tert*-butyl azocarboxylates were synthesized in high yields *via* the visible-light-driven reaction (2u–2x). Heteroarene hydrazine carboxylates was also employed for the reaction, and the desired product was obtained in 61% yield.

In an extended application, this visible-light-driven reaction was employed in the preparation of azobenzene compounds. Reactions of hydrazobenzenes for preparation of azobenzene compounds were conducted based on the optimal reaction conditions using SAQS and K_2_CO_3_ under blue LED light. A series of substituted symmetrical and unsymmetrical hydrazobenzenes was tested for photocatalytic oxidative dehydrogenation, and these reactions provided efficient photocatalytic oxidation (Table 3).

**Table 3:** Scopes of oxidative dehydrogenation of hydrazobenzenes^a^

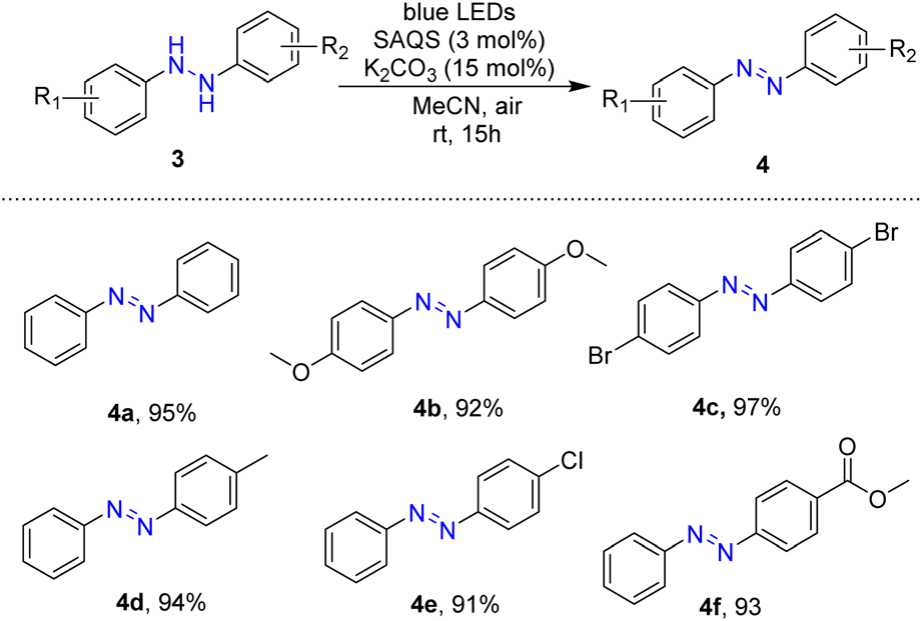

a Reaction conditions: compound 3 (1.0 mmol), SAQS (0.03 mmol), K_2_CO_3_ (0.15 mmol), MeCN (2 mL), room temperature, irradiation with 5 W blue LEDs for 15 h.

Symmetrical hydrazobenzenes containing various different electron-donating and electron-withdrawing substituents (methoxy and bromo groups) at the *para*-position of the phenyl rings were well tolerated in reactions, and the target products were obtained in high yields (4b and 4c). The reaction was also effective with unsymmetrical hydrazobenzenes with an electron-donating substituent such as a methyl group, affording the desired products in high yields (94%) (4d). Reactions of a substrate with halogen on the phenyl ring occurred smoothly, giving the desired product in 91% yield (4e). Synthesis of methyl (*E*)-4-(phenyldiazenyl)benzoate was achieved in 93% yield *via* the visible-light-driven reaction (4f).

**Scheme 2:**
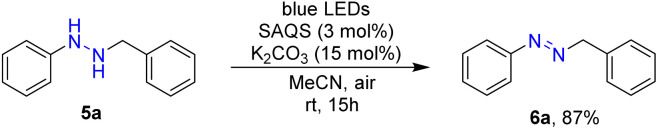
Further extended oxidative dehydrogenation (1a).

In additions, reaction of benzyl protected phenylhydrazine, a alkyl-protected phenylhydrazine, was performed (Scheme 2), and the target product was successfully prepared in 87% yields (6a).

**Scheme 3:**
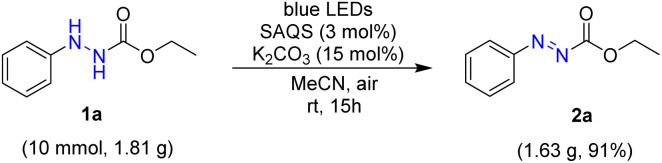
Gram-scale oxidative dehydrogenation (1a).

Next, a large-scale oxidative dehydrogenation of alkyl 2-phenylazocarboxylates was performed to demonstrate the utility of the procedure (Scheme 3). In reaction of ethyl 2-phenylhydrazine-1-carboxylate 1a (10.0 mmol, 1.81 g), the target product was obtained in 82% yield *via* the same reaction condition, suggesting that this reaction method can be scalable and practical.

**Scheme 4:**
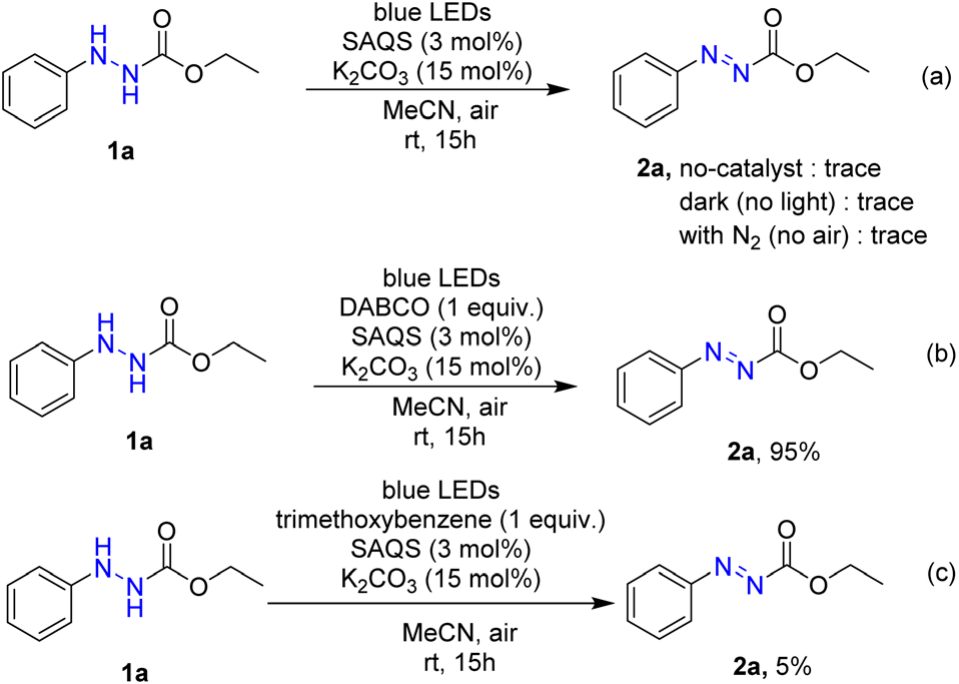
Control experiments for elucidate the reaction mechanism.

**Fig. 1:**
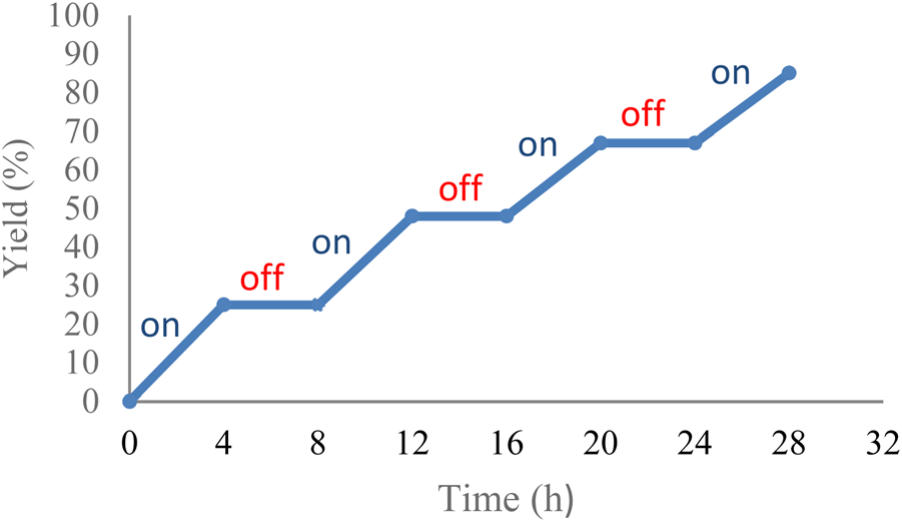
The on/off light of the reaction.

Several control experiments were carried out to better ascertain the reaction mechanism (Scheme 4). In the absence of photocatalyst, the product 2a was not synthesized. Under dark conditions, no conversion of 1a to product 2a was observed. Furthermore, a light turn on/turn off reaction was performed (Fig. 1) and indicated that light irradiation was a crucial factor for a successful transformation. The reaction was performed under N_2_, and product 2a was not produced, suggesting that oxygen affects the efficiency of the reaction. A singlet oxygen (^1^O_2_) quenching experiment was then conducted to determine whether this novel reaction followed an oxidative quenching mechanism. The singlet oxygen (^1^O_2_) quenching experiment was carried out under standard conditions with addition of 4-diazabicyclo[2.2.2]-octane (DABCO) as quencher to afford the target product in 95% yield. This suggests that ^1^O_2_ was not involved in the reaction pathway. The role of superoxide anion (O_2_˙^−^) was investigated by performing the reaction with trimethoxybenzene. In that reaction, only 5% product 2a was formed, which indicated that the reaction was inhibited by (O_2_˙^−^).

**Scheme 5:**
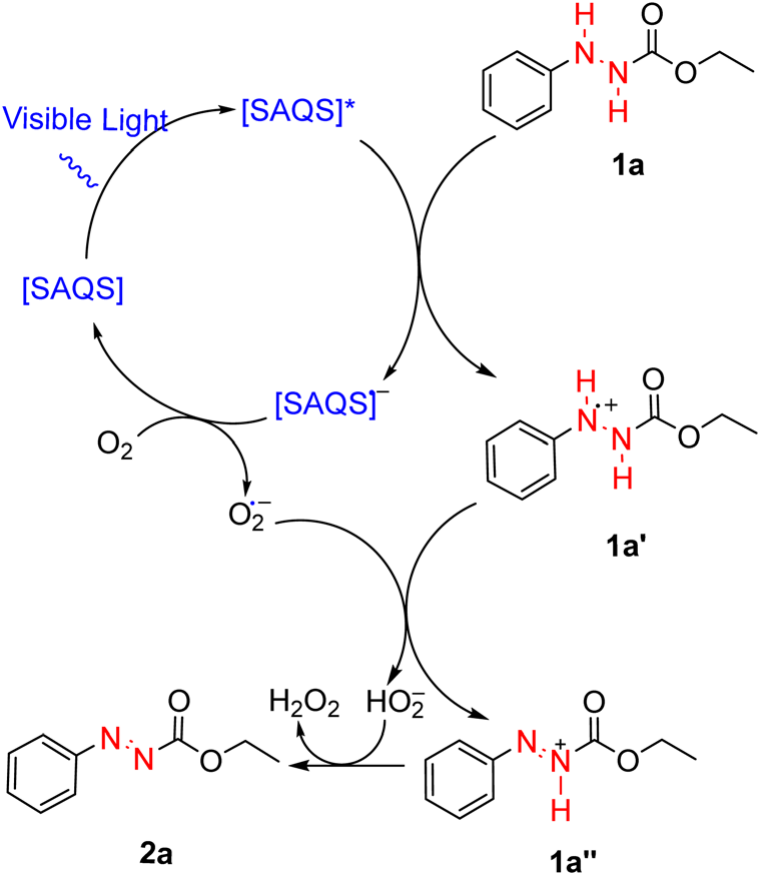
Plausible reaction mechanism.

Based on the control experiments and literature findings,^45,46^ a possible reaction mechanism is proposed in Scheme 5. Under visible light, SAQS is converted into [SAQS]*, an excited state species. [SAQS]* undergoes single electron transfer (SET) with phenylhydrazine carboxylate (1a) to produce the anion radical [SAQS]˙^−^ with concomitant generation of the radical of phenylhydrazine carboxylate (1a′). Further electron transfer between the anion radical [SAQS]˙^−^ and oxygen leads to the formation of superoxide anion (O_2_˙^−^), which allows [SAQS]˙^−^ to recover to its ground state. On the other hand, the superoxide anion abstracts one proton from (1a′) to generate the radical cation (1a′′) and peroxide radical. Finally, transfer of a proton results in conversion of the radical cation (1a′′) to the target product (2a) together with formation of H_2_O_2_.

## Conclusions

In conclusion, the visible-light-driven, photocatalyzed oxidative dehydrogenation of phenylhydrazinecarboxylates for the synthesis of azo compounds was described. In this procedure, sodium anthraquinone sulfonate, an organic molecule, was employed as an efficient photocatalyst to produce the desired products. This procedure utilizes visible light and air, suggesting that it is environmentally friendly and practical. A variety of substrates with different functional groups was tolerated in the reaction, and target phenylazocarboxylates were successfully synthesized in high yields (up to 98%) under mild reaction conditions. The novel visible-light-driven, photocatalyzed oxidative dehydrogenation of phenylazocarboxylate was an efficient approach to the synthesis of valuable azo compounds.

## Data availability

The data supporting this study are available within the article and the ESI†.

## Author contributions

H.-K. K. conceived the study. V. H. T. and H.-K. K. conducted the experiments and analysed the data. V. H. T. and H.-K. K. prepared the manuscript. V. H. T. prepared the ESI.† All authors discussed the results and commented on the manuscript.

## Conflicts of interest

There are no conflicts to declare.

## Supplementary Material

RA-012-D2RA05842A-s001
