# Supplementary material for: Visible-light-driven SAQS-catalyzed aerobic oxidative dehydrogenation of alkyl 2-phenylhydrazinecarboxylates
Source: RSC Adv. 2022 Oct 24;12(47):30304–9. doi: 10.1039/d2ra05842a (PMC9590590; doi:10.1039/d2ra05842a)
Supplement: RA-012-D2RA05842A-s001 [file RA-012-D2RA05842A-s001.pdf]

Electronic Supplementary Material (ESI) for RSC Advances.
This journal is © The Royal Society of Chemistry 2022

Supplementary Material

Visible-Light-Driven SAQS-Catalyzed Aerobic Oxidative Dehydrogenation of Alkyl 2-Phenylhydrazinecarboxylates

Van Hieu Tran [a,b] and Hee-Kwon Kim [a,b] *

[a] Department of Nuclear Medicine, Molecular Imaging & Therapeutic Medicine Research Center, Jeonbuk National University Medical School and Hospital, Jeonju, 54907, Republic of Korea

[b] Research Institute of Clinical Medicine of Jeonbuk National University-Biomedical Research Institute of Jeonbuk National University Hospital, Jeonju, 54907, Republic of Korea

* Corresponding author.
Hee-Kwon Kim: Tel: +82 63 250 2768; Fax: +82 63 255 1172.

E-mail address: hkkim717@jbnu.ac.kr (H. Kim).

Table of Contents

Table of Contents S1

General Information S2

Table S1. Screening of additives and solvents for synthesis of ethyl (E)-2-phenyldiazene-1-carboxylate S3

Table S2. Screening of amounts of reagents for synthesis of ethyl (E)-2-phenyldiazene-1-carboxylate S4

General procedure of the synthesis of azo compounds S5

Characterization of azo compounds S6

^{1}H and ^{13}C NMR Spectra..S16

General Information

All reactions were carried out in a 10-mL test tube. Commercial chemicals and solvents were used in place of the originals without any purification. The reaction was monitored using thin-layer chromatography (TLC). TLC spots were identified using an ultraviolet light (254nm) and a silica gel 60 F_{254} pre-coated aluminum plate from Merck. Flash chromatography was carried out using silica gel with mesh sizes ranging from 230 to 400 mesh and analytical grade solvents. The melting points of the products have to be recorded using a Stuart SMP10 Melting Point Apparatus. To determine the structure of molecules, NMR spectroscopy (^{1}H and ^{13}C NMR) was performed using a Bruker Avance 400 MHz spectrometer. Chemical shifts were recorded in parts per million (ppm) relative to the remaining protonated solvent resonance, coupling constants (*J*) in hertz, and signal multiplicity as follows: singlet (s); doublet (d); doublet of doublet (dd); triplet (t); multiplet (m); multiplet of multiplet (m);

Table S1. Screening of additives and solvents for synthesis of ethyl (E)-2-phenyldiazene-1-carboxylate[a]

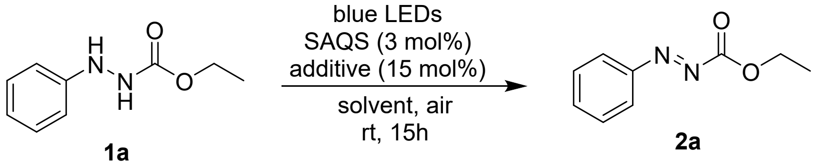

Entry	Photocatalyst	Additive	Solvent	Yield[b] (%)
1	SAQS	-	MeCN	85
2	SAQS	DIPEA	MeCN	4
3	SAQS	Et_3N	MeCN	6
4	SAQS	DMAP	MeCN	20
5	SAQS	$NaHCO_3$	MeCN	34
6	SAQS	$CsCO_3$	MeCN	65
7	SAQS	DBU	MeCN	76
8	SAQS	K_2CO_3	MeCN	97
9	SAQS	K_2CO_3	Toluene	5
10	SAQS	K_2CO_3	DCE	7
11	SAQS	K_2CO_3	CH_2Cl_2	17
12	SAQS	K_2CO_3	1,4-dioxane	19
13	SAQS	K_2CO_3	DMF	61
14	SAQS	K_2CO_3	THF	65
15[d]	SAQS	K_2CO_3	MeCN	NR[c]
16[e]	SAQS	K_2CO_3	MeCN	65
17[f]	SAQS	K_2CO_3	MeCN	70
18[g]	SAQS	K_2CO_3	MeCN	72
19[h]	SAQS	K_2CO_3	MeCN	88

[a] Reaction conditions: compound 1a (1.0 mmol), photocatalyst (0.03 mmol), Additive (0.15 mmol), solvent (2 mL), room temperature, irradiation with 5W blue LEDs for 15 h, [b] Isolated yield after purification by flash column chromatography, [c] No reaction, [d]No light, [e]irradiation with green LEDs, [f]irradiation with CFL, [g]irradiation with white LEDs, [h]irradiation with sunlight

Table S2. Screening of amounts of reagents for synthesis of ethyl (E)-2-phenyldiazene-1-carboxylate[a]

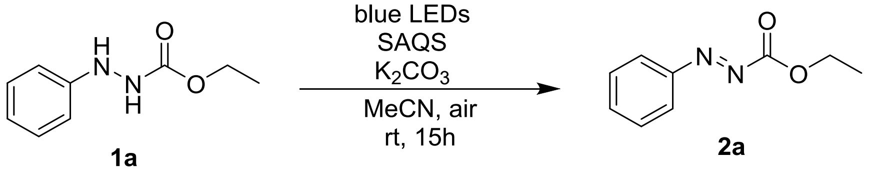

Entry	SAQS (equiv.)	K_2CO_3 (equiv.)	MeCN	Yield[b] (%)
1	0.005	0.15	2 mL	83
2	0.01	0.15	2 mL	89
3	0.03	0.15	2 mL	97
4	0.05	0.15	2 mL	97
5	0.1	0.15	2 mL	97
6	0.03	0.01	2 mL	80
7	0.03	0.03	2 mL	82
8	0.03	0.05	2 mL	85
9	0.03	0.10	2 mL	91
10	0.03	0.15	2 mL	97
11	0.03	0.20	2 mL	97

[a] Reaction conditions: compound **1a** (1.0 mmol), MeCN (2 mL), room temperature, irradiation with 5W blue LEDs for 15 h, [b] Isolated yield after purification by flash column chromatography.

1. General procedure of the synthesis of azo compounds (2a-2z, 4a-4l)

1.1. Preparation of alkyl 2-phenylazocarboxylates (2a-2z)

In a typical synthetic procedure, phenylhydrazine carboxylate (**1a**) (180 mg, 1.0 mmol), SAQS (9.3 mg, 0.03 mmol), and K_2CO_3 (20.1 mg, 0.15 mmol) were added to MeCN (2 mL). The mixture was stirred at room temperature under irradiation by 5W blue LEDs. After 15 hours, the mixture was extracted with 20 mL of CH_2Cl_2 and washed with 20 mL of water. The organic layer was dried by sodium sulfate and concentrated under reduced pressure. The residue was purified using flash column chromatography on silica gel with hexane-EtOAc as the eluent to get the intended product (**2a**) (172.66 mg, 97%).

1.2. Preparation of azobenzenes (4a-4l)

1,2-diphenylhydrazine (**3a**) (184.0 mg, 1.0 mmol), SAQS (9.3 mg, 0.03 mmol), and K_2CO_3 (20.1 mg, 0.15 mmol) were added to MeCN (2 mL). The mixture was stirred at room temperature under irradiation by 5W blue LEDs. After 15 hours, the mixture was extracted with 20 mL of CH_2Cl_2 and washed with 20 mL of water. The organic layer was dried by sodium sulfate and concentrated under reduced pressure. The residue was purified using flash column chromatography on silica gel with hexane-EtOAc as the eluent to get the intended product (**4a**) as yellow solid (173.0 mg, 95 %).

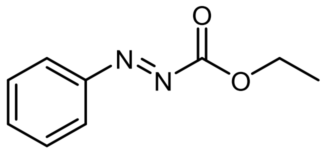

Ethyl (*E*)-2-phenyldiazene-1-carboxylate (2a): Yellow oil.

^{1}H NMR (400 MHz, CDCl$_3$) δ 7.94 (d, J = 8.4 Hz, 2H), 7.60 – 7.51 (m, 3H), 4.53 (q, J = 7.6 Hz, 2H), 1.47 (t, J = 7.2 Hz, 3H).

^{13}C NMR (100 MHz, CDCl$_3$) δ 162.2, 151.6, 133.8, 129.3 (2C), 123.8 (2C), 64.5, 14.2.

HRMS (ESI) m/z (M+H)$^+$ calcd for $C_9H_{11}N_2O_2$ = 179.0821, found 179.0823.

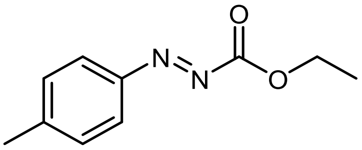

Ethyl (E)-2-(*p*-tolyl)diazene-1-carboxylate (2b): Yellow oil.

^{1}H NMR (400 MHz, CDCl$_3$) δ 7.86 – 8.83 (dt, J = 6.8 Hz, J = 2.0 Hz, 2H), 7.33 (d, J = 8.4 Hz, 2H), 4.52 (q, J = 7.2 Hz, 2H), 2.44 (s, 3H), 1.47 (t, J = 7.2 Hz, 3H).

^{13}C NMR (100 MHz, CDCl$_3$) δ 162.2, 149.9, 145.2, 129.9 (2C), 123.9 (2C), 64.4, 21.8, 14.2.

HRMS (ESI) m/z (M+H)$^+$ calcd for $C_{10}H_{13}N_2O_2$ = 193.0977, found 193.0978.

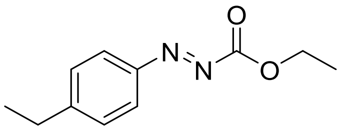

Ethyl (E)-2-(4-ethylphenyl)diazene-1-carboxylate (2c): Yellow oil.

^{1}H NMR (400 MHz, CDCl$_3$) δ 7.88 – 7.86 (dd, J = 6.4 Hz, J = 1.6 Hz 2H), 7.36 (d, J = 8.8 Hz, 2H), 4.52 (q, J = 6.8 Hz, 2H), 2.75 (q, J = 7.6 Hz, 2H), 1.47 (t, J = 6.8 Hz, 3H), 1.28 (t, J = 7.6 Hz, 3H).

^{13}C NMR (100 MHz, CDCl$_3$) δ 162.2, 151.3, 150.0, 128.8 (2C), 124.1 (2C), 64.4, 29.0, 15.1, 14.2.

HRMS (ESI) m/z (M+H)$^+$ calcd for $C_{11}H_{15}N_2O_2$ = 207.1134, found 207.1137.

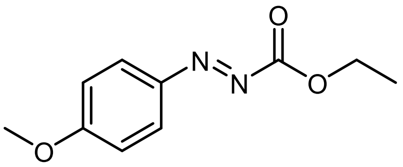

Ethyl (E)-2-(4-methoxyphenyl)diazene-1-carboxylate (2d): Yellow oil.

^{1}H NMR (400 MHz, CDCl$_3$) δ 7.98 – 7.95 (dt, J = 9.2 Hz, J = 2.4 Hz, 2H), 7.02 – 6.98 (dt, J = 8.8 Hz, J = 2.0 Hz, 2H), 4.52 (q, J = 7.2 Hz, 2H), 3.91 (s, 3H), 1.47 (t, J = 7.2 Hz, 3H).

^{13}C NMR (100 MHz, CDCl$_3$) δ 164.7, 162.1, 146.1, 126.5 (2C), 114.5 (2C), 64.2, 55.8, 14.2.

HRMS (ESI) m/z $(M+H)^+$ calcd for $C_{10}H_{13}N_2O_3$ = 209.0926, found 209.0925.

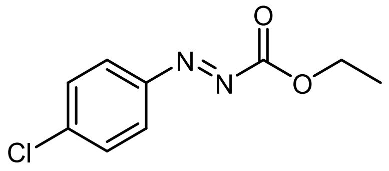

Ethyl (E)-2-(4-chlorophenyl)diazene-1-carboxylate (2e): Yellow oil.

^{1}H NMR (400 MHz, CDCl$_3$) δ 7.90- 7.86 (dt, J = 8.8 Hz, J = 2.0 Hz, 2H), 7.54 (dt, J = 8.4 Hz, J = 8.4 Hz, 2H), 4.53 (q, J = 7.2 Hz, 2H), 1.47 (t, J = 7.2 Hz, 3H).

^{13}C NMR (100 MHz, CDCl$_3$) δ 161.9, 149.9, 140.2, 129.7 (2C), 125.0 (2C), 64.6, 14.2.

HRMS (ESI) m/z $(M+H)^+$ calcd for $C_9H_{10}ClN_2O_2$ = 213.0431, found 213.0428.

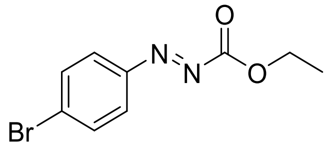

Ethyl (E)-2-(4-bromophenyl)diazene-1-carboxylate (2f): Yellow oil.

^{1}H NMR (400 MHz, CDCl$_3$) δ 7.82 – 7.78 (dt, J = 8.8 Hz, J = 2.0 Hz, 2H), 7.69 – 7.66 (dt, J = 8.8 Hz, J = 2.0 Hz, 2H), 4.53 (q, J = 7.2 Hz, 2H), 1.47 (t, J = 7.2 Hz, 3H).

^{13}C NMR (100 MHz, CDCl$_3$) δ 161.9, 150.3, 132.7 (2C), 128.9, 125.14 (2C), 64.6, 14.2.

HRMS (ESI) m/z $(M+H)^+$ calcd for $C_9H_{10}BrN_2O_2$ = 256.9926, found 256.9927.

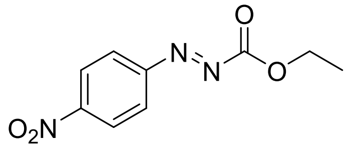

Ethyl (E)-2-(4-nitrophenyl)diazene-1-carboxylate (2g): Yellow oil.

^{1}H NMR (400 MHz, CDCl$_3$) δ 8.42 – 8.38 (dt, J = 8.8 Hz, J = 2.0 Hz 2H), 8.06 – 8.04 (dt, J = 8.8 Hz, J = 2.0 Hz 2H), 4.56 (q, J = 7.2 Hz, 2H), 1.51 (t, J = 6.8 Hz, 3H).

^{13}C NMR (100 MHz, CDCl$_3$) δ 161.6, 154.3, 150.4, 124.9 (2C), 124.3 (2C), 65.1, 14.2.

HRMS (ESI) m/z $(M+H)^+$ calcd for $C_9H_{10}N_3O_4$ = 224.0671, found 224.0674.

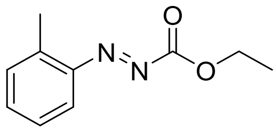

Ethyl (E)-2-(o-tolyl)diazene-1-carboxylate (2h): Yellow oil.

^{1}H NMR (400 MHz, CDCl$_3$) δ 7.58 (d, J = 8.0 Hz, 1H), 7.47 – 7.44 (td, J = 7.6 Hz, J = 1.2 Hz, 1H), 7.37 (d, J = 7.6 Hz, 1H), 7.24 (t, J = 7.2 Hz, 1H), 4.52 (q, J = 7.2 Hz, 2H), 2.69 (s, 1H), 1.47 (t, J = 7.2 Hz, 3H).

^{13}C NMR (100 MHz, CDCl$_3$) δ 162.6, 149.9, 140.69, 133.7, 131.6, 126.4, 115.27, 64.3, 17.5, 14.2.

HRMS (ESI) m/z (M+H)$^+$ calcd for $C_{10}H_{13}N_2O_2$ = 193.0977, found 193.0979.

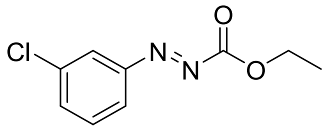

Ethyl (E)-2-(3-chlorophenyl)diazene-1-carboxylate (2i): Yellow oil.

^{1}H NMR (400 MHz, CDCl$_3$) δ 7.89 – 7.84 (m, 2H), 7.57 – 7.55 (m, 1H), 7.49 (t, J = 7.6 Hz, 1H), 4.53 (q, J = 7.2 Hz, 2H), 1.47 (t, J = 7.2 Hz, 3H).

^{13}C NMR (100 MHz, CDCl$_3$) δ 161.8, 152,3, 135.5, 133.5, 130.4, 123.4, 122.4, 64.7, 14.2.

HRMS (ESI) m/z (M+H)$^+$ calcd for $C_9H_{10}ClN_2O_2$ = 213.0431, found 213.0435.

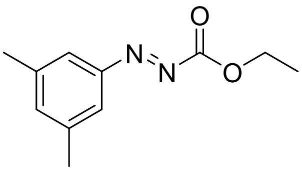

Ethyl (E)-2-(3,5-dimethylphenyl)diazene-1-carboxylate (2j): Yellow oil.

^{1}H NMR (400 MHz, CDCl$_3$) δ 7.56 (s, 2H), 7.22 (s, 1H), 4.52 (q, J = 7.2 Hz, 2H), 2.39 (s, 6H), 1.47 (t, J = 7.2 Hz, 3H).

^{13}C NMR (100 MHz, CDCl$_3$) δ 162.2, 151.9, 139.1 (2C), 135.6, 121.6 (2C), 64.4, 21.1 (2C), 14.2.

HRMS (ESI) m/z (M+H)$^+$ calcd for $C_{11}H_{15}N_2O_2$ = 207.1134, found 207.1136.

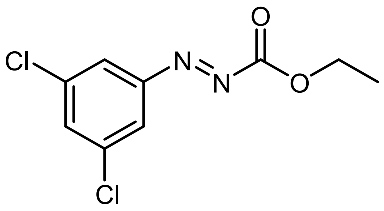

Ethyl (*E*)-2-(3,5-dichlorophenyl)diazene-1-carboxylate (2k): Yellow oil.

^{1}H NMR (400 MHz, CDCl$_3$) δ 7.81 (d, J = 2.0 Hz, 2H), 7.56 (t, J = 2.0 Hz, 1H), 4.53 (q, J = 7.2 Hz, 2H), 1.47 (t, J = 7.2 Hz, 3H).

^{13}C NMR (100 MHz, CDCl$_3$) δ 161.5, 152.5, 136.1 (2C), 132.9, 122.2 (2C), 64.9, 14.2.

HRMS (ESI) m/z (M+H)$^+$ calcd for $C_9H_9Cl_2N_2O_2$ = 247.0041, found 247.0042.

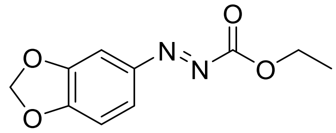

Ethyl (E)-2-(benzo[d][1,3]dioxol-5-yl)diazene-1-carboxylate (2l): Yellow oil.

^{1}H NMR (400 MHz, CDCl$_3$) δ 6.73 – 6.65 (m, 2H), 6.46 (s, 1H), 5.93 (s, 2H), 4.31(q, J = 7.2 Hz, 2H), 1.29 (t, J = 7.2 Hz, 3H).

^{13}C NMR (100 MHz, CDCl$_3$) δ 153.8, 147.9, 143.7, 132.2, 111.9, 108.1, 101.9, 101.2, 61.2, 14.6.

HRMS (ESI) m/z (M+H)$^+$ calcd for $C_{10}H_{11}N_2O_4$ = 223.0719, found 223.0716.

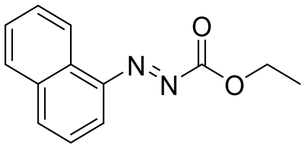

Ethyl (E)-2-(naphthalen-1-yl)diazene-1-carboxylate (2m): Yellow oil.

^{1}H NMR (400 MHz, CDCl$_3$) δ 8.83 (d, J = 8.0 Hz, 1H), 8.11 (d, J = 8.4 Hz, 1H), 7.94 (d, J = 8.0 Hz, 1H), 7.84 – 7.82 (dd, J = 7.6 Hz, J = 1.2 Hz, 1H), 7.69 -7.65 (m, 1H), 7.63 – 7.59 (m, 1H), 7.55 (t, J = 7.6 Hz, 1H), 4.57 (q, J = 7.2 Hz, 2H), 1.51 (t, J = 7.2 Hz, 3H).

^{13}C NMR (100 MHz, CDCl$_3$) δ 157.1, 142.6 (2C), 134.1, 128.6, 126.0, 125.9, 125.3, 123.0, 121.3, 119.8, 62.0, 14.5.

HRMS (ESI) m/z (M+H)$^+$ calcd for $C_{13}H_{13}N_2O_2$ = 229.0977, found 229.0975.

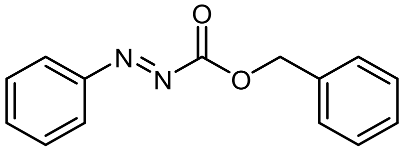

Benzyl (E)-2-phenyldiazene-1-carboxylate (2n): Yellow oil.

^{1}H NMR (400 MHz, CDCl$_3$) δ 7.95 – 7.92 (m, 2H), 7.61 – 7.48 (m, 5H), 7.44 – 7.36 (m, 3H), 5.49 (s, 2H).

^{13}C NMR (100 MHz, CDCl$_3$) δ 162.1, 151.6, 134.4, 133.9, 129.32 (2C), 128.9, 128.8 (2C), 128.7 (2C), 123.9 (2C), 69.9.

HRMS (ESI) m/z $(M+H)^+$ calcd for $C_{14}H_{13}N_2O_2$ = 241.0977, found 241.0974.

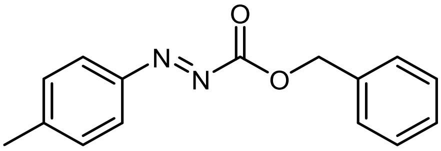

Benzyl (E)-2-(*p*-tolyl)diazene-1-carboxylate (2o): Yellow oil.

^{1}H NMR (400 MHz, CDCl$_3$) δ 7.85 (d, J = 8.4 Hz, 2H), 7.50 – 7.48 (dd, J = 8.0 Hz, J = 1.2 Hz, 2H), 7.43 – 7.36 (m, 5H), 5.47 (s, 2H), 2.44 (s, 3H).

^{13}C NMR (100 MHz, CDCl$_3$) δ 162.1, 149.9, 145.33, 134.5, 130.0 (2C), 128.8, 128.8 (2C), 128.7 (2C), 124.0 (2C), 69.8. 21.8.

HRMS (ESI) m/z $(M+H)^+$ calcd for $C_{15}H_{15}N_2O_2$ = 255.1134, found 255.1136.

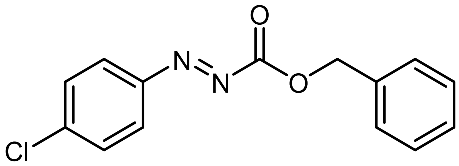

Benzyl (*E*)-2-(4-chlorophenyl)diazene-1-carboxylate (2p): Yellow oil.

^{1}H NMR (400 MHz, CDCl$_3$) δ 7.89 – 7.86 (dt, J = 6.8 Hz, J = 2.4 Hz, 2H), 7.52 – 7.48 (m, 4H), 7.43 – 7.38 (m, 3H), 5.47 (s, 2H).

^{13}C NMR (100 MHz, CDCl$_3$) δ 161.8, 149.9, 140.3, 134.3, 129.7 (2C), 128.9, 128.8 (2C), 128.8 (2C), 125.1 (2C), 70.1.

HRMS (ESI) m/z $(M+H)^+$ calcd for $C_{14}H_{12}ClN_2O_2$ = 275.0587, found 275.0588.

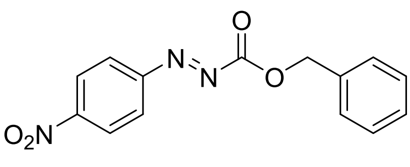

Benzyl (E)-2-(4-nitrophenyl)diazene-1-carboxylate (2q): Yellow oil.

^{1}H NMR (400 MHz, CDCl$_3$) δ 8.41 – 8.37 (dt, J = 8.8 Hz, J = 2.0 Hz, 2H), 8.06 -8.03 (dt, J = 8.8 Hz, J = 2.0 Hz, 2H), 7.50 – 7.47 (m, 2H), 7.45 – 7.38 (m, 3H), 5.49 (s, 2H).

^{13}C NMR (100 MHz, CDCl$_3$) δ 161.5, 154.3, 150.4, 133.9, 129.2, 128.8 (4C), 124.8 (2C), 124.3 (2C), 70.5.

HRMS (ESI) m/z (M+H)$^+$ calcd for $C_{14}H_{12}N_3O_4$ = 286.0828, found 286.0830.

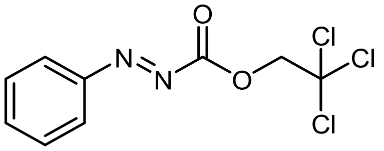

2,2,2-trichloroethyl (E)-2-phenyldiazene-1-carboxylate (2r): Yellow oil.

^{1}H NMR (400 MHz, CDCl$_3$) δ 8.01 – 7.98 (m, 2H), 7.65 -7.61 (m, 1H), 7.58 – 7.54 (m, 2H), 5.07 (s, 2H).

^{13}C NMR (100 MHz, CDCl$_3$) δ 160.7, 151.7, 134.6, 129.4 (2C), 124.2 (2C), 93.9, 76.5.

HRMS (ESI) m/z (M+H)$^+$ calcd for $C_9H_8Cl_3N_2O_2$ = 280.9651, found 280.9653.

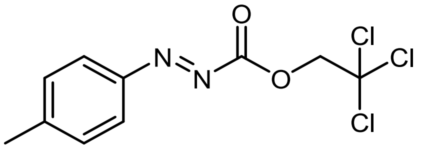

2,2,2-trichloroethyl (E)-2-(p-tolyl)diazene-1-carboxylate (2s): Yellow oil.

^{1}H NMR (400 MHz, CDCl$_3$) δ 7.92 (d, J = 8.4 Hz, 2H), 7.36 (d, J = 8.0 Hz, 2H), 5.06 (s, 2H), 2.47 (s, 3H).

^{13}C NMR (100 MHz, CDCl$_3$) δ 160.7, 153.2, 146.2, 130.1 (2C), 124.4 (2C), 93.9, 76.5, 21.9.

HRMS (ESI) m/z (M+H)$^+$ calcd for $C_{10}H_{10}Cl_3N_2O_2$ = 294.9808, found 294.9807.

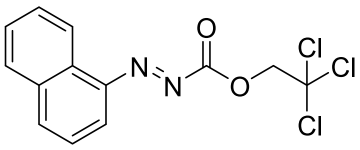

2,2,2-trichloroethyl (E)-2-(naphthalen-1-yl)diazene-1-carboxylate (2t): Yellow oil.

^{1}H NMR (400 MHz, CDCl$_3$) δ 8.85 (d, J = 84 Hz, 1H), 8.16 (d, J = 8.8 Hz, 1H), 7.96 – 7.92 (m, 2H), 7.72 – 7.68 (m, 1H), 7.65 - 7.61 (m, 1H), 7.58 (t, J = 8.0 Hz, 1H), 5.11 (s, 2H.

^{13}C NMR (100 MHz, CDCl$_3$) δ 153.5, 135.5, 128.2, 128.1, 127.8 (2C), 127.1, 125.8, 125.2, 123.2, 113.1, 93.9, 76.5.

HRMS (ESI) m/z (M+H)$^+$ calcd for $C_{13}H_{10}Cl_3N_2O_2$ = 330.9808, found 330.9805.

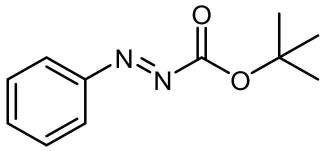

***tert*-butyl (E)-2-phenyldiazene-1-carboxylate (2u):** Yellow oil.

^{1}H NMR (400 MHz, CDCl$_3$) δ 7.92 – 7.89 (m, 2H), 7.58 – 7.49 (m, 3H), 1.66 (s, 9H).

^{13}C NMR (100 MHz, CDCl$_3$) δ 161.2, 146.7, 133.4, 129.2 (2C), 123.6 (2C), 85.0, 27.9 (3C).

HRMS (ESI) m/z $(M+H)^+$ calcd for $C_{11}H_{15}N_2O_2$ = 207.1134, found 207.1136.

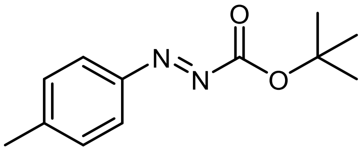

***tert*-butyl (*E*)-2-(*p*-tolyl)diazene-1-carboxylate (2v):** Yellow oil.

^{1}H NMR (400 MHz, CDCl$_3$) δ 7.82 (d, J = 8.4 Hz, 2H), 7.31 (d, J = 8.0 Hz, 2H), 2.43 (m, 3H), 1.65 (s, 9H).

^{13}C NMR (100 MHz, CDCl$_3$) δ 161.3, 146.8, 144.6, 129.9 (2C), 123.7 (2C), 84.8, 27.9 (3C), 21.7.

HRMS (ESI) m/z $(M+H)^+$ calcd for $C_{12}H_{17}N_2O_2$ = 221.1290, found 221.1293.

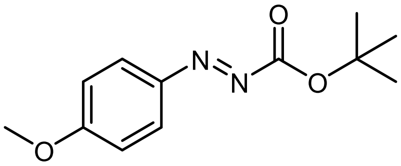

***tert*-butyl (*E*)-2-(4-methoxyphenyl)diazene-1-carboxylate (2w):** Yellow oil.

^{1}H NMR (400 MHz, CDCl$_3$) δ 7.94 – 7.91 (dt, J = 9.2 Hz, J = 2.0 Hz, 2H), 7.00 – 6.97 (dt, J = 9.2 Hz, J = 2.0 Hz, 2H), 3.89 (m, 3H), 1.66 (s, 9H).

^{13}C NMR (100 MHz, CDCl$_3$) δ 164.2, 161.2, 146.0, 126.2 (2C), 114.4 (2C), 84.4, 55.7, 27.9 (3C).

HRMS (ESI) m/z $(M+H)^+$ calcd for $C_{12}H_{17}N_2O_3$ = 237.1239, found 237.1236.

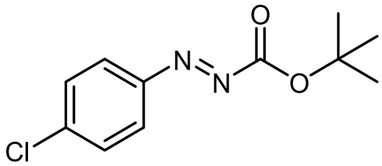

***tert*-butyl (*E*)-2-(4-chlorophenyl)diazene-1-carboxylate (2x):** Yellow oil.

^{1}H NMR (400 MHz, CDCl$_3$) δ 7.87 – 7.84 (dt, J = 8.8 Hz, J = 2.0 Hz, 2H), 7.50 – 7.48 (dt, J

= 8.8 Hz, *J* = 2.0 Hz, 2H), 1.66 (s, 9H).

^{13}C NMR (100 MHz, $CDCl_3$) δ 160.9, 149.9, 139.7, 129.6 (2C), 124.9 (2C), 85.3, 27.9 (3C).

HRMS (ESI) m/z $(M+H)^+$ calcd for $C_{11}H_{14}ClN_2O_2$ = 241.0744, found 241.0746.

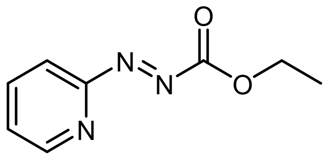

Ethyl (E)-2-(pyridin-2-yl)diazene-1-carboxylate (2y): Colorless oil.

^{1}H NMR (400 MHz, $CDCl_3$) δ 8.39 (d, *J* = 3.6 Hz, 1H), 7.81 (d, *J* = 7.2 Hz, 1H), 7.71 (t, *J* = 6.8 Hz, 1H), 7.12 – 7.09 (dt, *J* = 4.8 Hz, *J* = 1.2 Hz, 1H), 4.33 (q, *J* = 7.2 Hz, 2H), 1.32 (t, *J* = 7.2 Hz, 3H).

^{13}C NMR (100 MHz, $CDCl_3$) δ 156.1, 154.5, 153.0, 147.7, 137.9, 120.9, 62.2, 14.4.

HRMS (ESI) m/z $(M+H)^+$ calcd for $C_8H_{10}N_3O_2$ = 180.0773, found 180.0776.

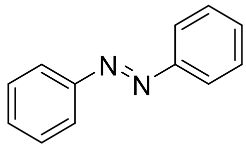

(E)-1,2-diphenyldiazene (4a): Yellow solid. m.p. 66 -68 °C.

^{1}H NMR (400 MHz, $CDCl_3$) δ 7.94 – 7.91 (dt, *J* = 8.0 Hz, *J* = 2.0 Hz, 4H), 7.55 – 7.46 (m, 6H).

^{13}C NMR (100 MHz, $CDCl_3$) δ 152.6 (2C), 131.0 (2C), 129.1 (4C), 122.9 (4C).

HRMS (ESI) m/z $(M+H)^+$ calcd for $C_{12}H_{11}N_2$ = 183.0922, found 183.0921.

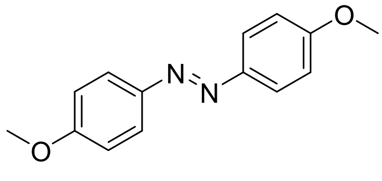

(E)-1,2-bis(4-methoxyphenyl)diazene (4b): Yellow solid. m.p. 160 -162 °C.

^{1}H NMR (400 MHz, $CDCl_3$) δ 7.94 (d, *J* = 8.8 Hz, 4H), 7.04 (d, *J* = 9.2 Hz, 4H), 3.91 (s, 6H).

^{13}C NMR (100 MHz, $CDCl_3$) δ 161.7 (2C), 146.9 (2C), 124.5 (4C), 114.2 (4C), 55.6 (2C).

HRMS (ESI) m/z $(M+H)^+$ calcd for $C_{14}H_{15}N_2O_2$ = 243.1134, found 243.1138.

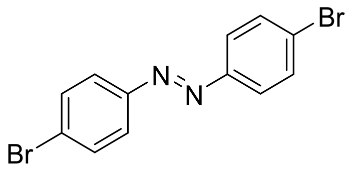

(E)-1,2-bis(4-bromophenyl)diazene (4c): Yellow solid. m.p. 203 - 205 °C.

^{1}H NMR (400 MHz, $CDCl_3$) δ 7.81 – 7.78 (dt, J = 8.4 Hz, J = 2.0 Hz, 4H), 7.66 – 7.64 (dt, J = 8.8 Hz, J = 2.0 Hz, 4H).

^{13}C NMR (100 MHz, $CDCl_3$) δ 151.2 (2C), 132.4 (4C), 125.8 (2C), 124.4 (4C).

HRMS (ESI) m/z $(M+H)^+$ calcd for $C_{12}H_9Br_2N_2$ = 338.9132, found 338.9129.

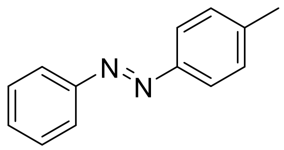

(E)-1-phenyl-2-(p-tolyl)diazene (4d): Yellow solid. m.p. 68 - 70 °C.

^{1}H NMR (400 MHz, DMSO d_6) δ 7.89 – 7.87 (m, 2H), 7.83 (d, J = 8.4 Hz, 2H), 7.62 – 7.56 (m, 3H), 7.42 (d, J = 7.6 Hz, 2H), 2.42 (s, 3H).

^{13}C NMR (100 MHz, DMSO d_6) δ 150.5, 148.1, 129.7 (2C), 129.2 (2C), 126.6, 117.9, 112.3 (2C), 112.1 (2C), 20.6.

HRMS (ESI) m/z $(M+H)^+$ calcd for $C_{13}H_{13}N_2$ = 197.1079, found 197.1076.

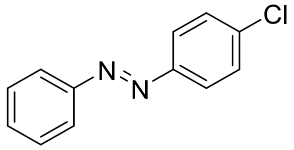

(E)-1-(4-chlorophenyl)-2-phenyldiazene (4e): Yellow oil. m.p. 87 - 89 °C.

^{1}H NMR (400 MHz, $CDCl_3$) δ 7.93 – 7.87 (m, 4H), 7.45 – 7.48 (m, 5H).

^{13}C NMR (100 MHz, $CDCl_3$) δ 152.4, 150.9, 136.9, 131.3, 129.4 (2C), 129.2 (2C), 124.1 (2C), 122.9 (2C).

HRMS (ESI) m/z $(M+H)^+$ calcd for $C_{12}H_{10}ClN_2$ = 217.0533, found 217.0535.

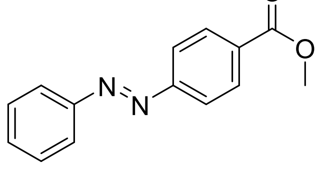

Methyl (E)-4-(phenyldiazenyl)benzoate (4f): Yellow solid. m.p. 123 -125 °C.

^{1}H NMR (400 MHz, $CDCl_3$) δ 8.21 – 8.18 (dt, J = 8.8 Hz, J = 2.0 Hz, 2H), 7.96 – 7.94 (dt, J = 6.0 Hz, J = 1.6 Hz, 4H), 7.56 – 7.49 (m, 3H), 3.96 (s, 3H).

^{13}C NMR (100 MHz, CDCl$_3$) δ 166.6, 155.1, 152.6, 131.8, 131.7, 130.6 (2C), 129.2 (2C), 123.2 (2C), 122.6 (2C), 52.4.

HRMS (ESI) m/z $(M+H)^+$ calcd for $C_{14}H_{13}N_2O_2$ = 241.0977, found 241.0979.

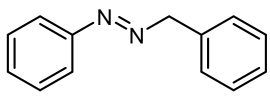

(E)-1-benzyl-2-phenyldiazene (6a): Yellow oil.

^{1}H NMR (400 MHz, CDCl$_3$) δ 7.40 - 7.28 (m, 5H), 7.19 (t, J = 8.4 Hz, 2H), 6.76 (t, J = 7.2 Hz, 1H), 6.69 (d, J = 7.6 Hz, 2H), 4.34 (s, 2H).

^{13}C NMR (100 MHz, CDCl$_3$) δ 147.2, 138.8, 129.3 (2C), 128.6 (2C), 127.8 (2C), 127.4, 126.2, 118.4, 113.6, 48.9.

HRMS (ESI) m/z $(M+H)^+$ calcd for $C_{13}H_{13}N_2$ = 197.1079, found 197.1082.

Ethyl (*E*)-2-phenyldiazene-1-carboxylate (2a)

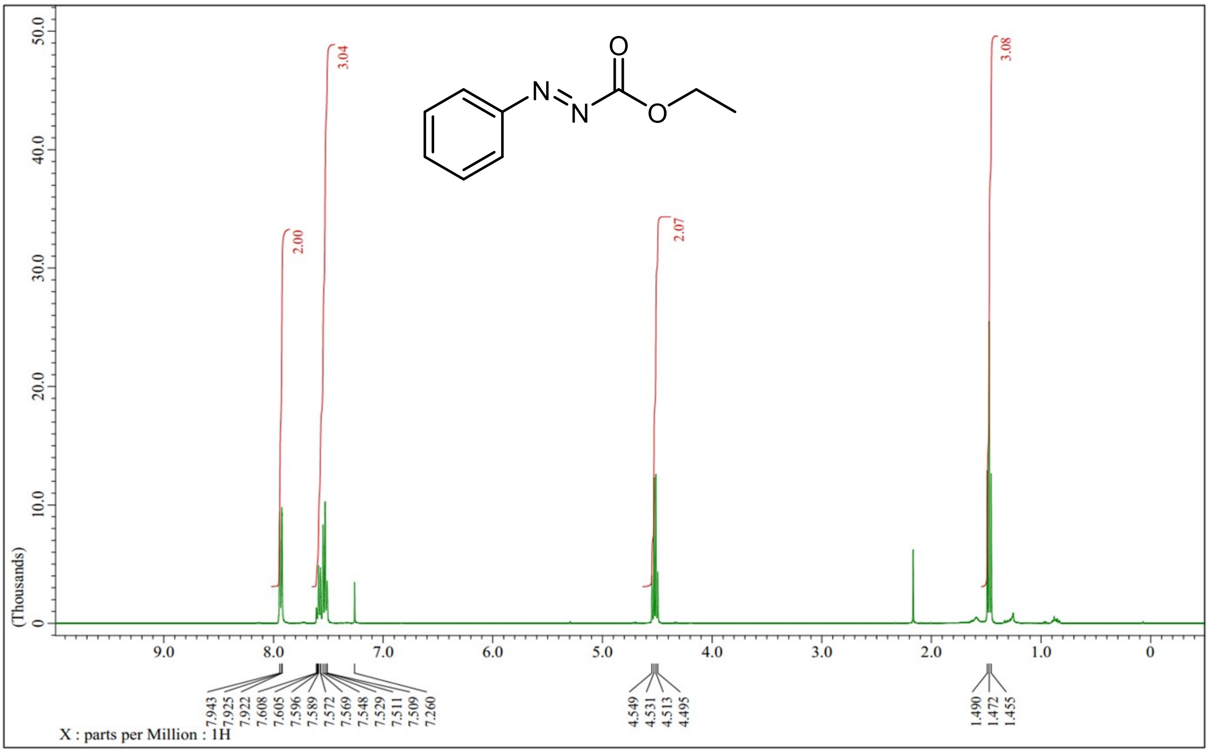

^{1}H NMR spectrum of ethyl (*E*)-2-phenyldiazene-1-carboxylate (**2a**)

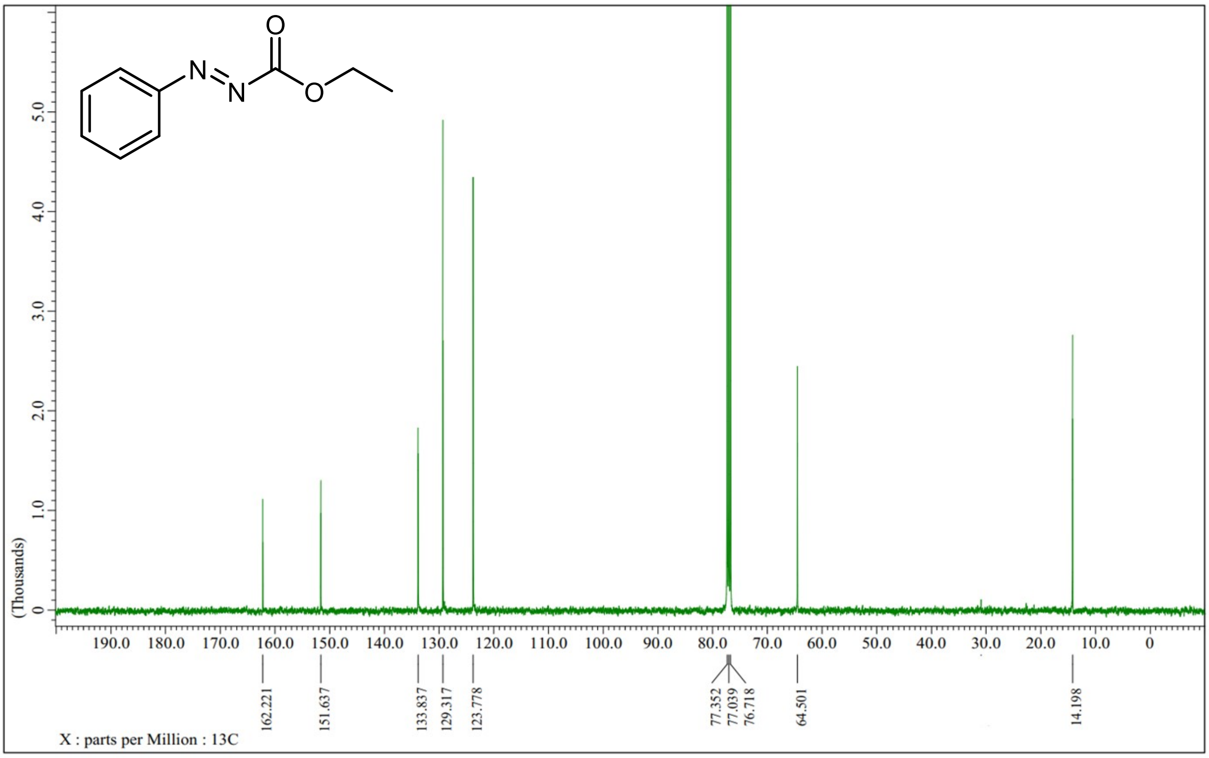

^{13}C NMR spectrum of ethyl (*E*)-2-phenyldiazene-1-carboxylate (**2a**)

Ethyl (E)-2-(p-tolyl)diazene-1-carboxylate (2b)

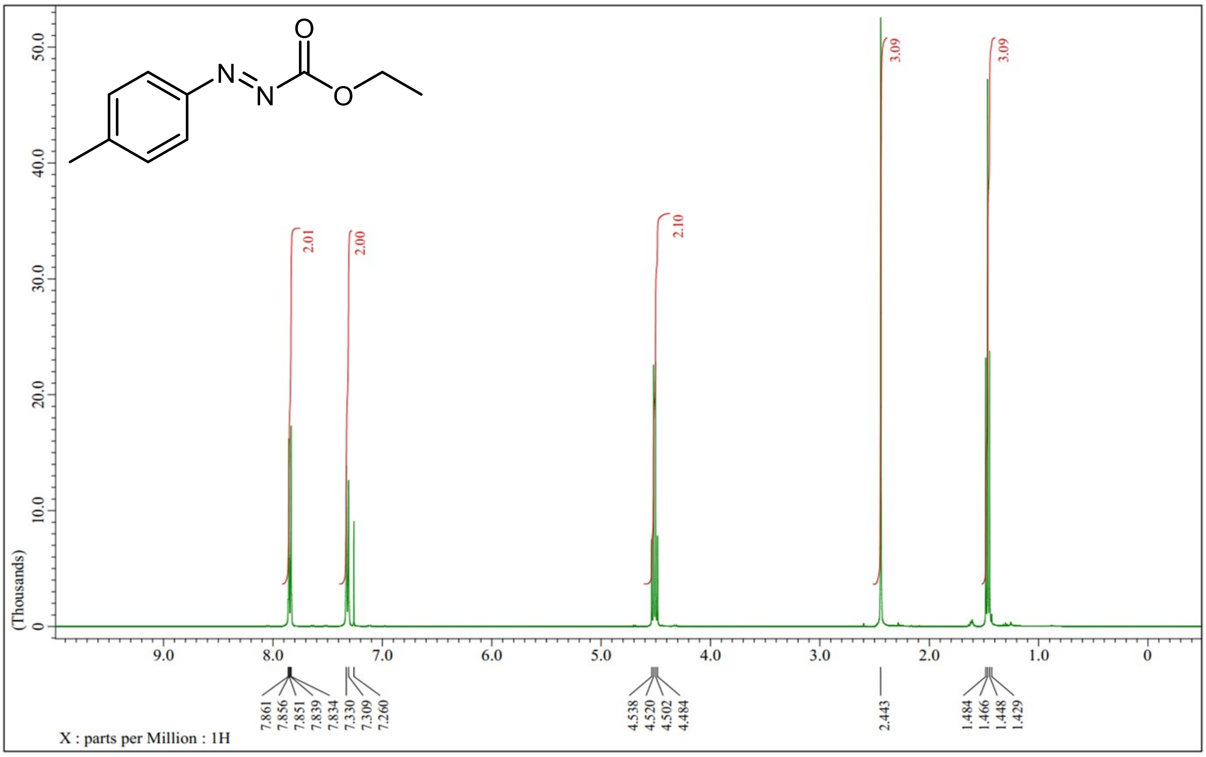

^{1}H NMR spectrum of ethyl (E)-2-(*p*-tolyl)diazene-1-carboxylate (**2b**)

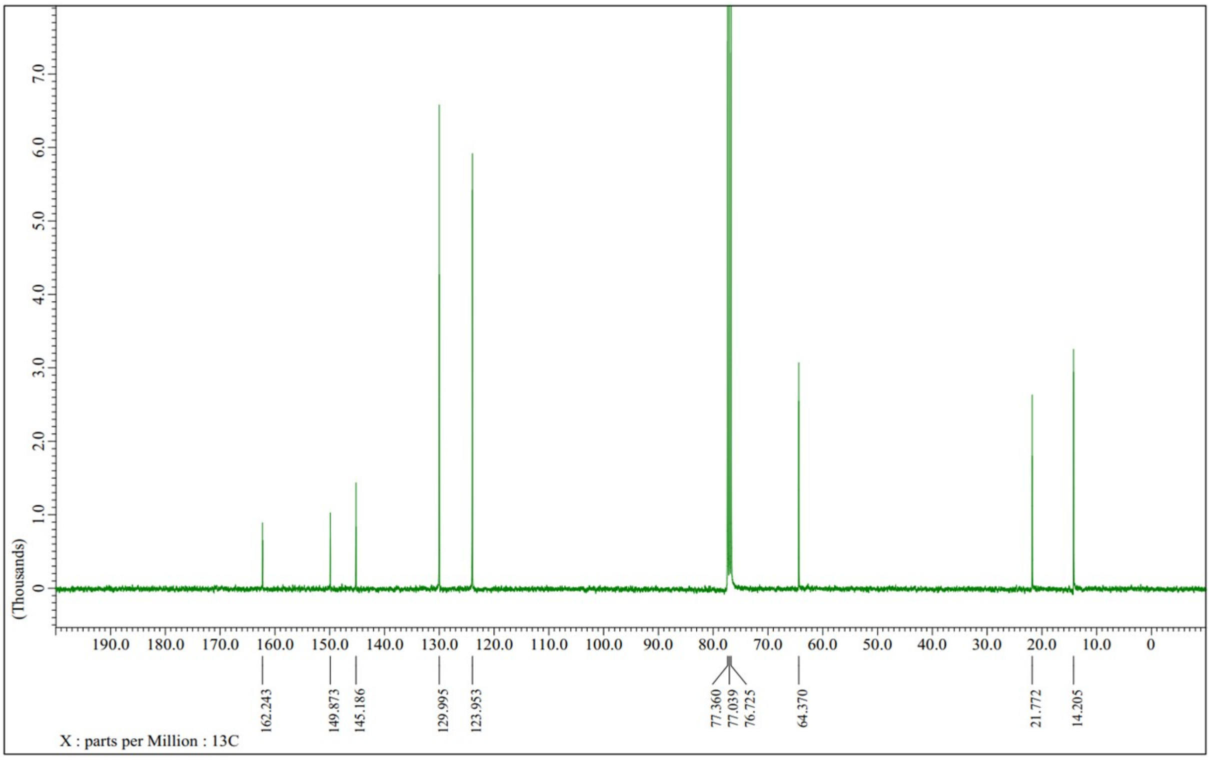

^{13}C NMR spectrum of ethyl (E)-2-(*p*-tolyl)diazene-1-carboxylate (**2b**)

Ethyl (E)-2-(4-ethylphenyl)diazene-1-carboxylate (2c)

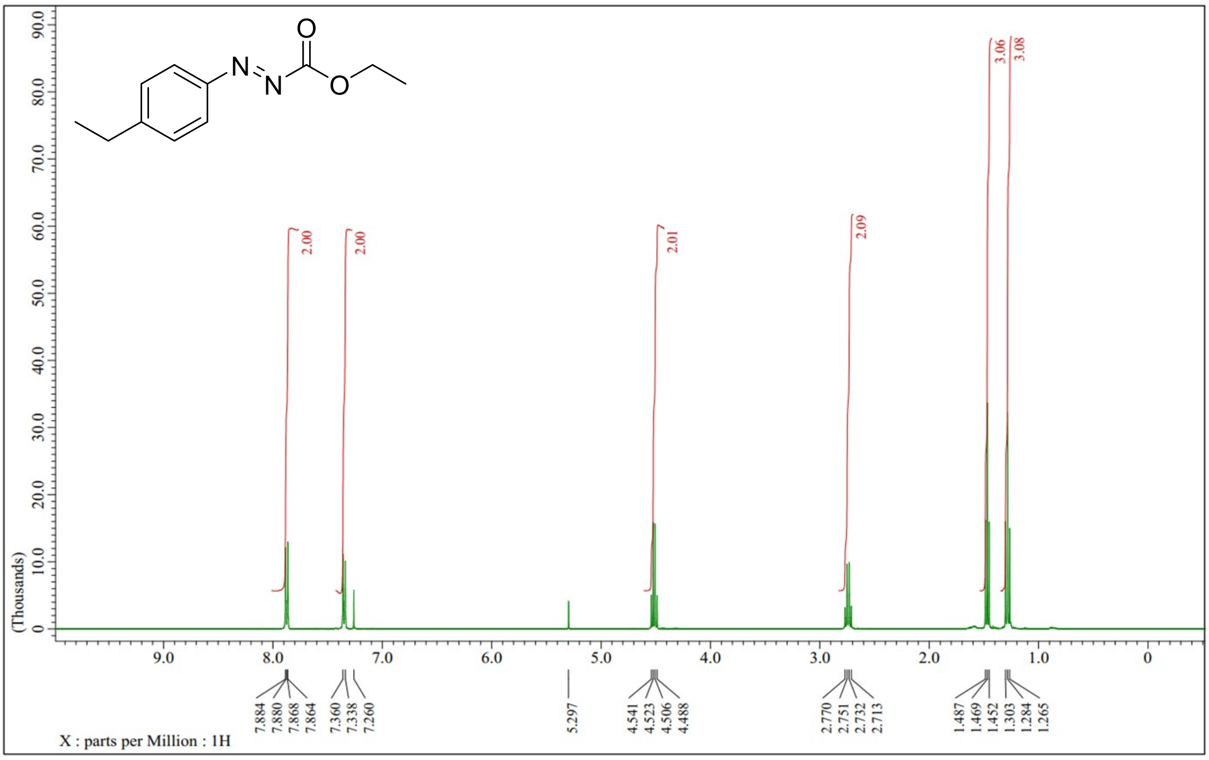

^{1}H NMR spectrum of ethyl (E)-2-(4-ethylphenyl)diazene-1-carboxylate (**2c**)

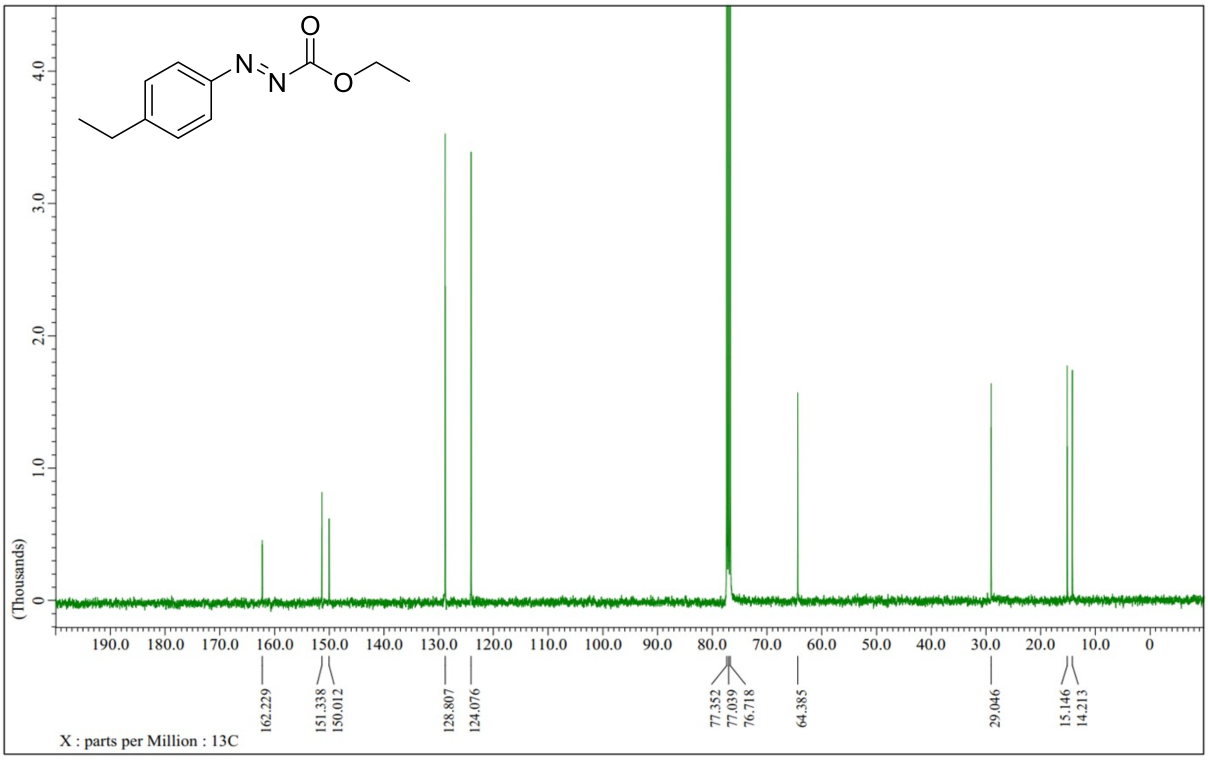

^{13}C NMR spectrum of ethyl (E)-2-(4-ethylphenyl)diazene-1-carboxylate (**2c**)

Ethyl (E)-2-(4-methoxyphenyl)diazene-1-carboxylate (2d)

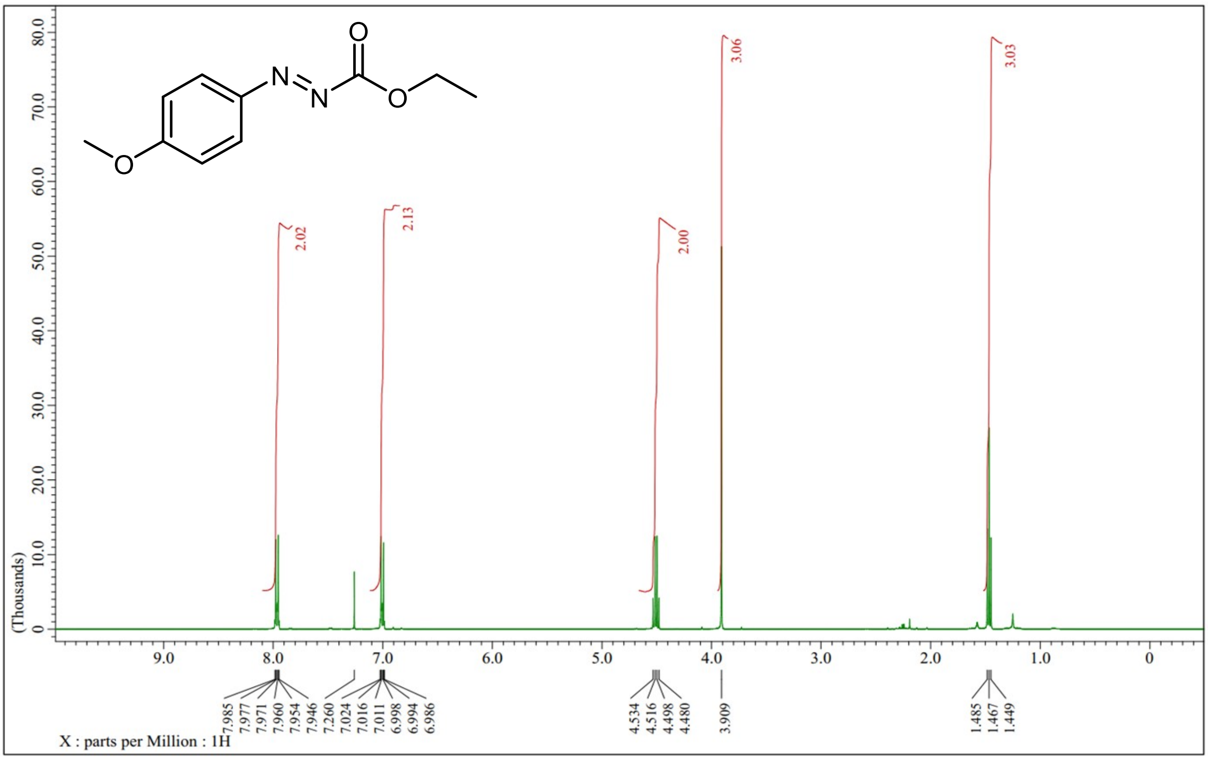

^1H NMR spectrum of ethyl (E)-2-(4-methoxyphenyl)diazene-1-carboxylate (**2d**)

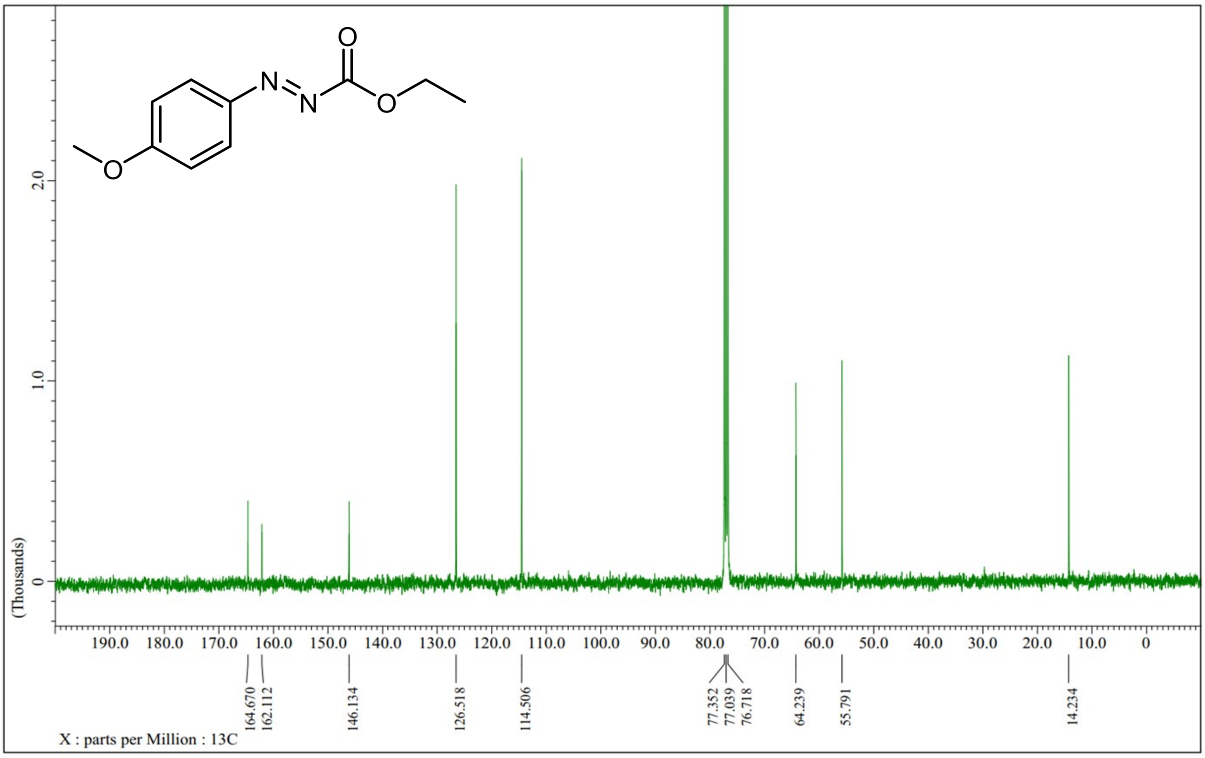

^{13}C NMR spectrum of ethyl (E)-2-(4-methoxyphenyl)diazene-1-carboxylate (**2d**)

Ethyl (E)-2-(4-chlorophenyl)diazene-1-carboxylate (2e)

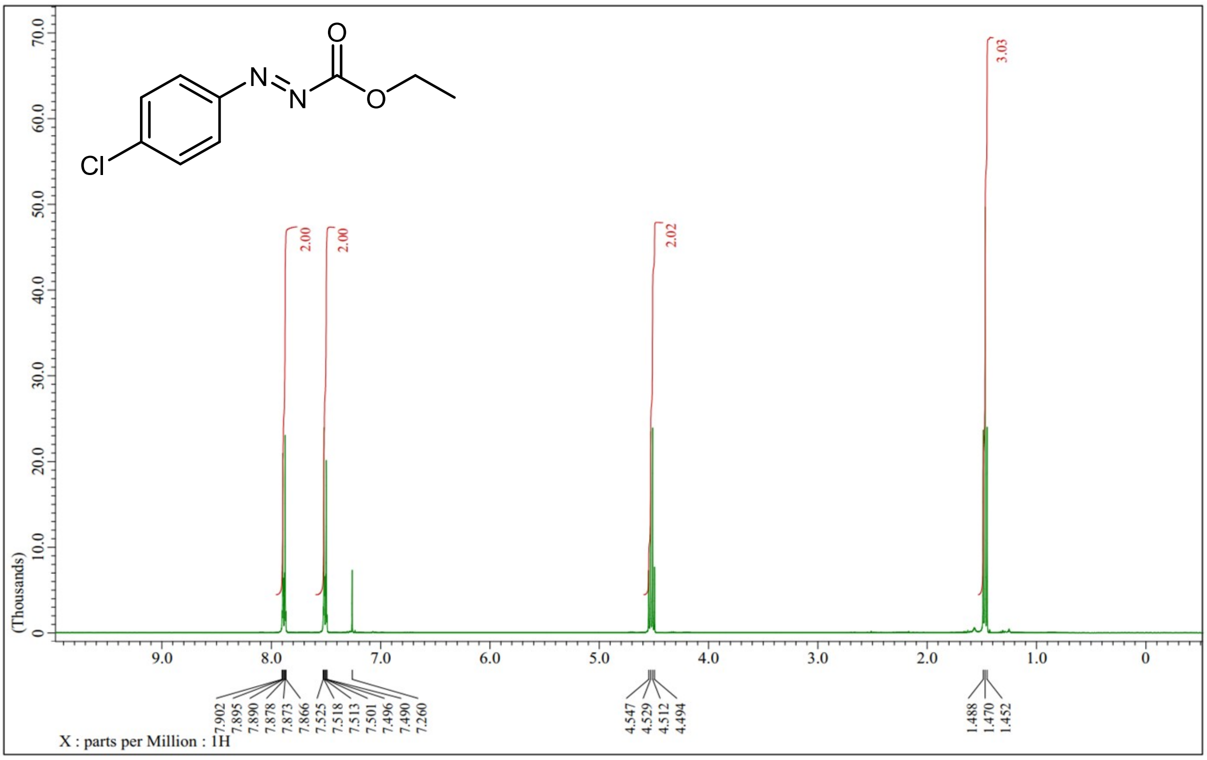

^{1}H NMR spectrum of ethyl (E)-2-(4-chlorophenyl)diazene-1-carboxylate (**2e**)

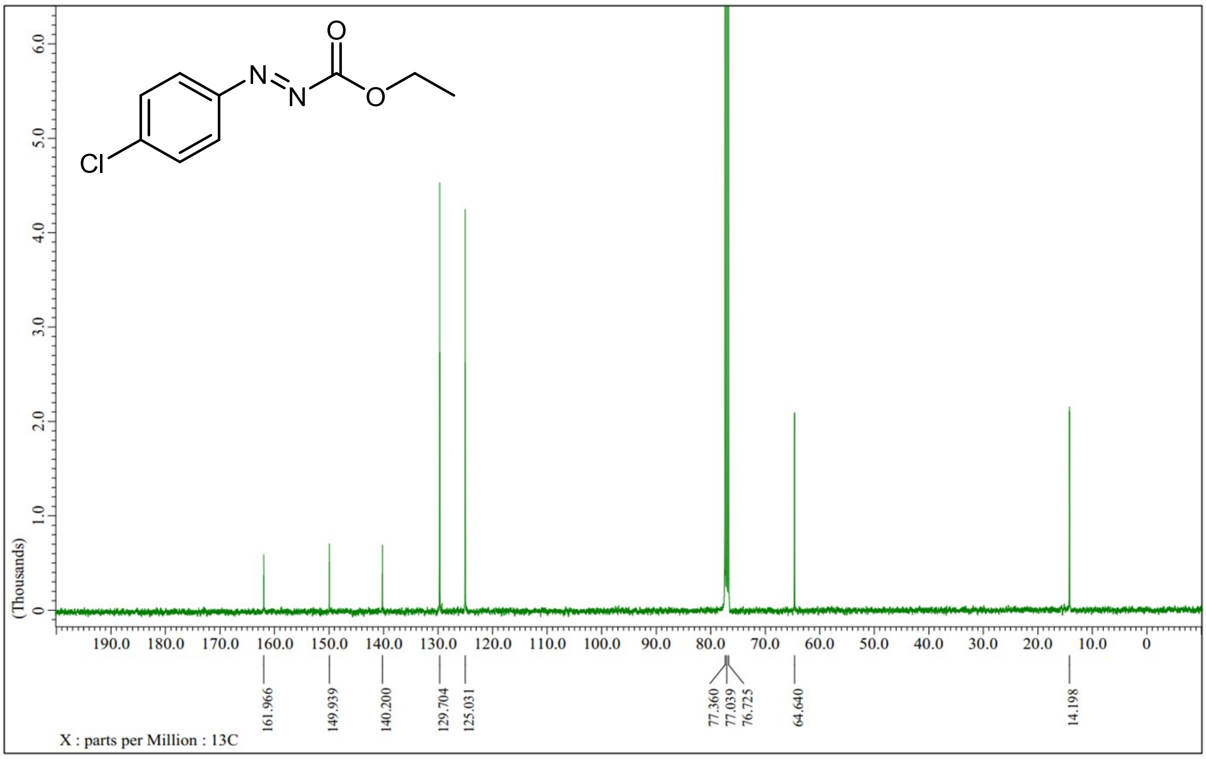

^{13}C NMR spectrum of ethyl (E)-2-(4-chlorophenyl)diazene-1-carboxylate (**2e**)

Ethyl (E)-2-(4-bromophenyl)diazene-1-carboxylate (2f)

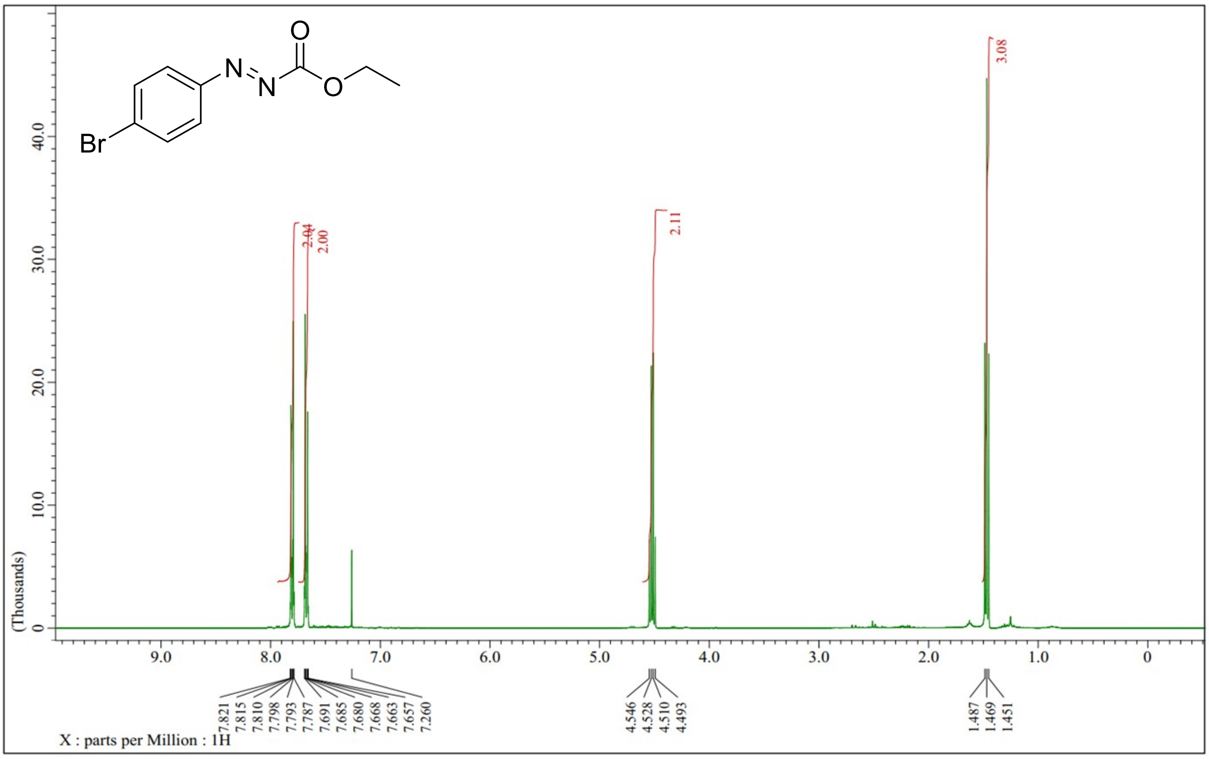

^{1}H NMR spectrum of ethyl (E)-2-(4-bromophenyl)diazene-1-carboxylate (**2f**)

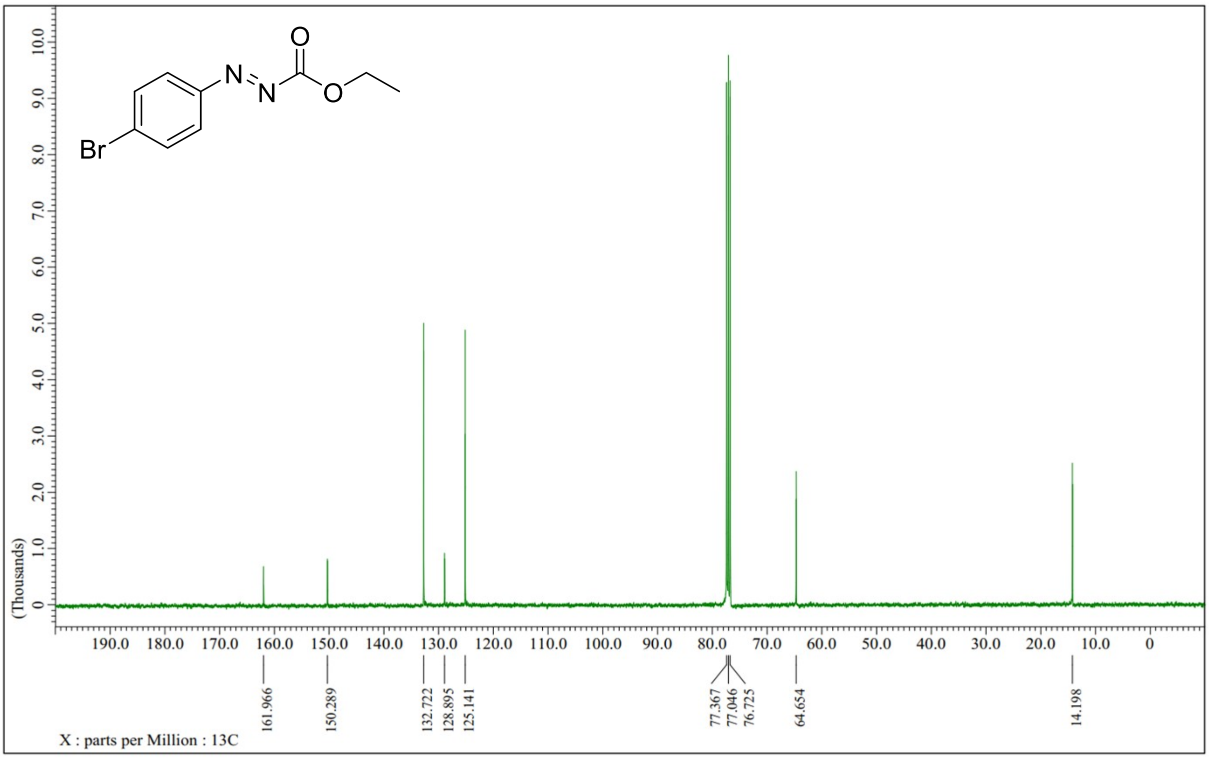

^{13}C NMR spectrum of ethyl (E)-2-(4-bromophenyl)diazene-1-carboxylate (**2f**)

Ethyl (E)-2-(4-nitrophenyl)diazene-1-carboxylate (2g)

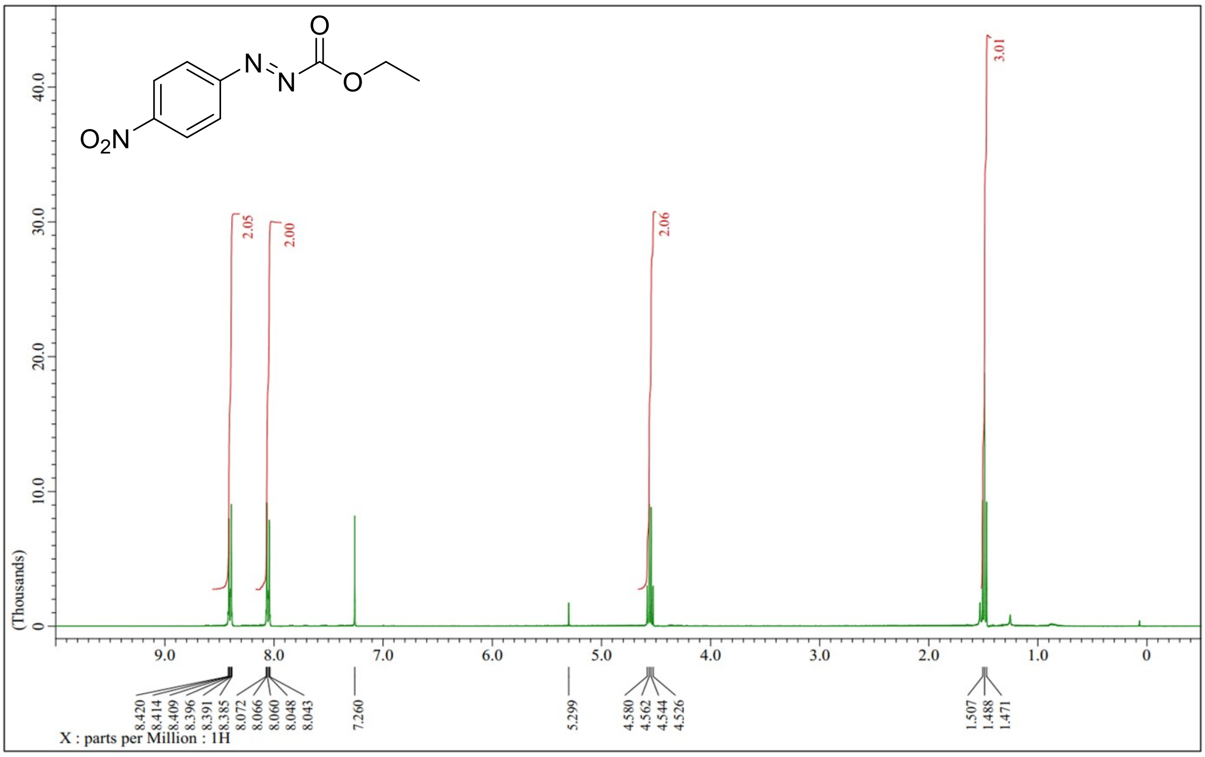

^1H NMR spectrum of ethyl (E)-2-(4-nitrophenyl)diazene-1-carboxylate (**2g**)

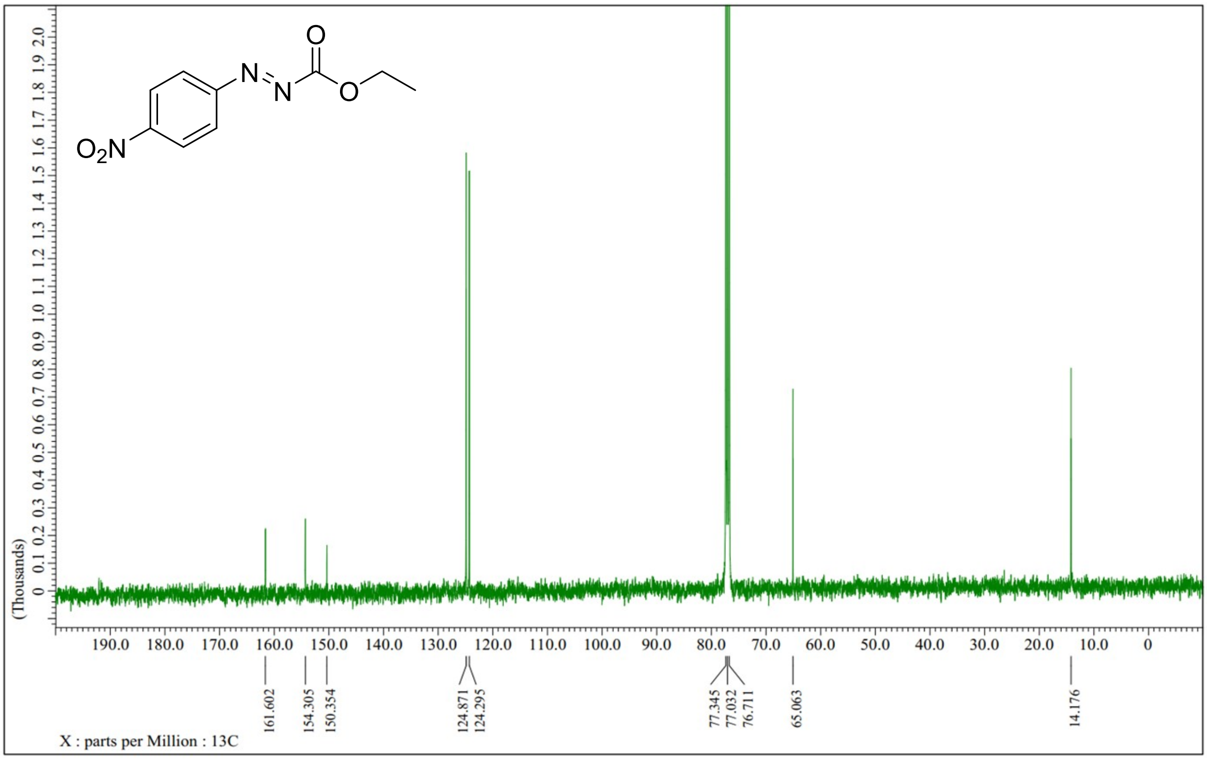

^{13}C NMR spectrum of ethyl (E)-2-(4-nitrophenyl)diazene-1-carboxylate (**2g**)

Ethyl (E)-2-(o-tolyl)diazene-1-carboxylate (2h)

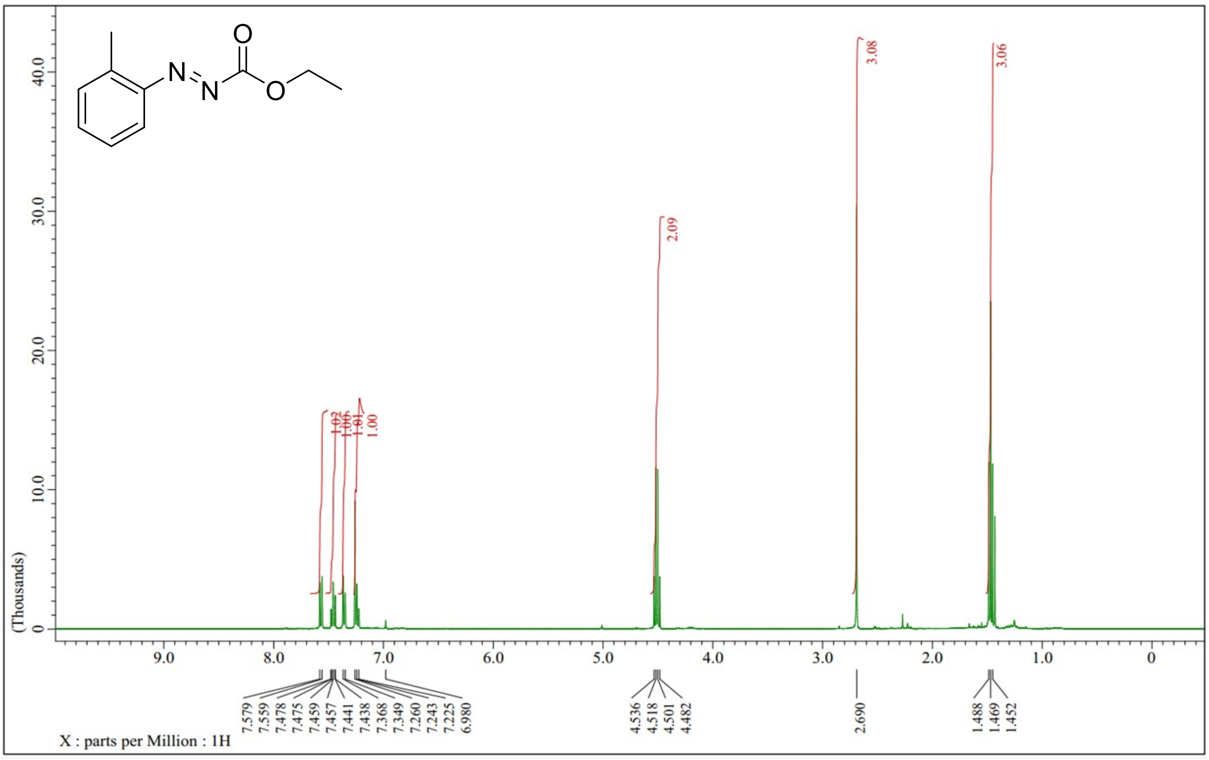

^{1}H NMR spectrum of ethyl (E)-2-(o-tolyl)diazene-1-carboxylate (**2h**)

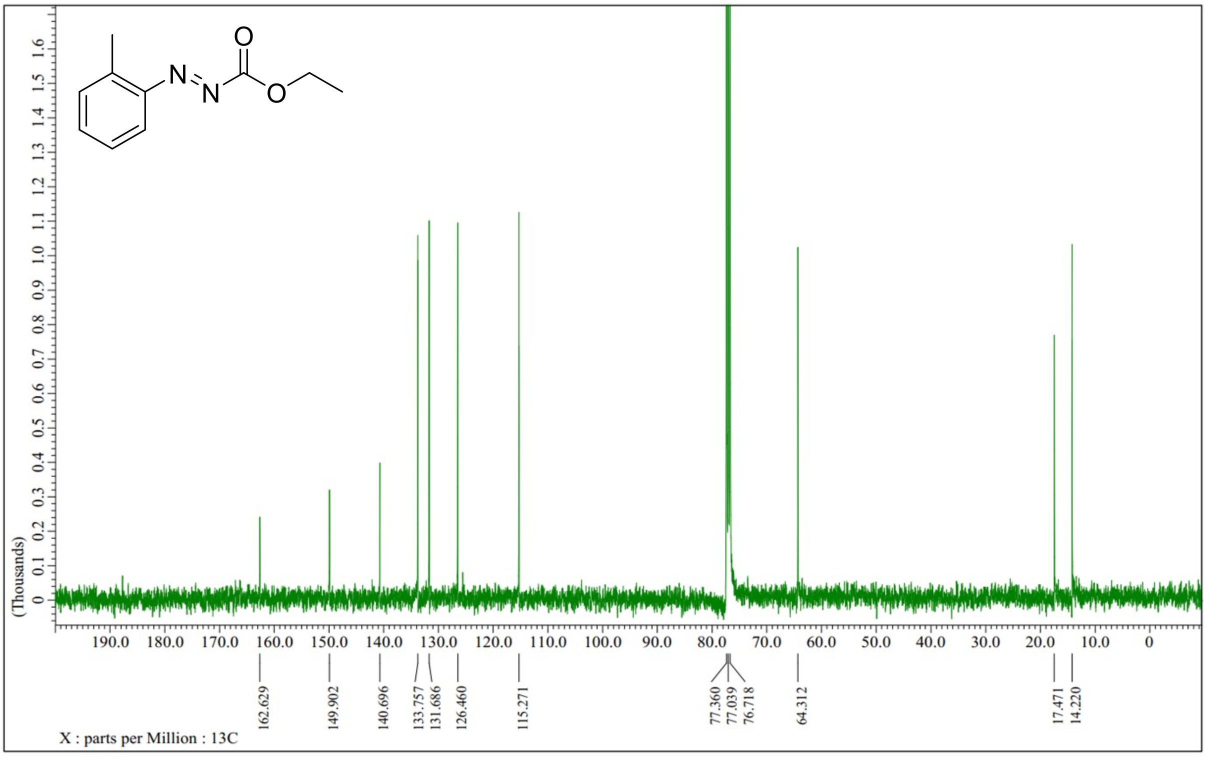

^{13}C NMR spectrum of ethyl (E)-2-(o-tolyl)diazene-1-carboxylate (**2h**)

Ethyl (E)-2-(3-chlorophenyl)diazene-1-carboxylate (2i)

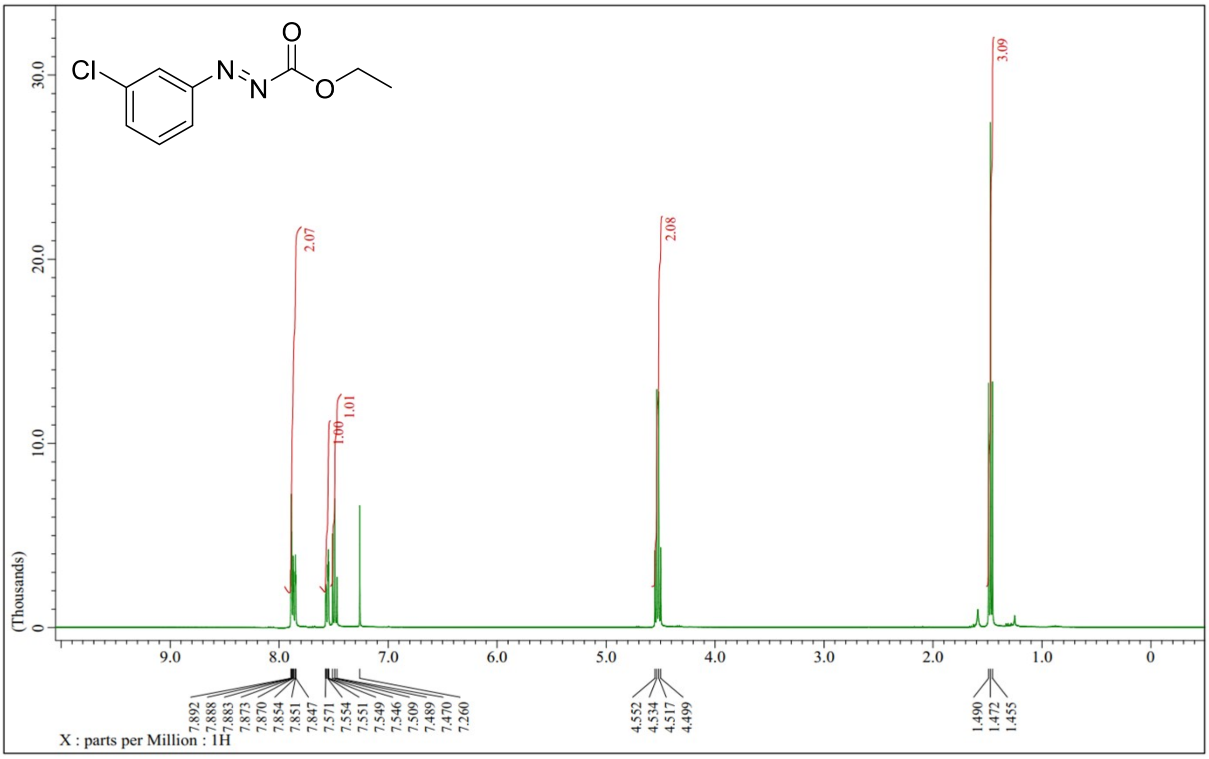

^{1}H NMR spectrum of ethyl (E)-2-(3-chlorophenyl)diazene-1-carboxylate (**2i**)

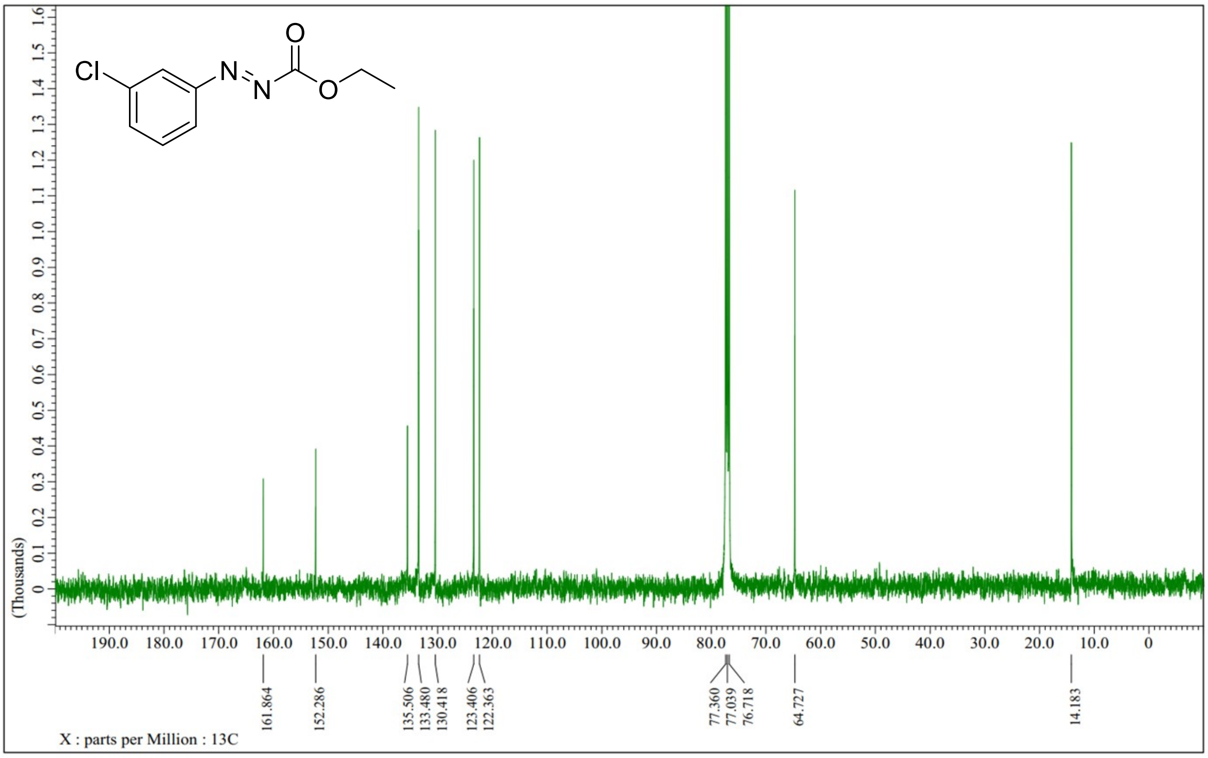

^{13}C NMR spectrum of ethyl (E)-2-(3-chlorophenyl)diazene-1-carboxylate (**2i**)

Ethyl (E)-2-(3,5-dimethylphenyl)diazene-1-carboxylate (2j)

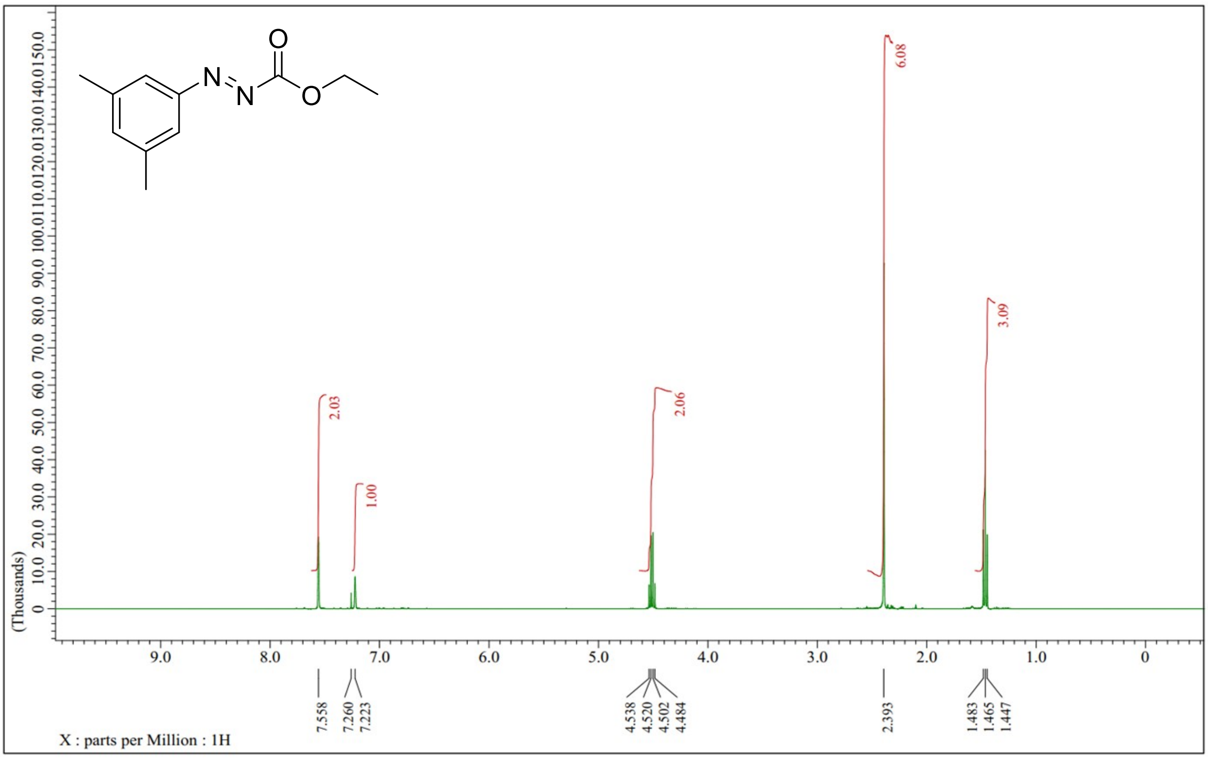

^{1}H NMR spectrum of ethyl (E)-2-(3,5-dimethylphenyl)diazene-1-carboxylate (**2j**)

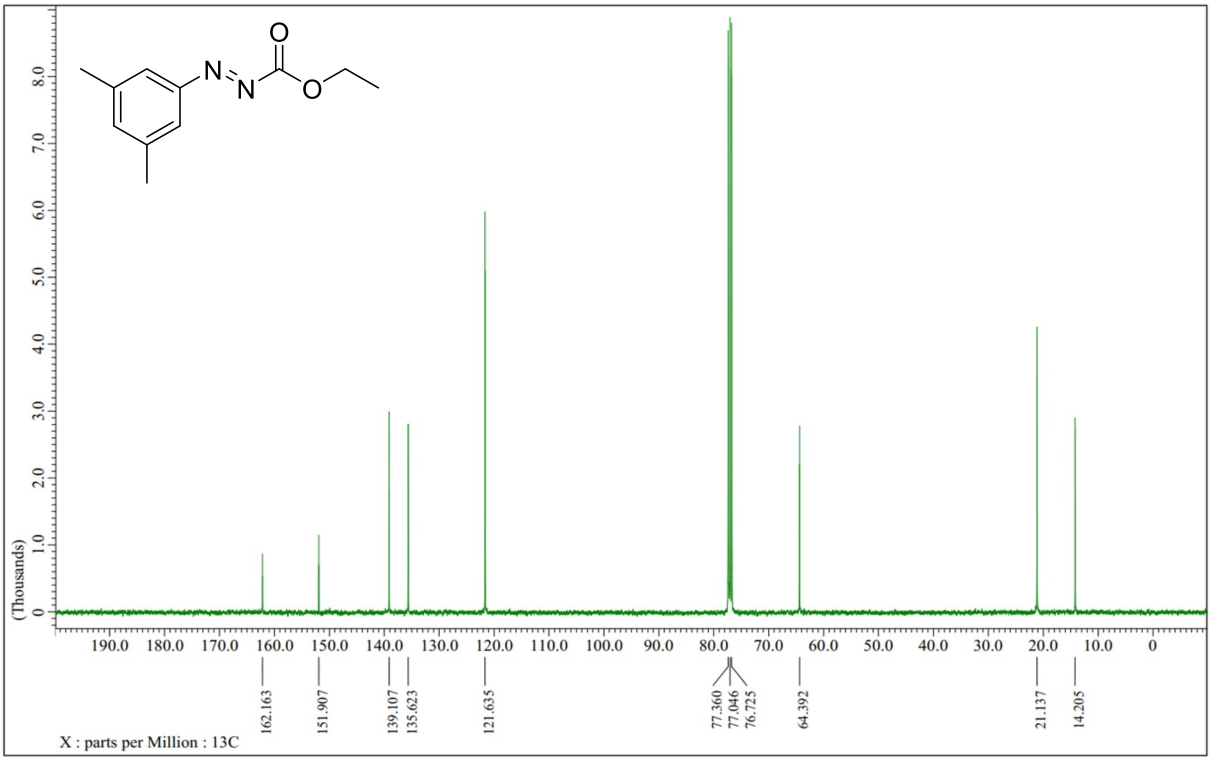

^{13}C NMR spectrum of ethyl (E)-2-(3,5-dimethylphenyl)diazene-1-carboxylate (**2j**)

Ethyl (*E*)-2-(3,5-dichlorophenyl)diazene-1-carboxylate (2k)

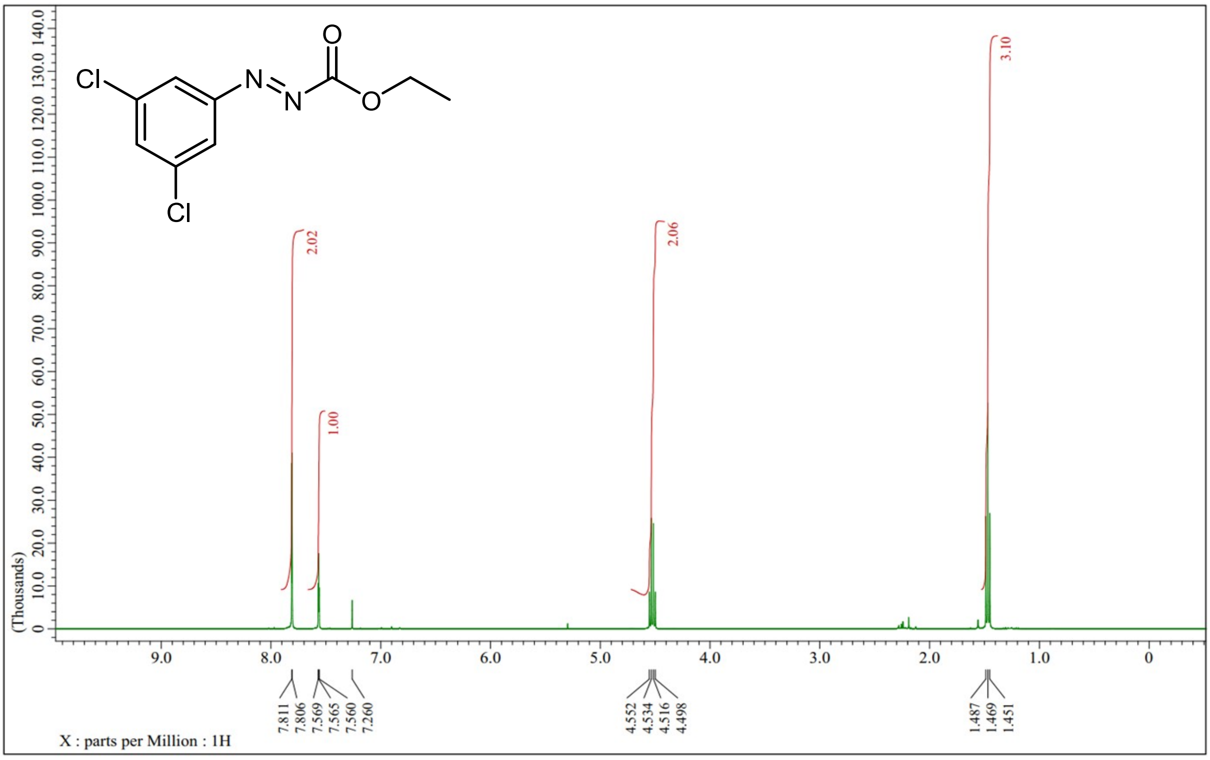

^{1}H NMR spectrum of ethyl (E)-2-(3,5-dichlorophenyl)diazene-1-carboxylate (**2k**)

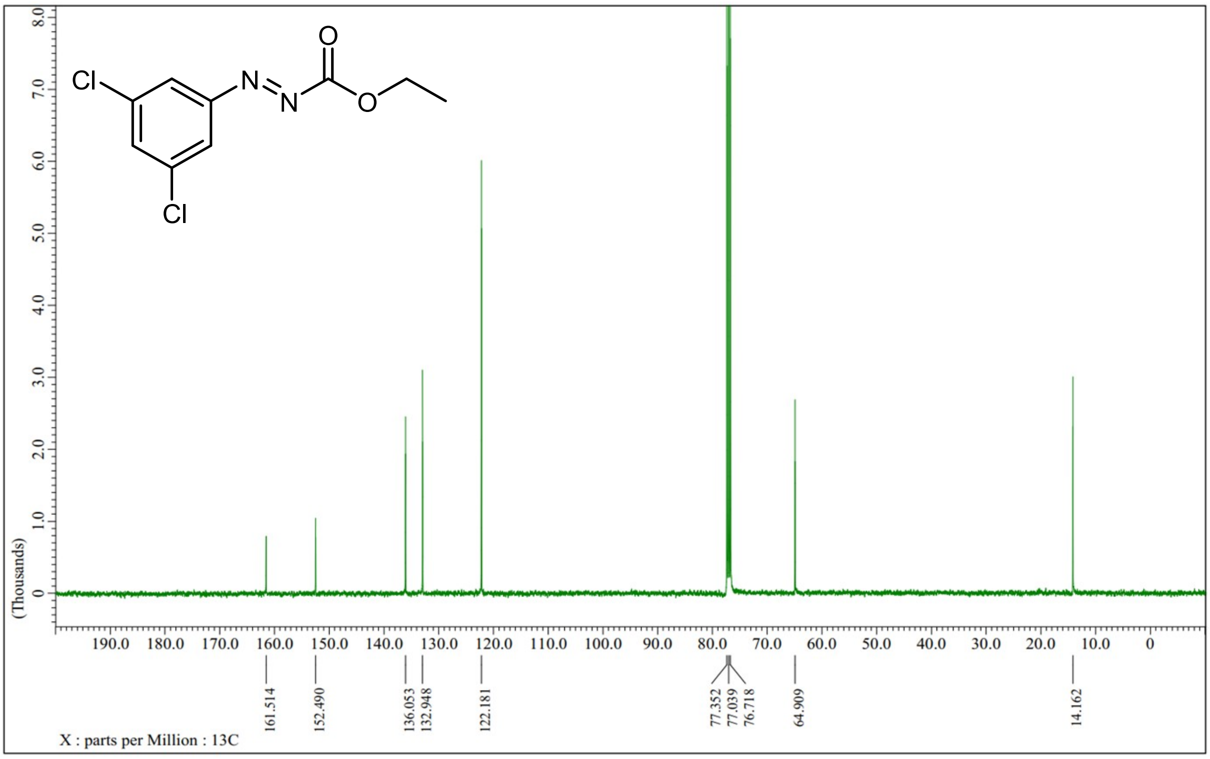

^{13}C NMR spectrum of ethyl (E)-2-(3,5-dichlorophenyl)diazene-1-carboxylate (**2k**)

Ethyl (E)-2-(benzo[d][1,3]dioxol-5-yl)diazene-1-carboxylate (2l)

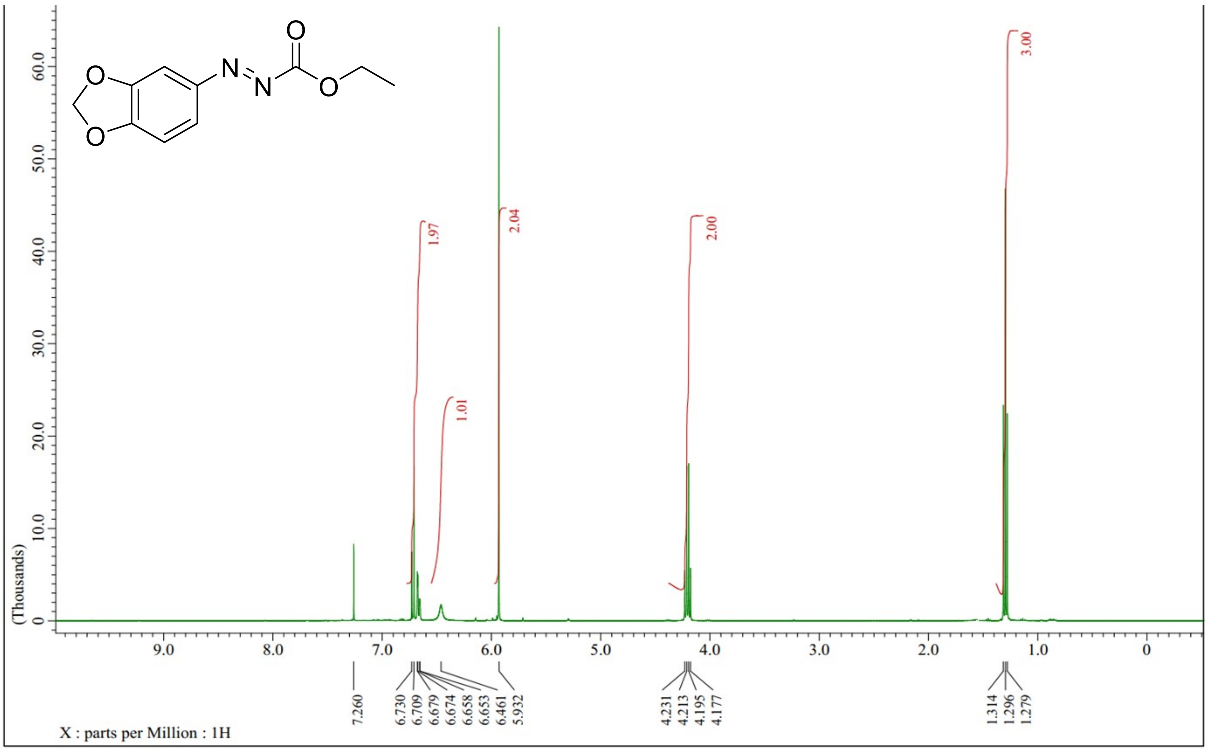

^{1}H NMR spectrum of ethyl (E)-2-(benzo[d][1,3]dioxol-5-yl)diazene-1-carboxylate (**2l**)

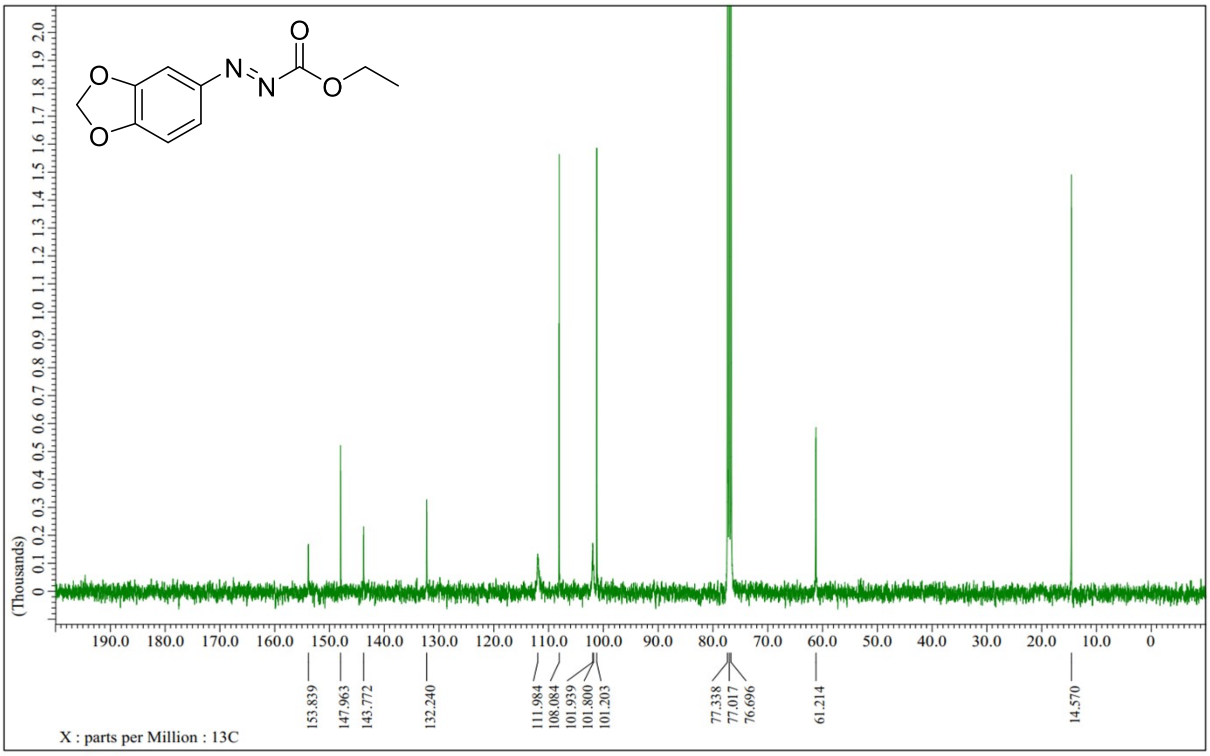

^{13}C NMR spectrum of ethyl (E)-2-(benzo[d][1,3]dioxol-5-yl)diazene-1-carboxylate (**2l**)

Ethyl (E)-2-(naphthalen-1-yl)diazene-1-carboxylate (2m)

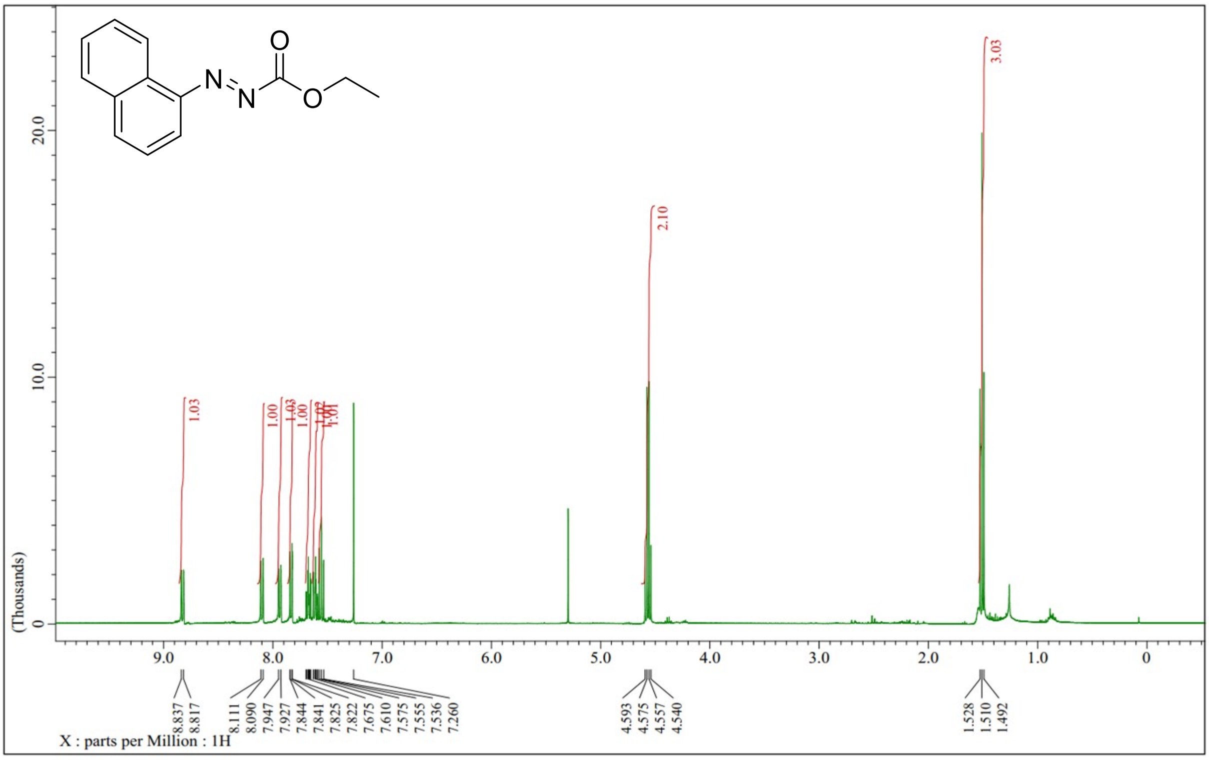

^{1}H NMR spectrum of ethyl (E)-2-(naphthalen-1-yl)diazene-1-carboxylate (**2m**)

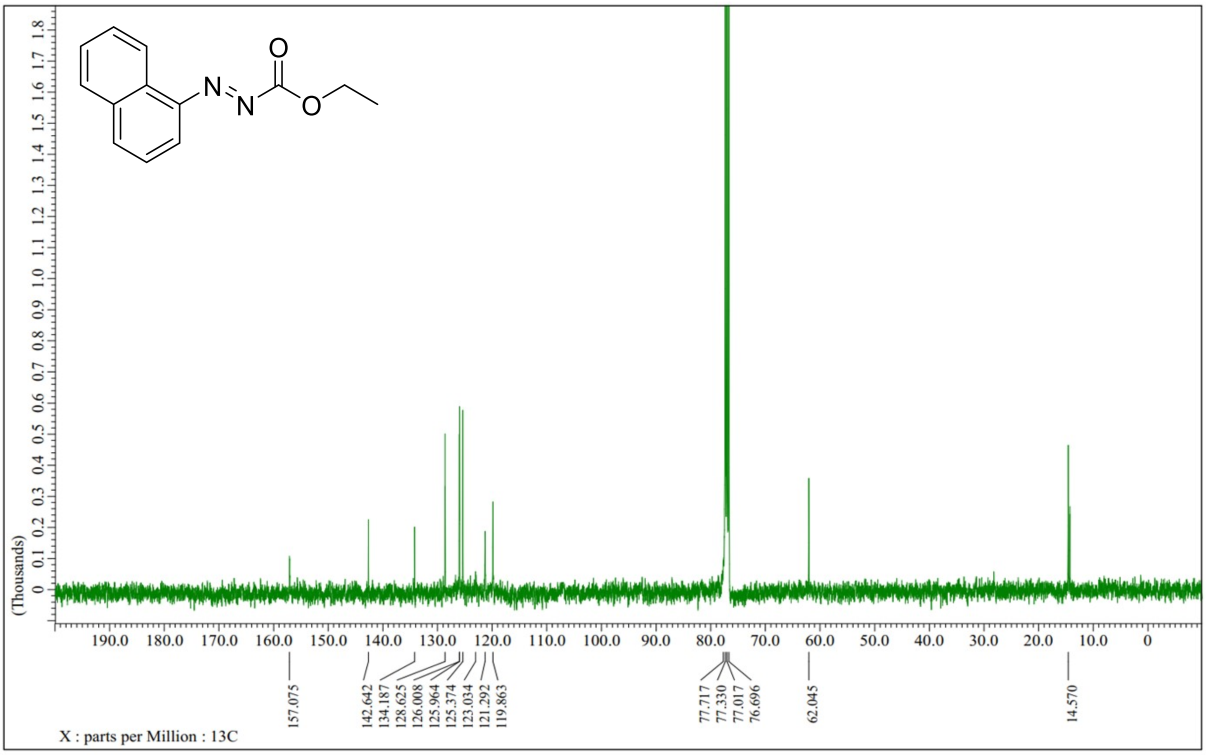

^{13}C NMR spectrum of ethyl (E)-2-(naphthalen-1-yl)diazene-1-carboxylate (**2m**)

Benzyl (E)-2-phenyldiazene-1-carboxylate (2n)

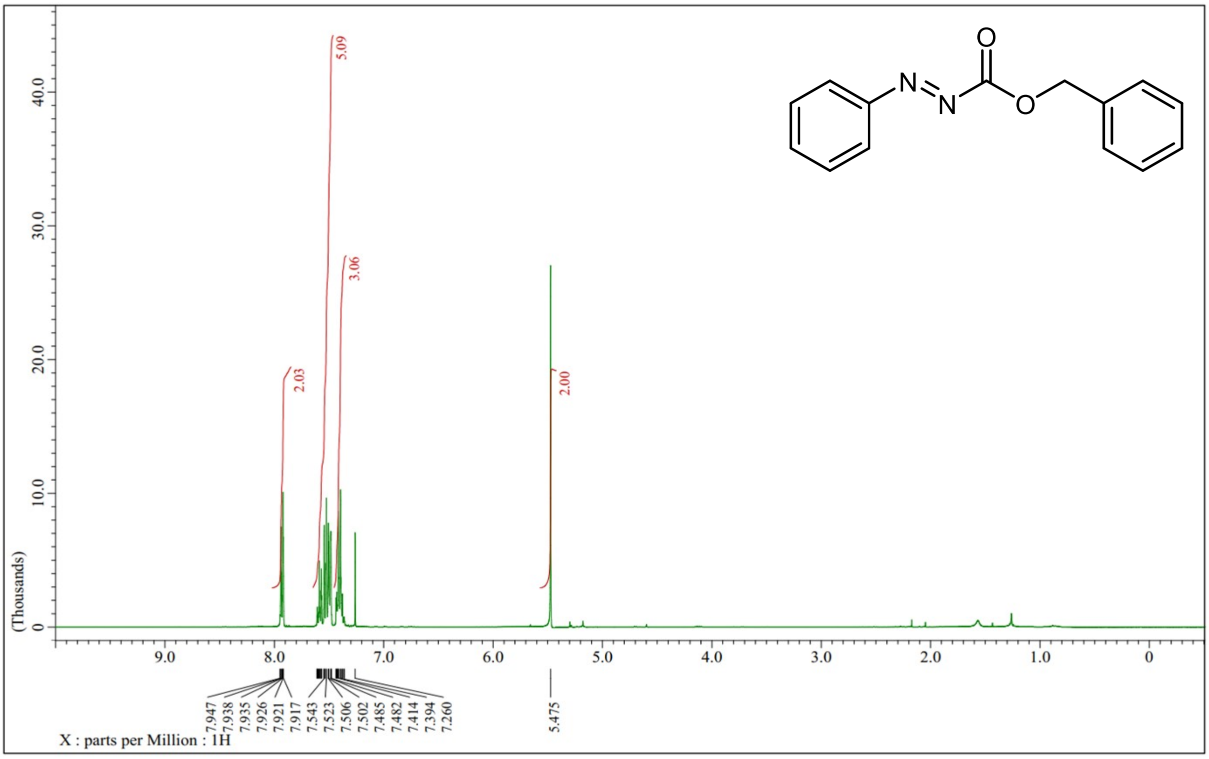

^{1}H NMR spectrum of benzyl (E)-2-phenyldiazene-1-carboxylate (**2n**)

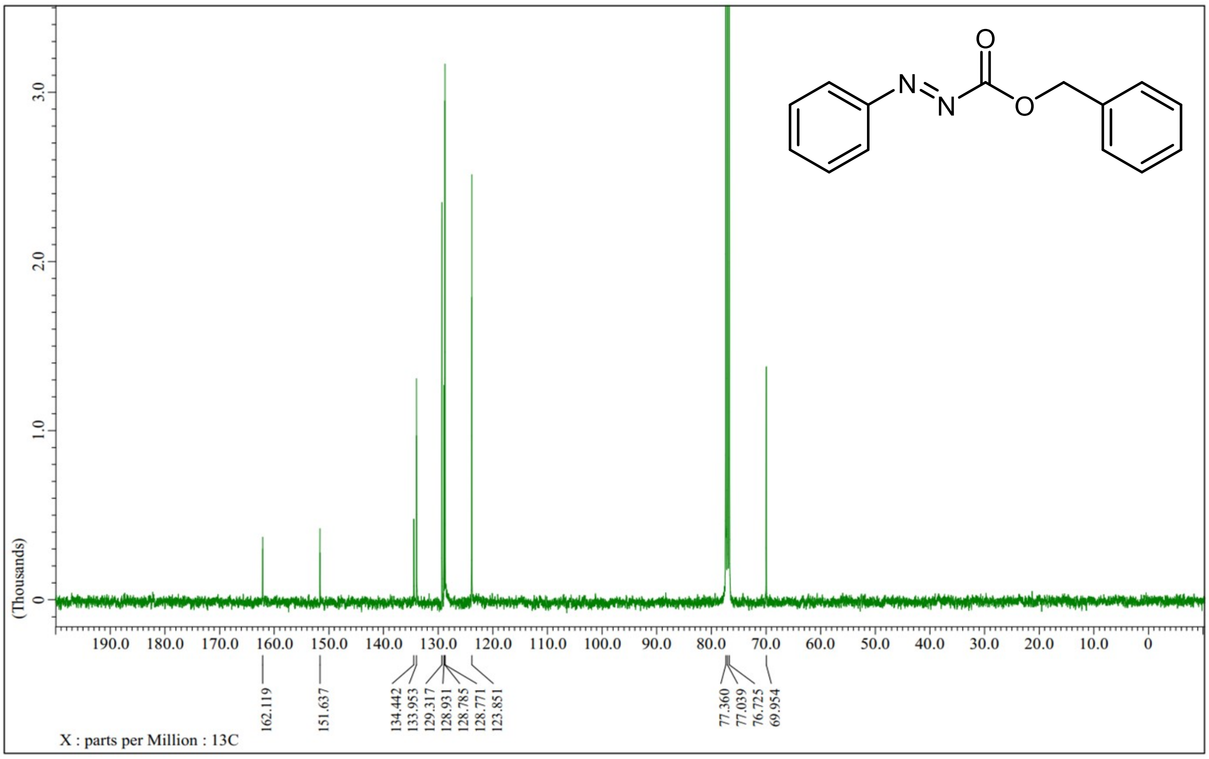

^{13}C NMR spectrum of benzyl (E)-2-phenyldiazene-1-carboxylate (**2n**)

Benzyl (E)-2-(*p*-tolyl)diazene-1-carboxylate (2o)

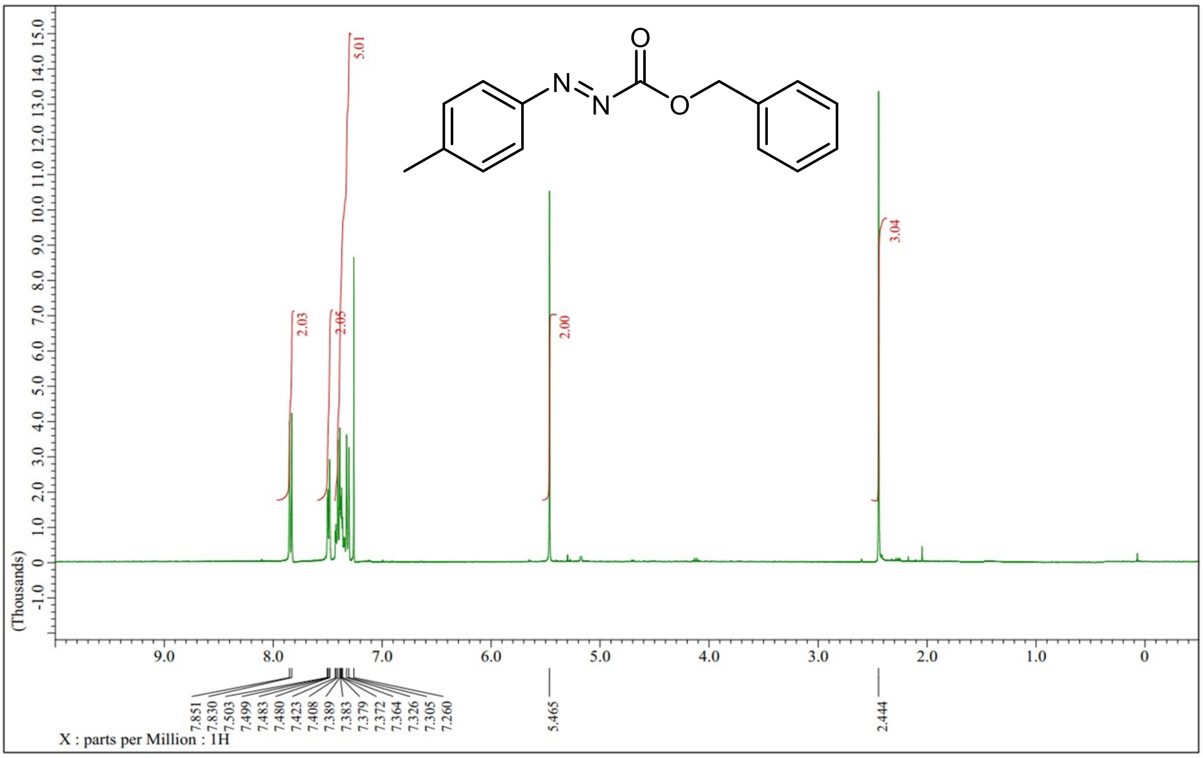

^{1}H NMR spectrum of benzyl (E)-2-(*p*-tolyl)diazene-1-carboxylate (**2o**)

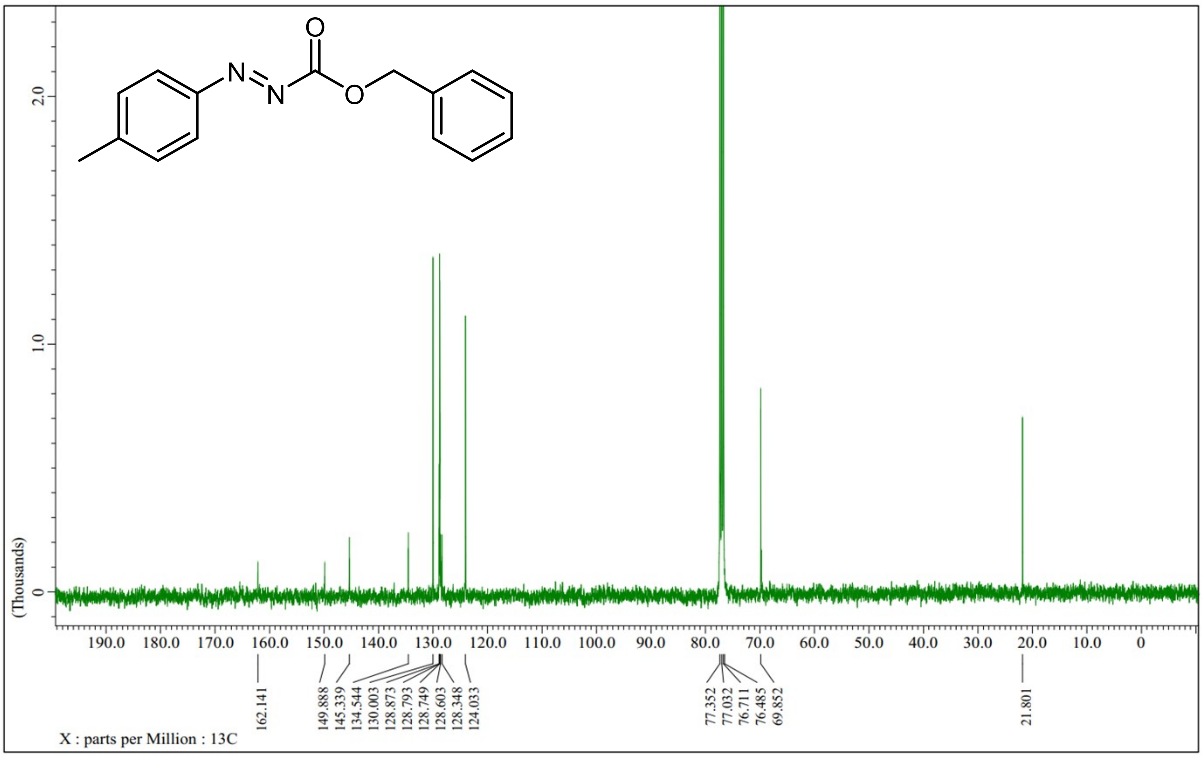

^{13}C NMR spectrum of benzyl (E)-2-(*p*-tolyl)diazene-1-carboxylate (**2o**)

Benzyl (*E*)-2-(4-chlorophenyl)diazene-1-carboxylate (2p)

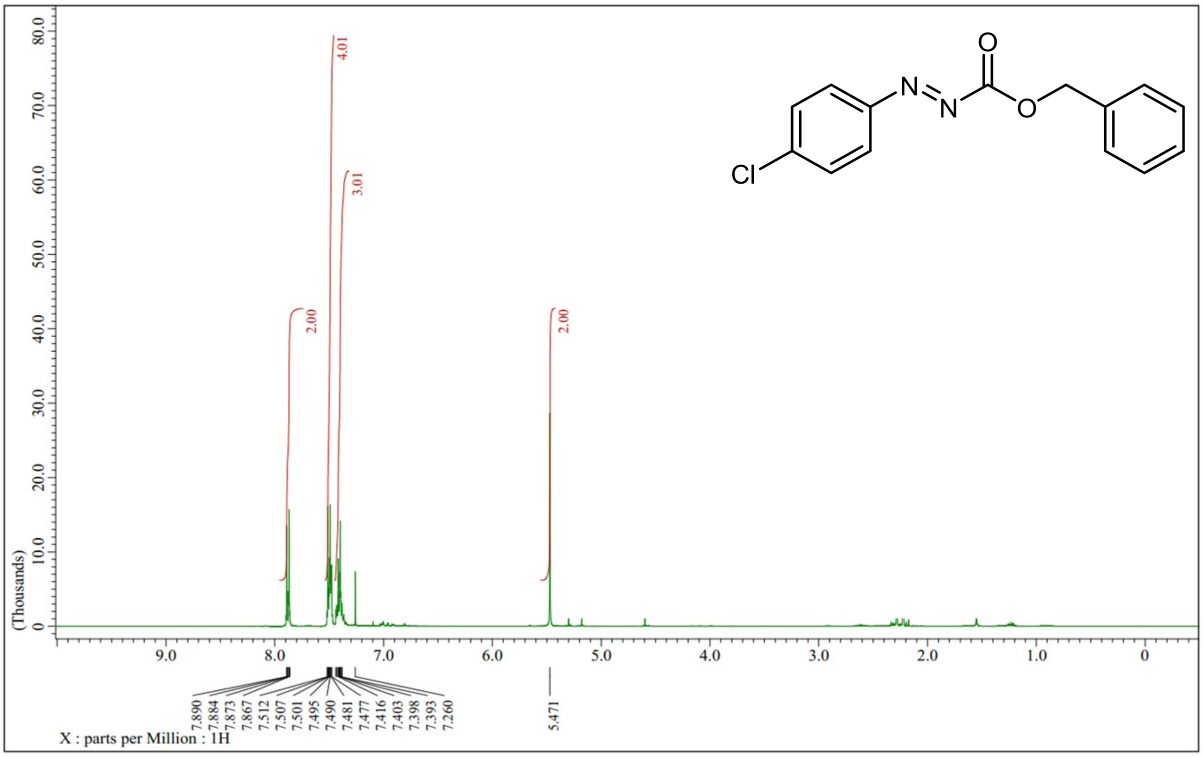

^{1}H NMR spectrum of benzyl (E)-2-(4-chlorophenyl)diazene-1-carboxylate (**2p**)

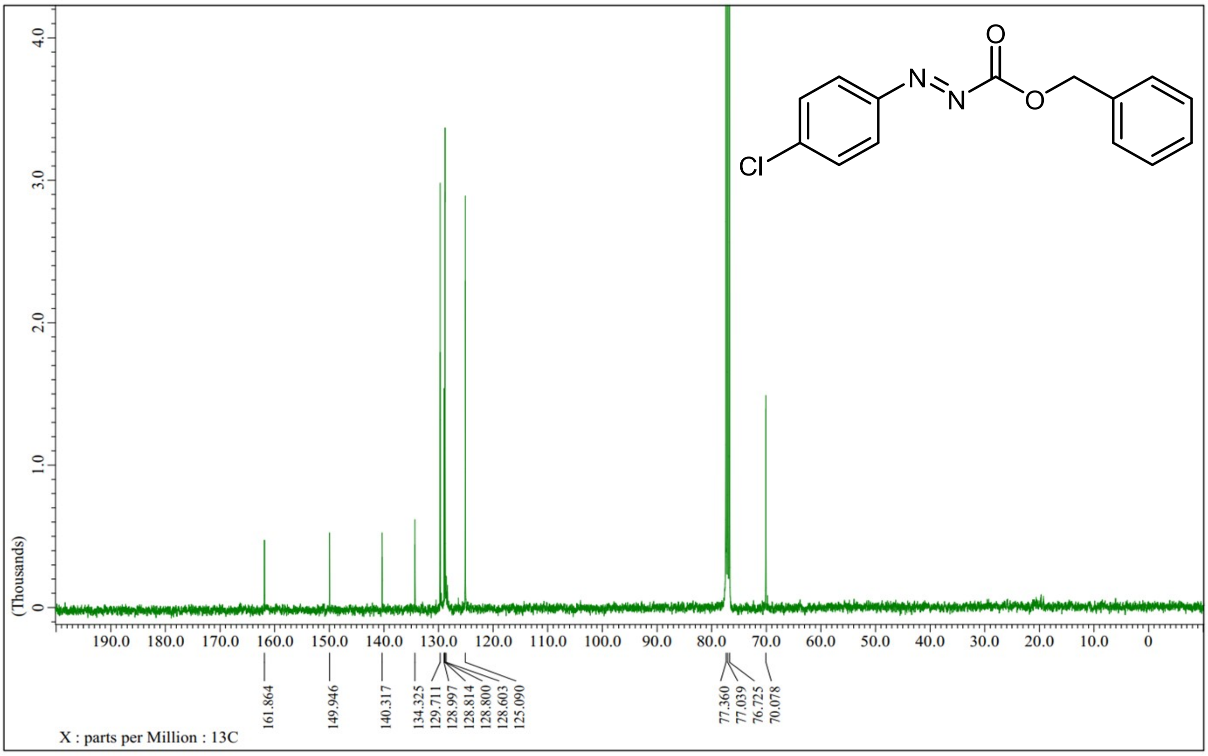

^{13}C NMR spectrum of benzyl (E)-2-(4-chlorophenyl)diazene-1-carboxylate (**2p**)

Benzyl (E)-2-(4-nitrophenyl)diazene-1-carboxylate (2q)

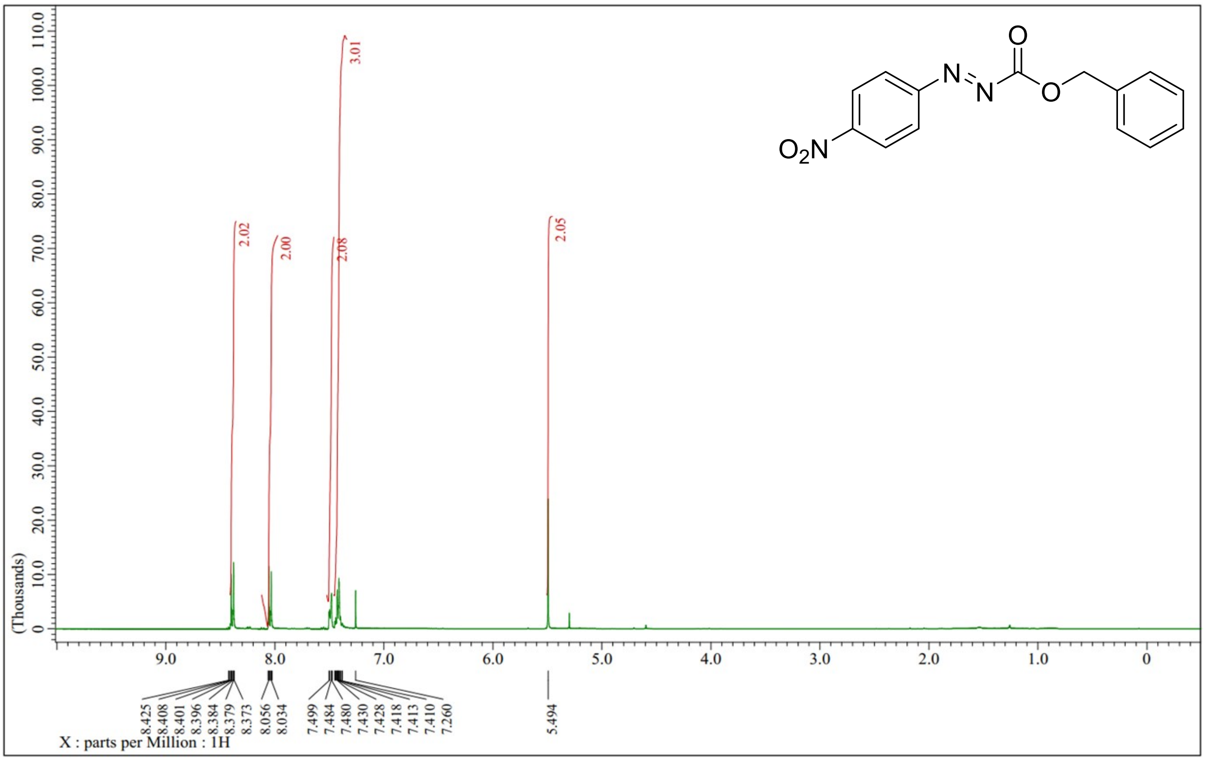

^{1}H NMR spectrum of benzyl (E)-2-(4-nitrophenyl)diazene-1-carboxylate (**2q**)

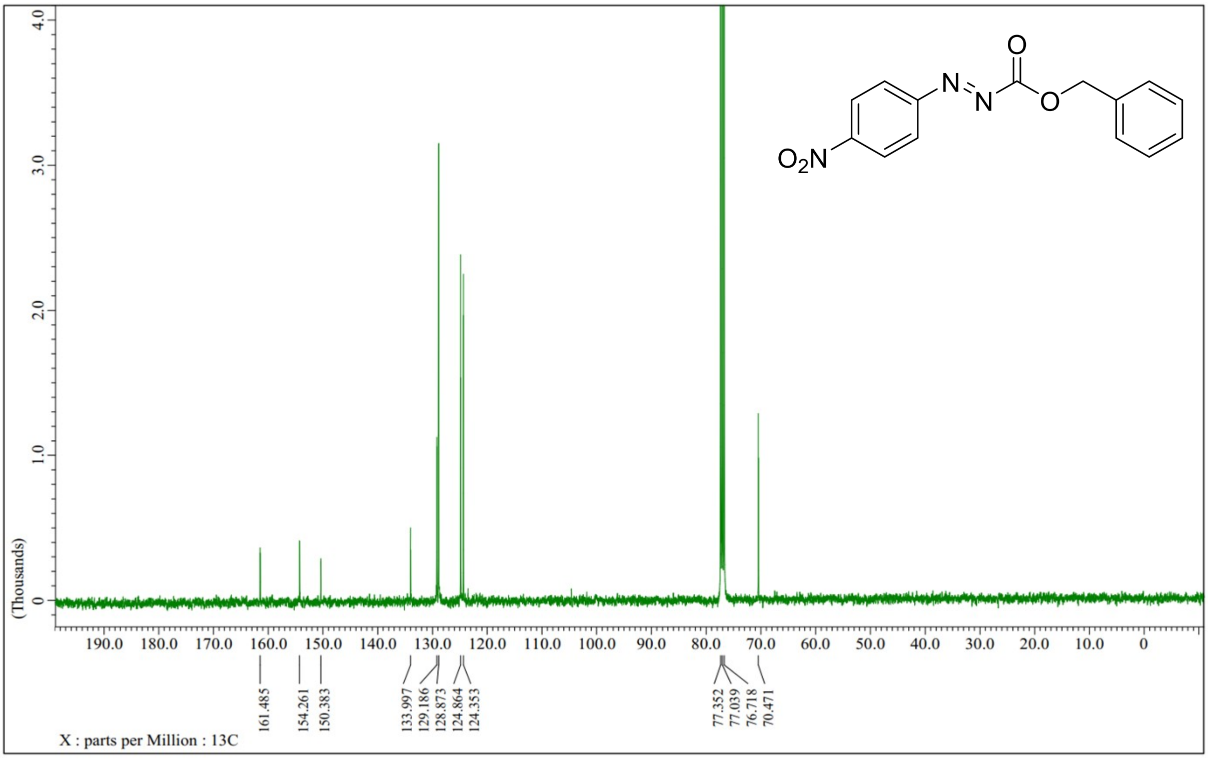

^{13}C NMR spectrum of benzyl (E)-2-(4-nitrophenyl)diazene-1-carboxylate (**2q**)

2,2,2-trichloroethyl (E)-2-phenyldiazene-1-carboxylate (2r)

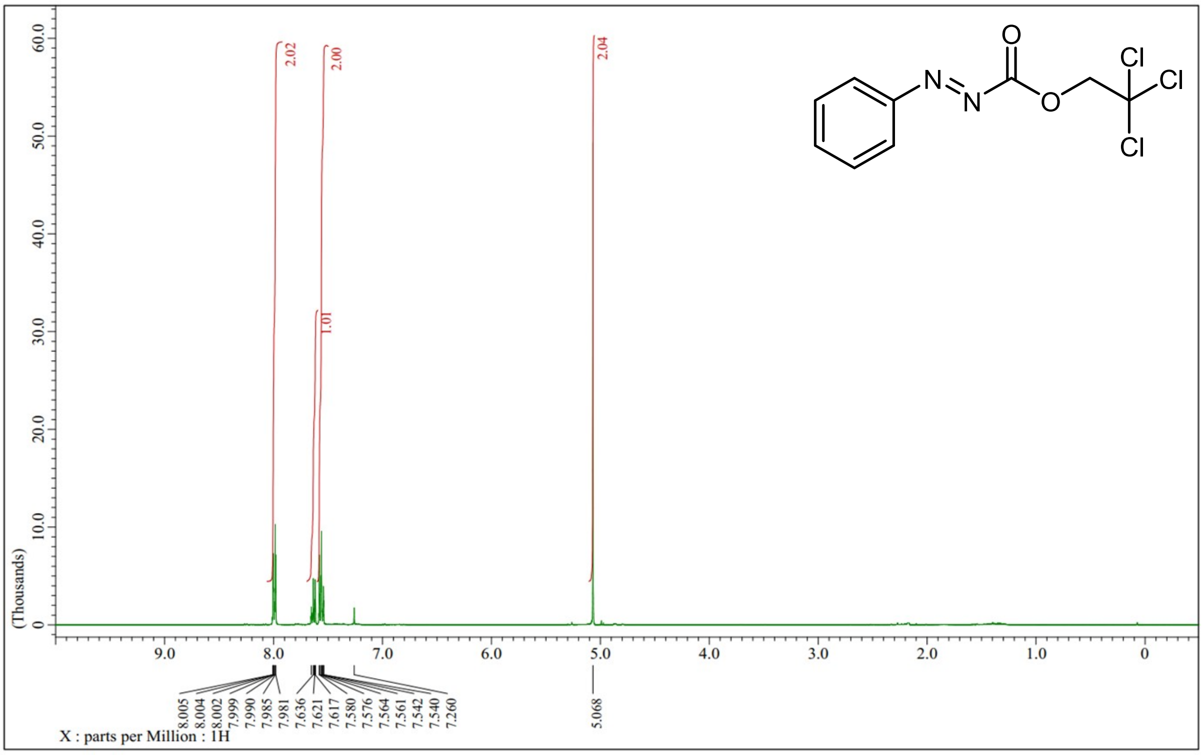

^{1}H NMR spectrum of 2,2,2-trichloroethyl (E)-2-phenyldiazene-1-carboxylate (**2r**)

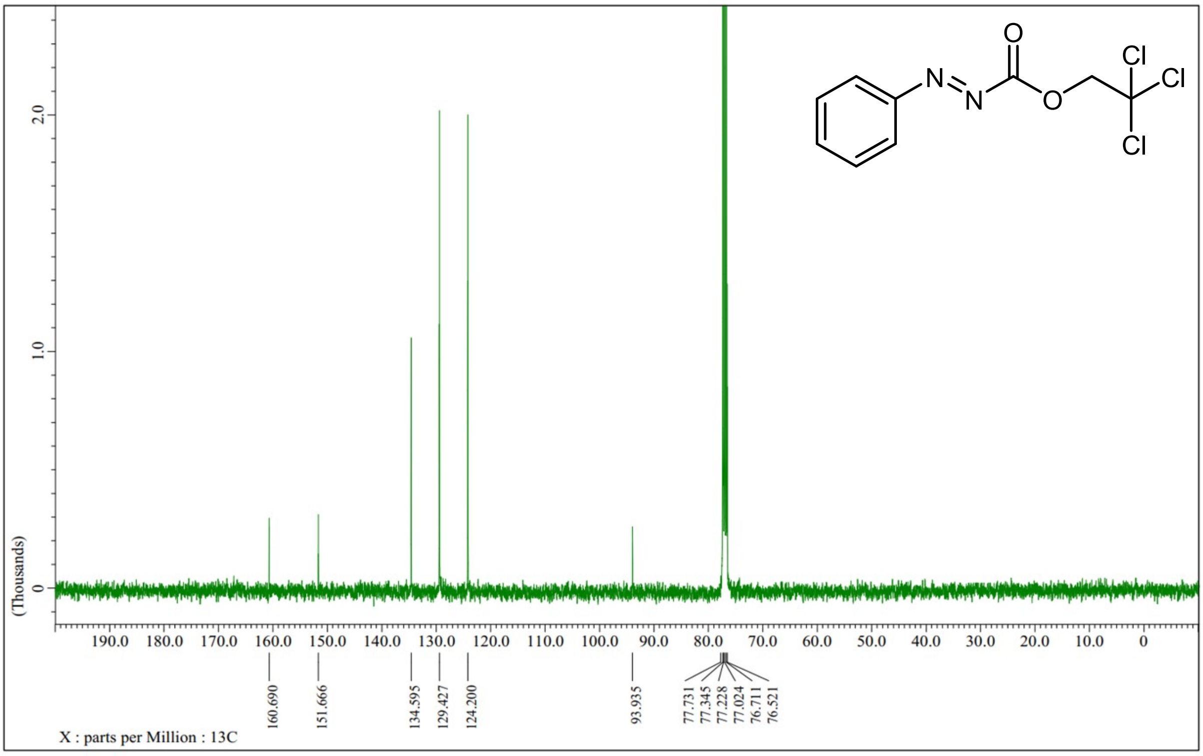

^{13}C NMR spectrum of 2,2,2-trichloroethyl (E)-2-phenyldiazene-1-carboxylate (**2r**)

2,2,2-trichloroethyl (E)-2-(p-tolyl)diazene-1-carboxylate (2s)

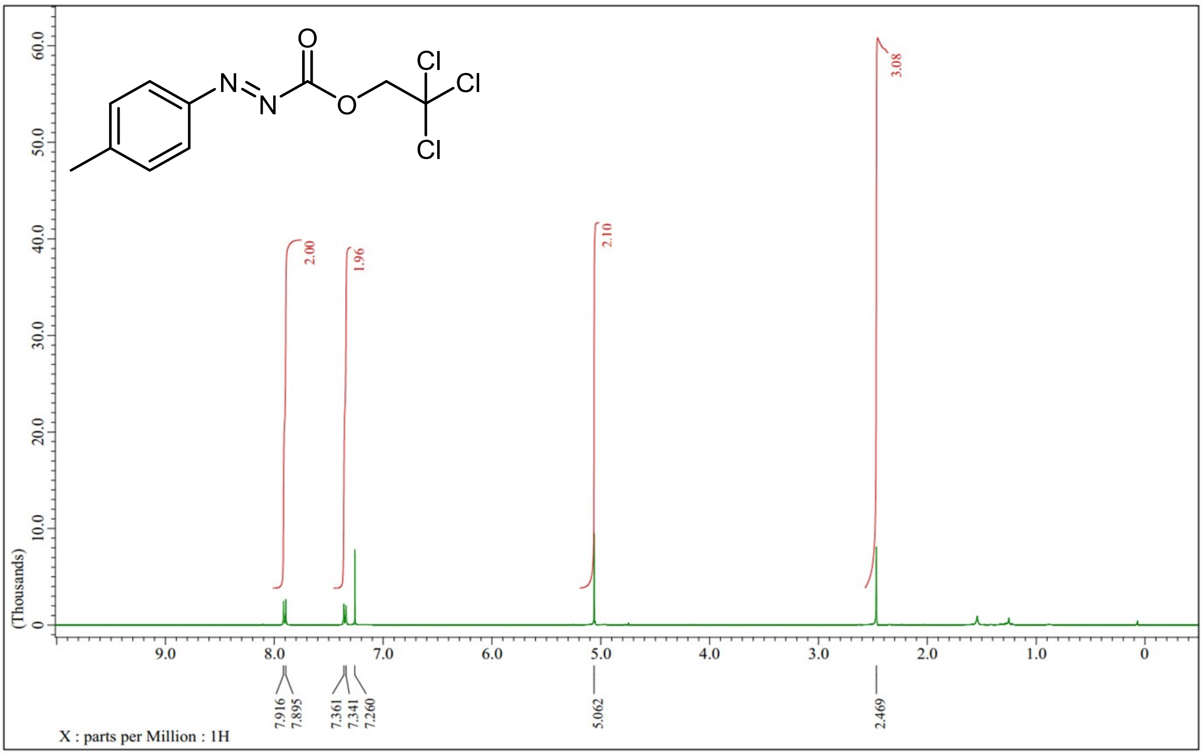

^{1}H NMR spectrum of 2,2,2-trichloroethyl (E)-2-(p-tolyl)diazene-1-carboxylate (**2s**)

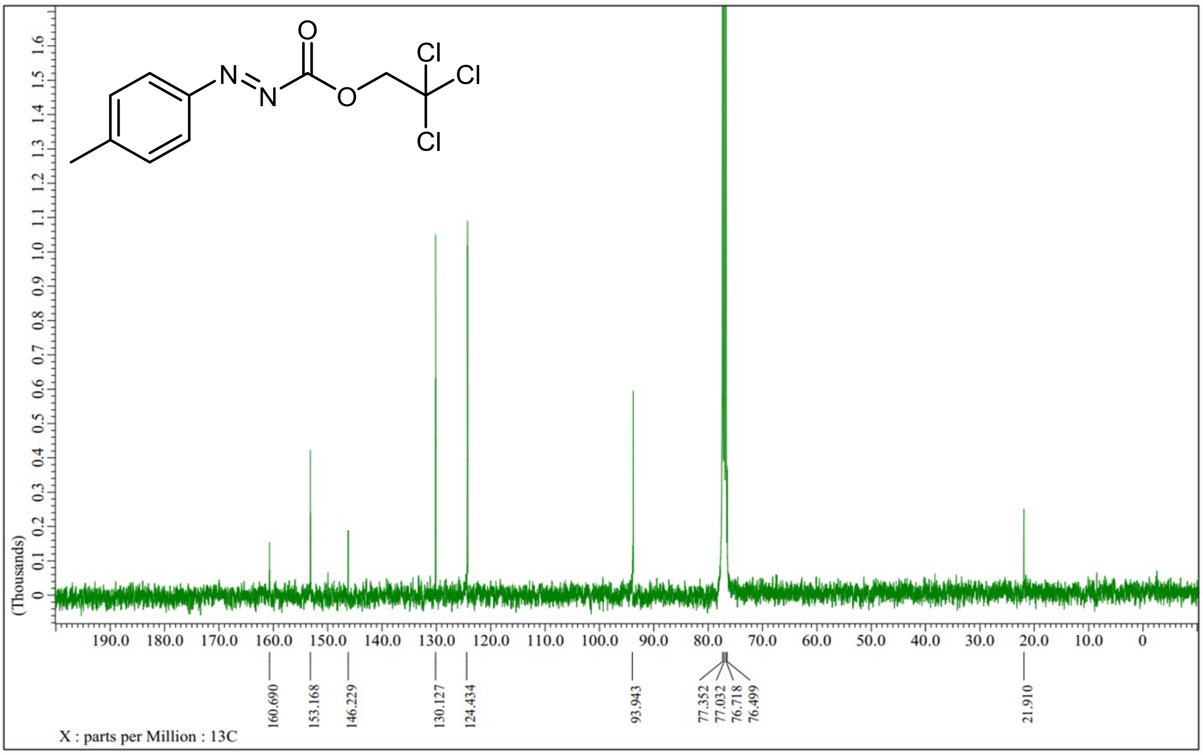

^{13}C NMR spectrum of 2,2,2-trichloroethyl (E)-2-(p-tolyl)diazene-1-carboxylate (**2s**)

2,2,2-trichloroethyl (E)-2-(naphthalen-1-yl)diazene-1-carboxylate (2t)

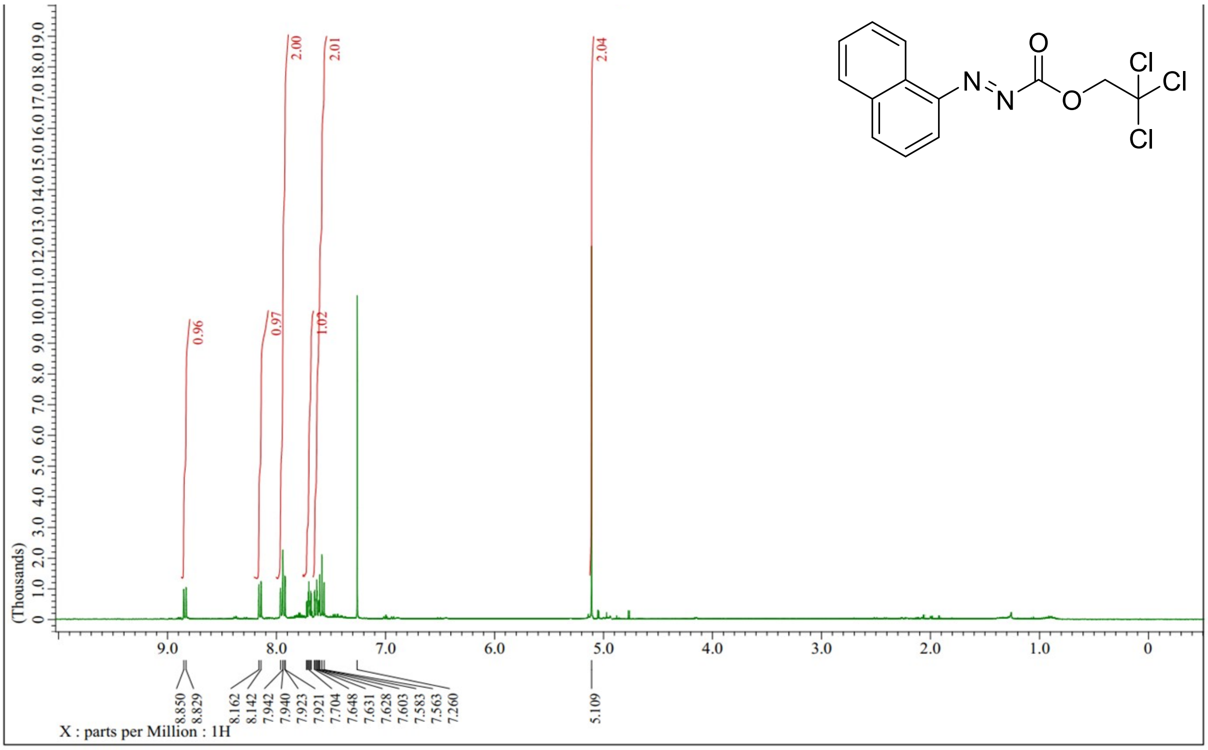

^{1}H NMR spectrum of 2,2,2-trichloroethyl (E)-2-(naphthalen-1-yl)diazene-1-carboxylate (**2t**)

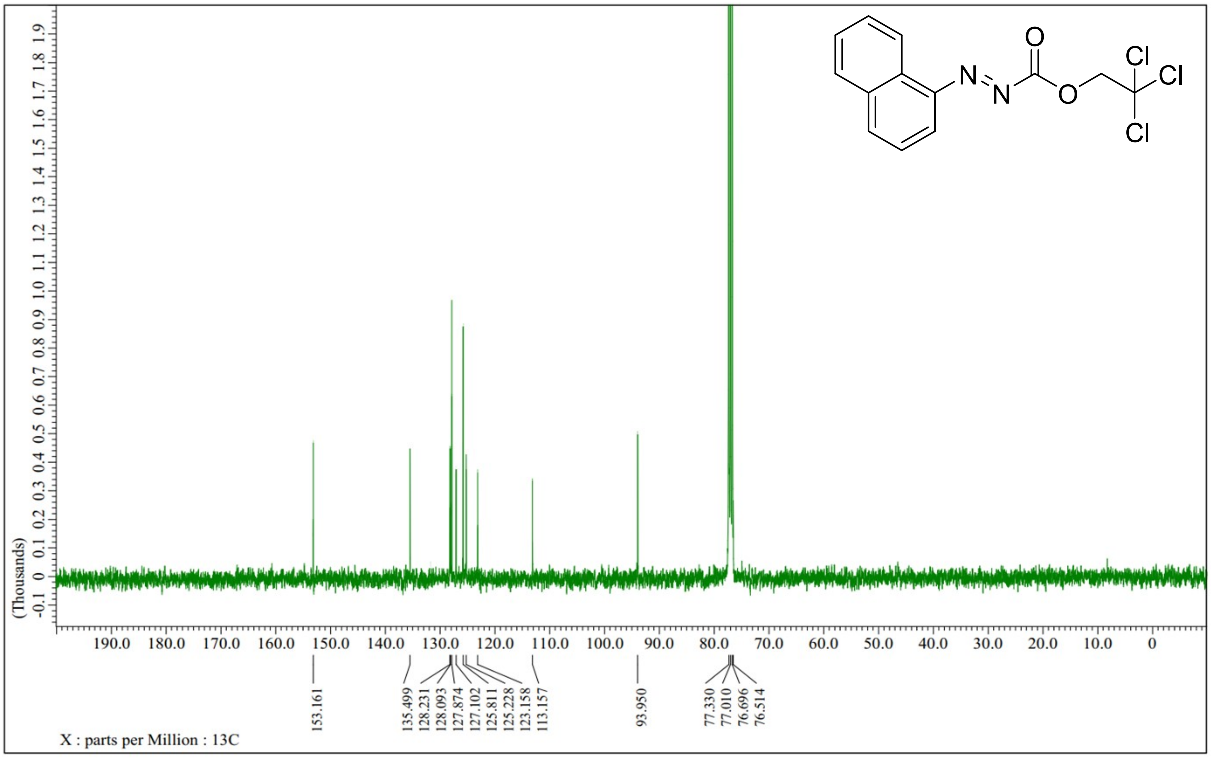

^{13}C NMR spectrum of 2,2,2-trichloroethyl (E)-2-(naphthalen-1-yl)diazene-1-carboxylate (**2t**)

***tert*-butyl (E)-2-phenyldiazene-1-carboxylate (2u)**

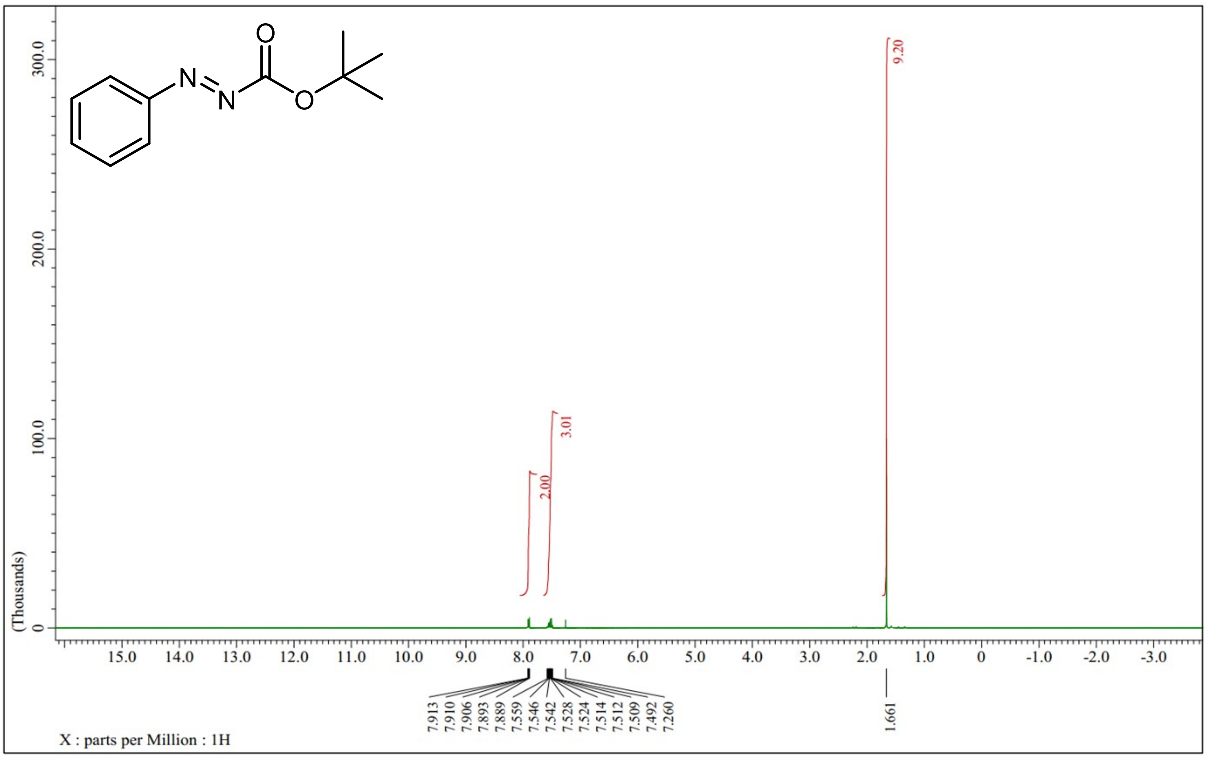

^{1}H NMR spectrum of *tert*-butyl (E)-2-phenyldiazene-1-carboxylate (**2u**)

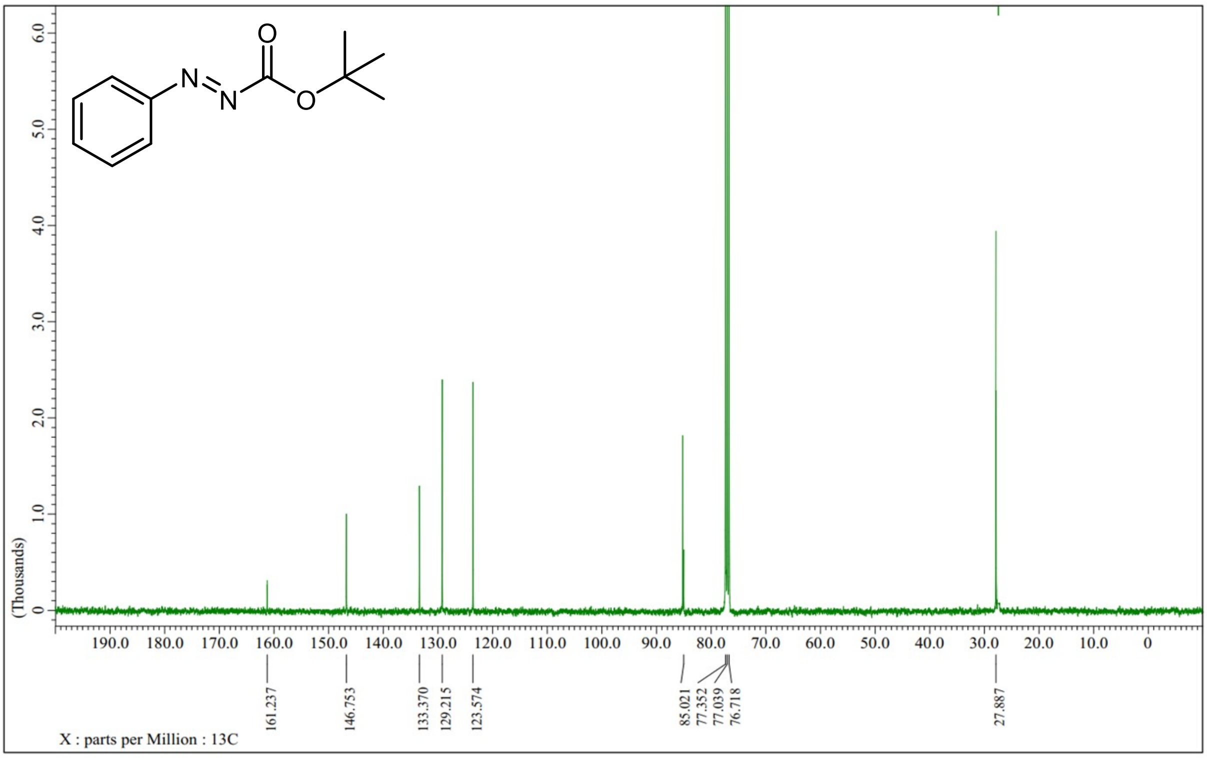

^{13}C NMR spectrum of *tert*-butyl (E)-2-phenyldiazene-1-carboxylate (**2u**)

tert-butyl (E)-2-(p-tolyl)diazene-1-carboxylate (2v)

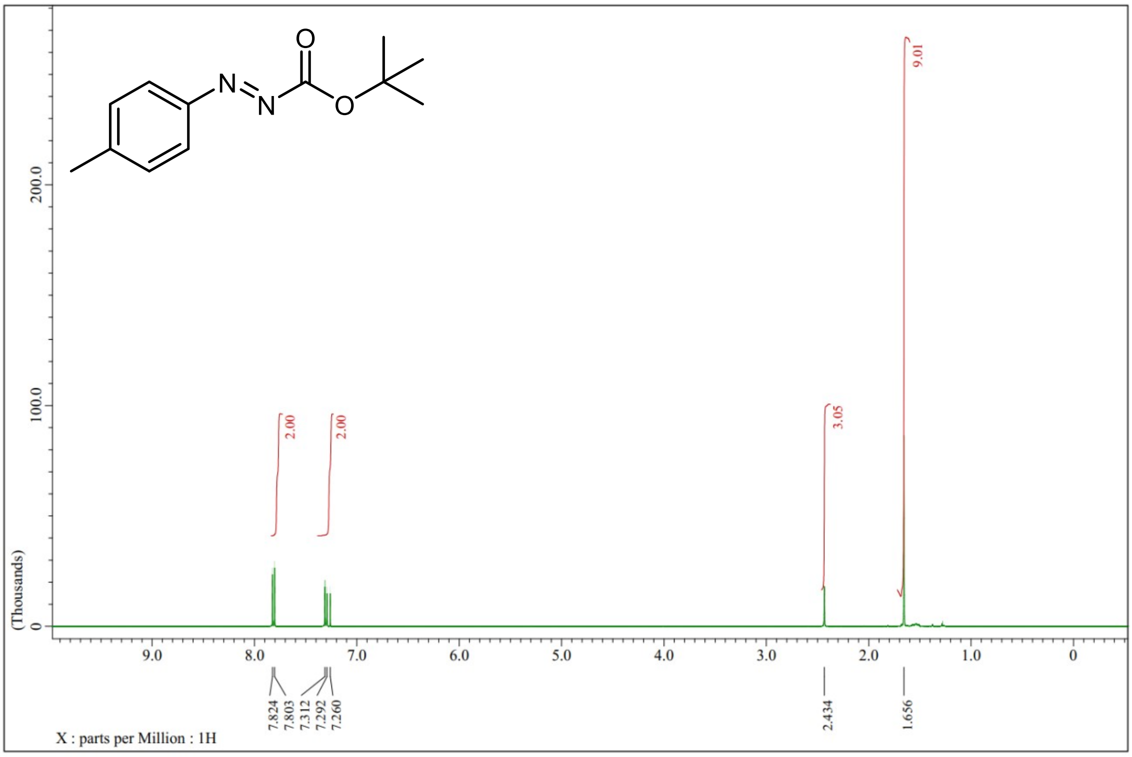

^{1}H NMR spectrum of *tert*-butyl (E)-2-(p-tolyl)diazene-1-carboxylate (**2v**)

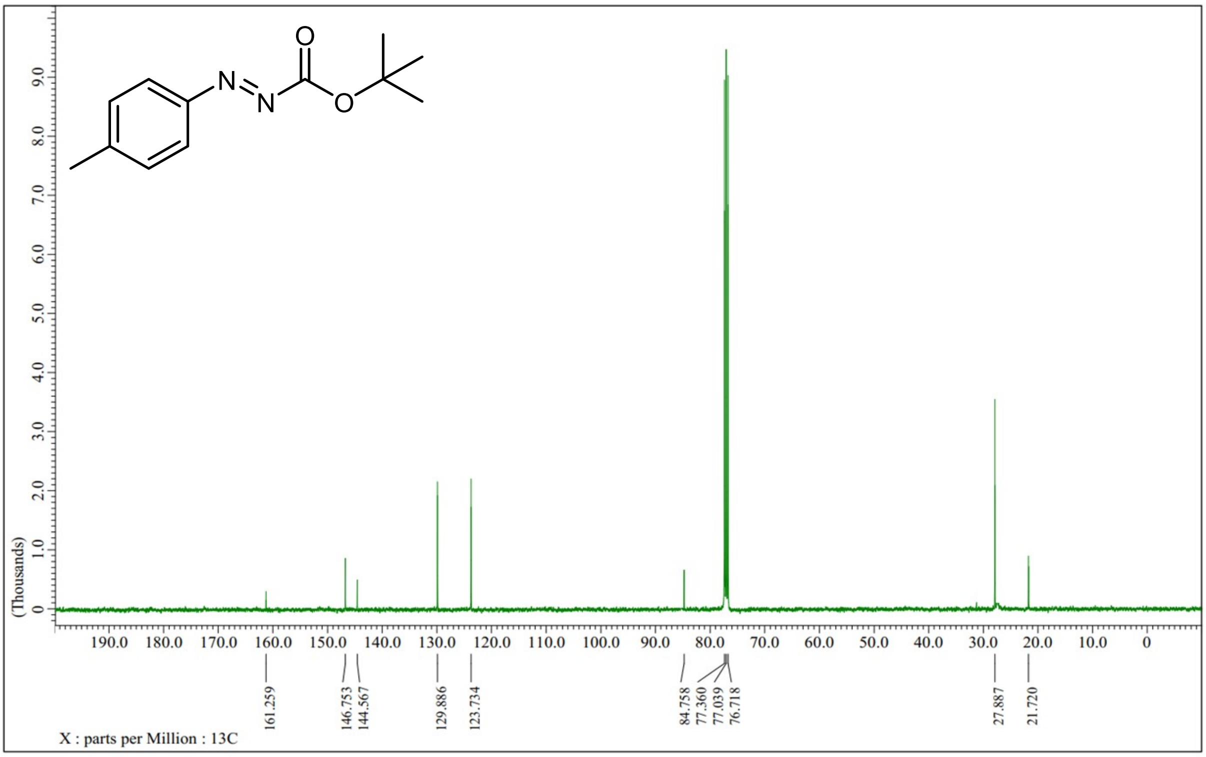

^{13}C NMR spectrum of *tert*-butyl (E)-2-(p-tolyl)diazene-1-carboxylate (**2v**)

***tert*-butyl (*E*)-2-(4-methoxyphenyl)diazene-1-carboxylate (2w)**

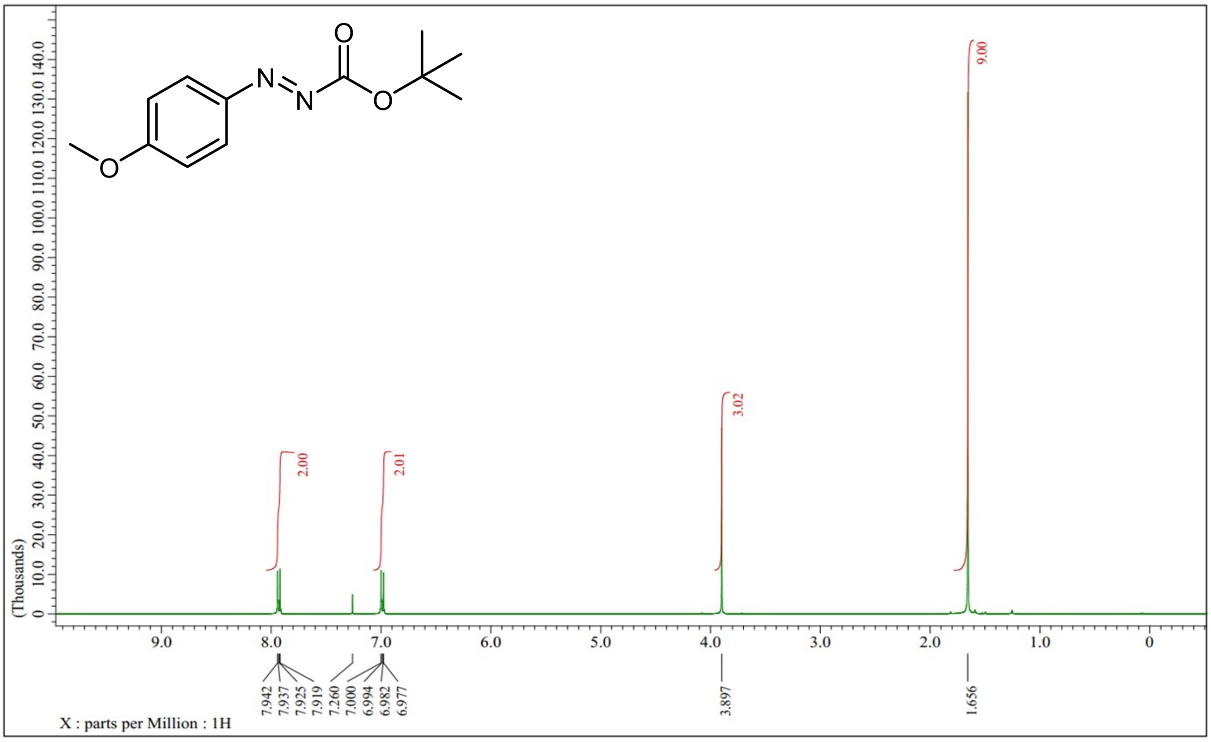

^{1}H NMR spectrum of *tert*-butyl (E)-2-(4-methoxyphenyl)diazene-1-carboxylate (**2w**)

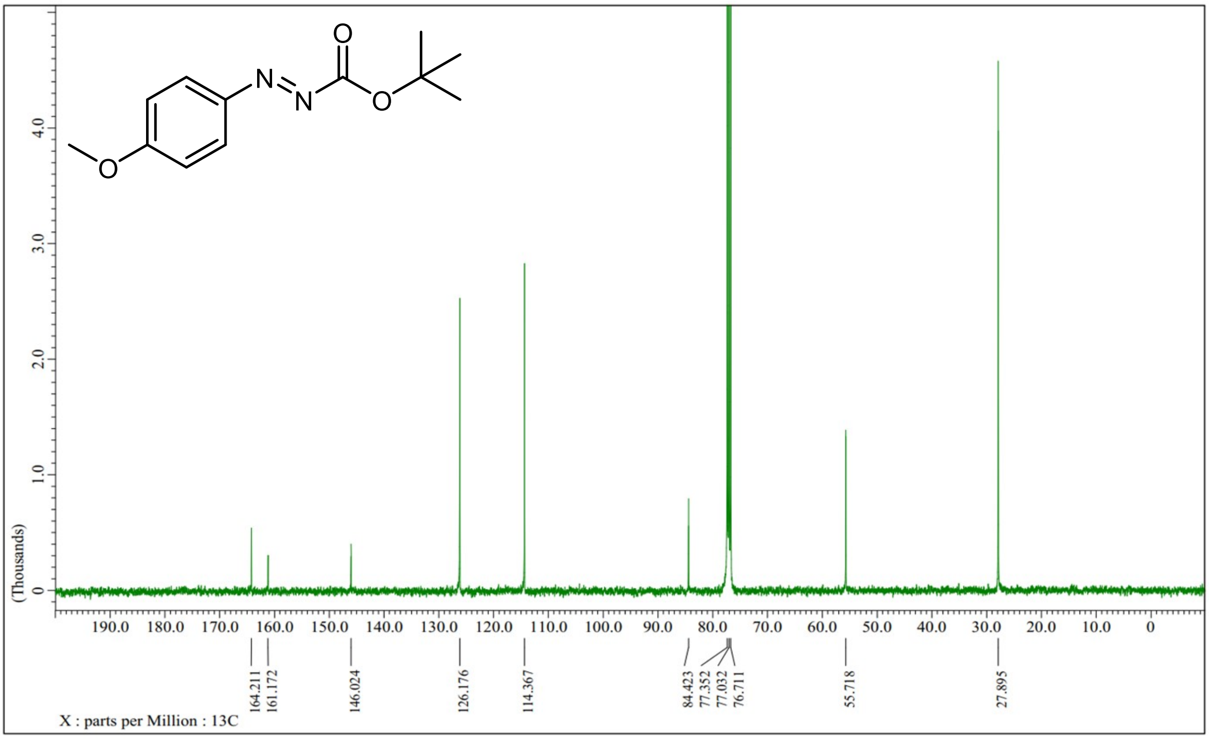

^{13}C NMR spectrum of *tert*-butyl (E)-2-(4-methoxyphenyl)diazene-1-carboxylate (**2w**)

***tert*-butyl (*E*)-2-(4-chlorophenyl)diazene-1-carboxylate (2x)**

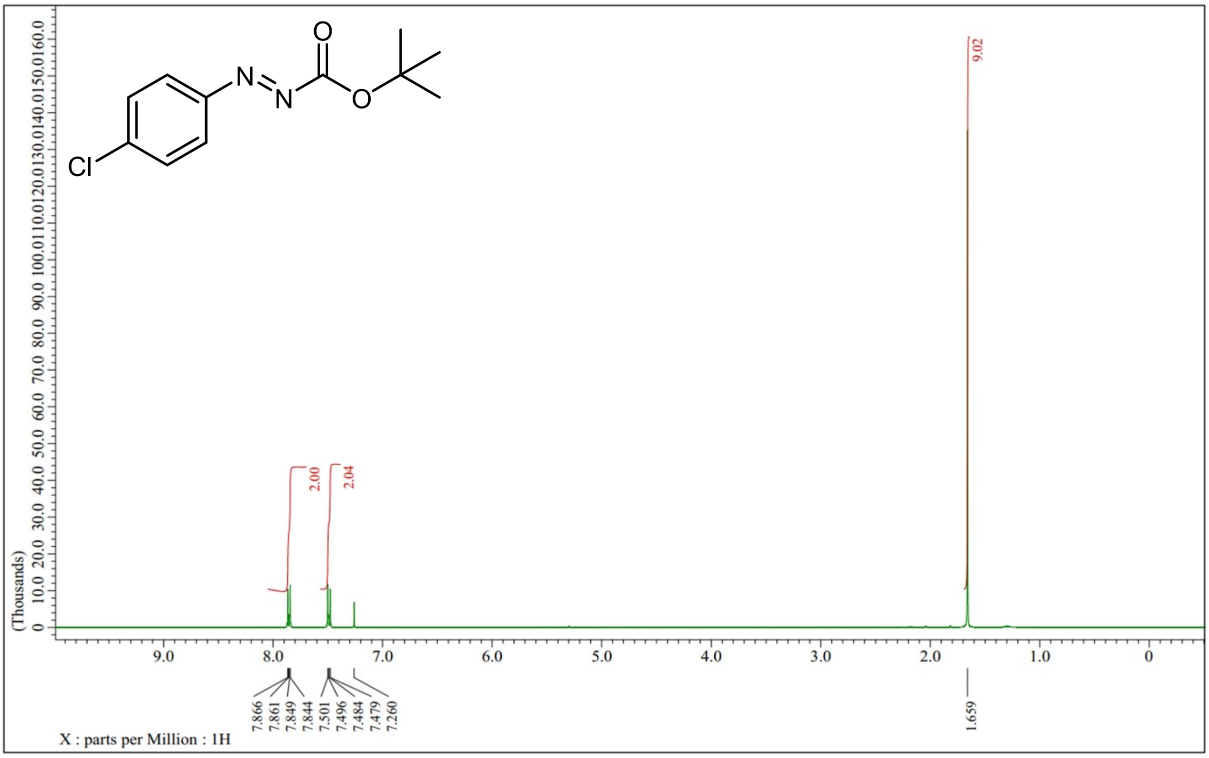

^{1}H NMR spectrum of *tert*-butyl (E)-2-(4-chlorophenyl)diazene-1-carboxylate (**2x**)

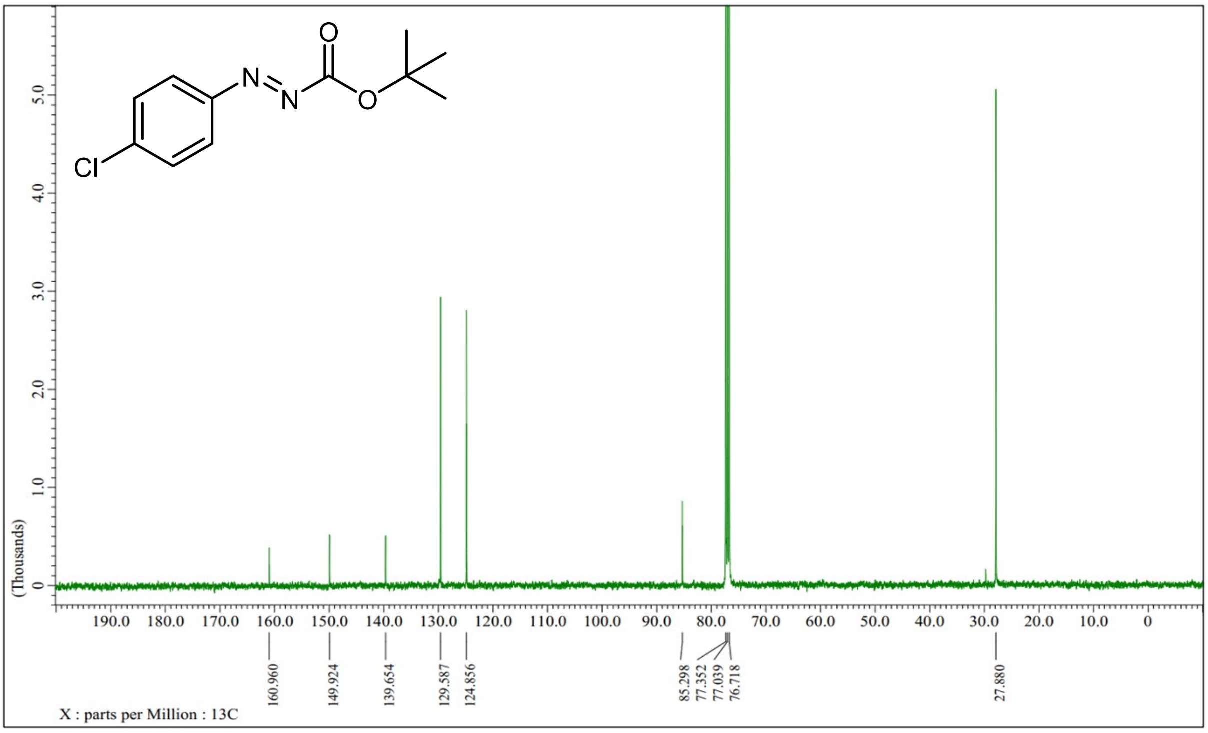

^{13}C NMR spectrum of *tert*-butyl (E)-2-(4-chlorophenyl)diazene-1-carboxylate (**2x**)

Ethyl (E)-2-(pyridin-2-yl)diazene-1-carboxylate (2y)

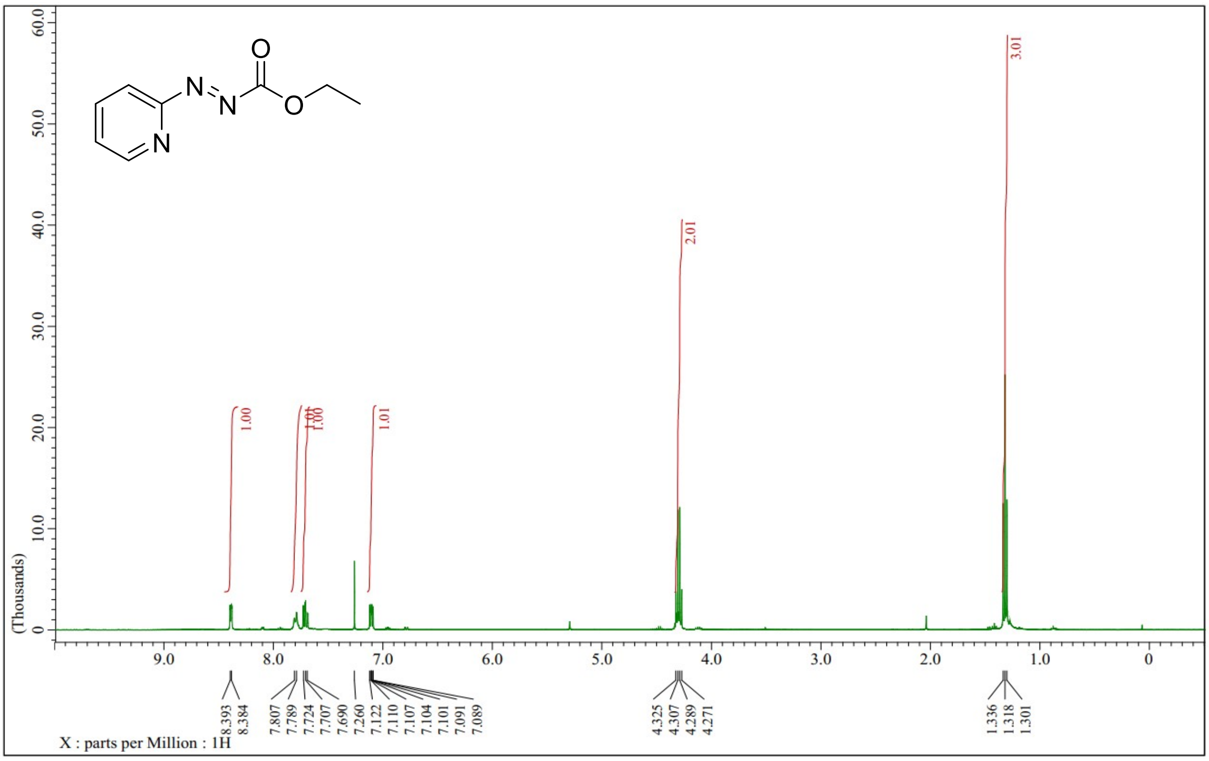

^{1}H NMR spectrum of ethyl (E)-2-(pyridin-2-yl)diazene-1-carboxylate (**2y**)

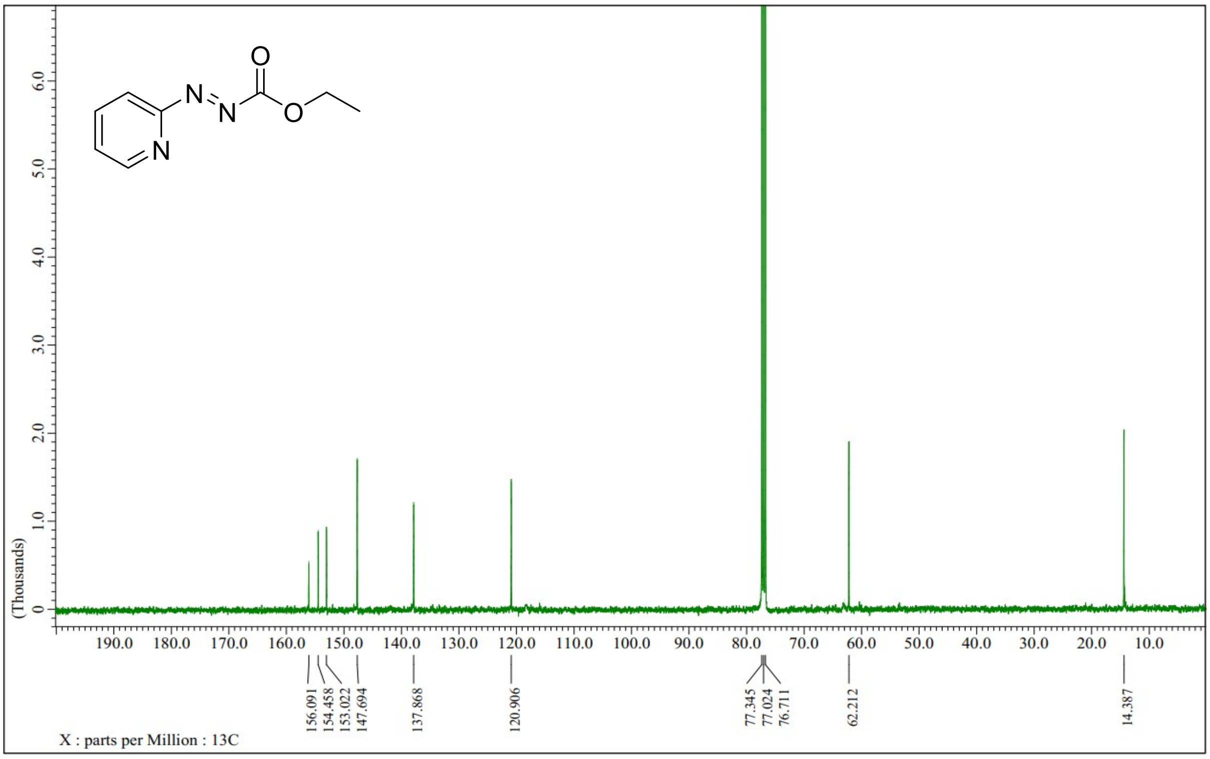

^{13}C NMR spectrum of ethyl (E)-2-(pyridin-2-yl)diazene-1-carboxylate (**2y**)

(E)-1,2-diphenyldiazene (4a)

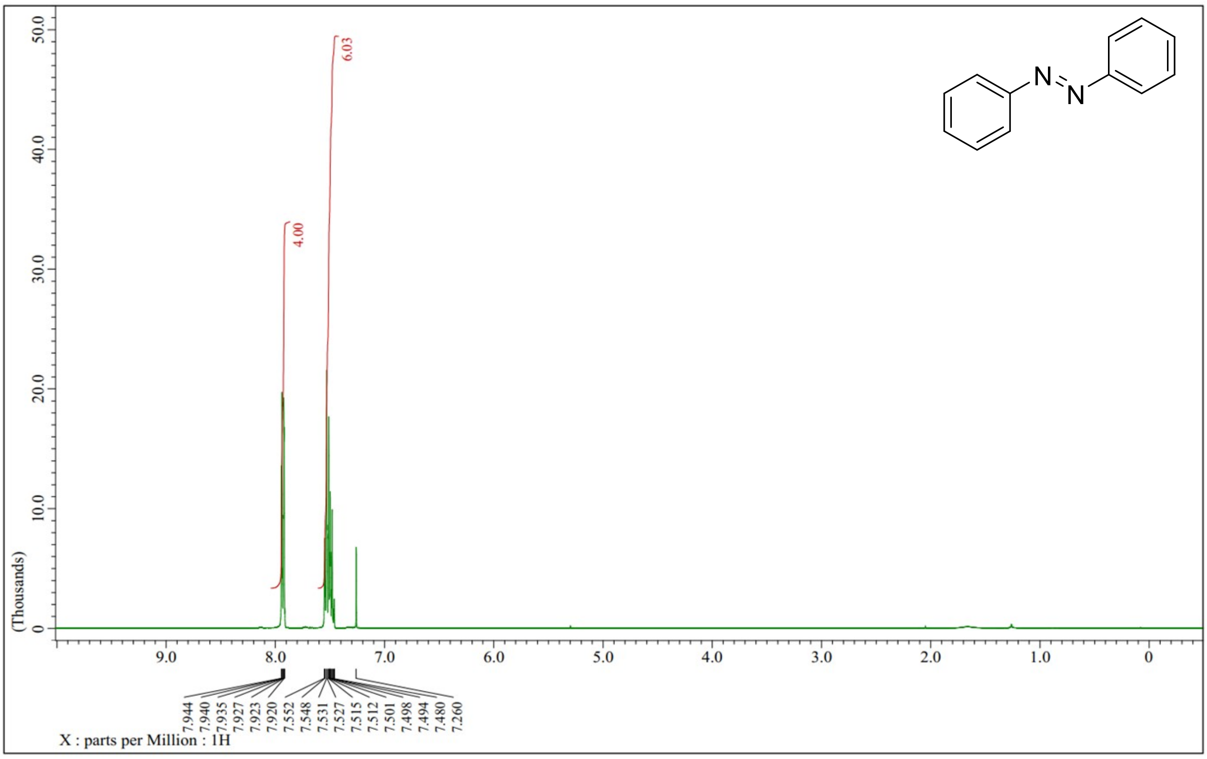

^{1}H NMR spectrum of (E)-1,2-diphenyldiazene (**4a**)

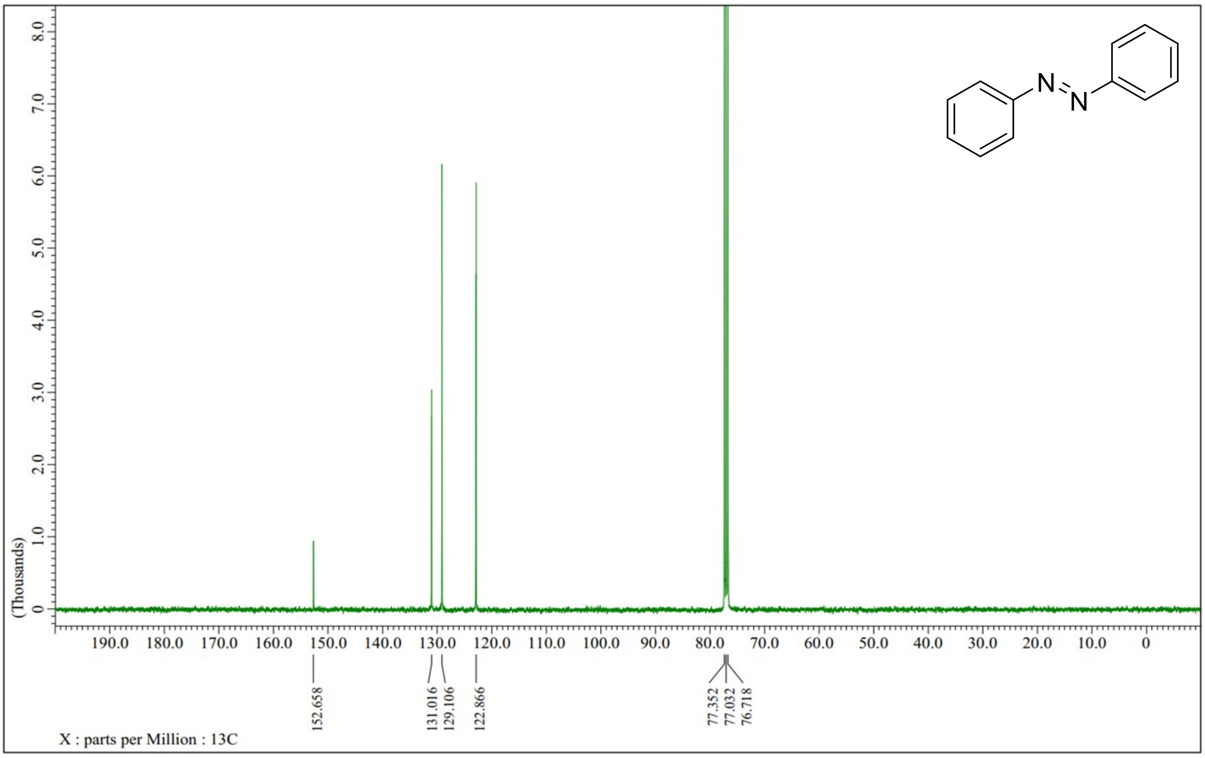

^{13}C NMR spectrum of (E)-1,2-diphenyldiazene (**4a**)

(E)-1,2-bis(4-methoxyphenyl)diazene (4b)

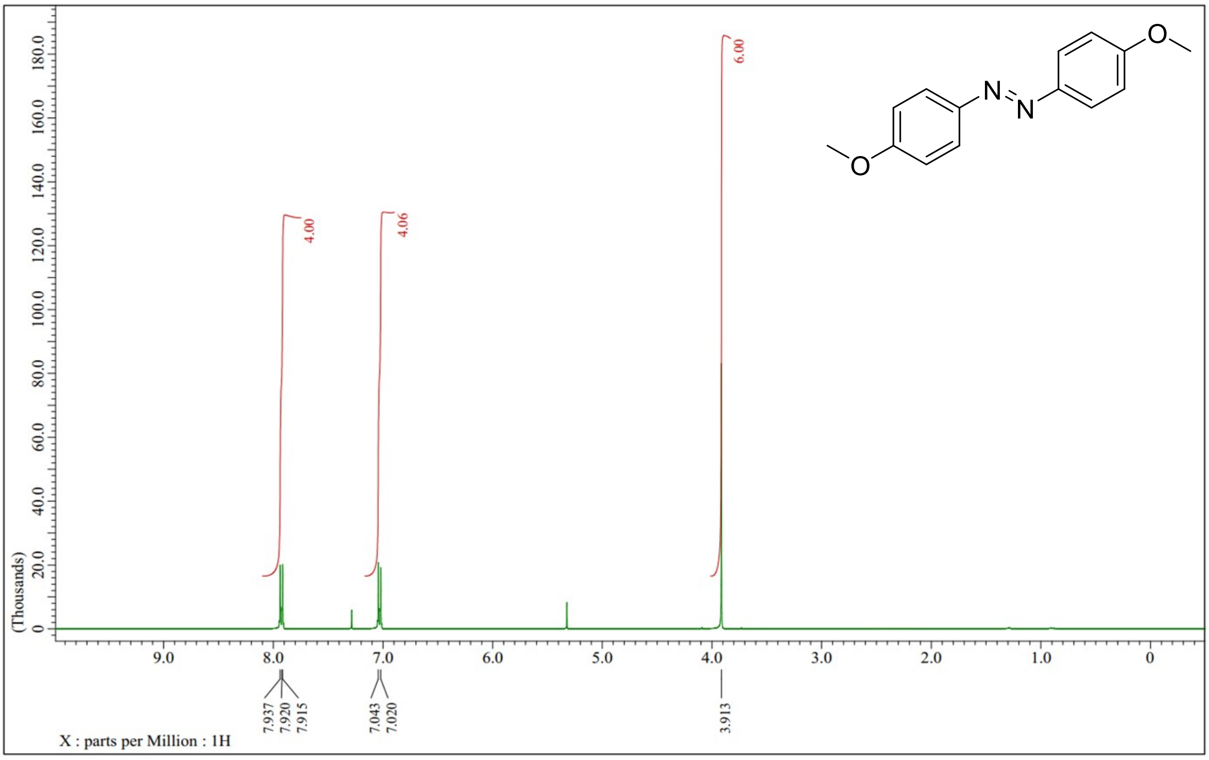

^{1}H NMR spectrum of (E)-1,2-bis(4-methoxyphenyl)diazene (**4b**)

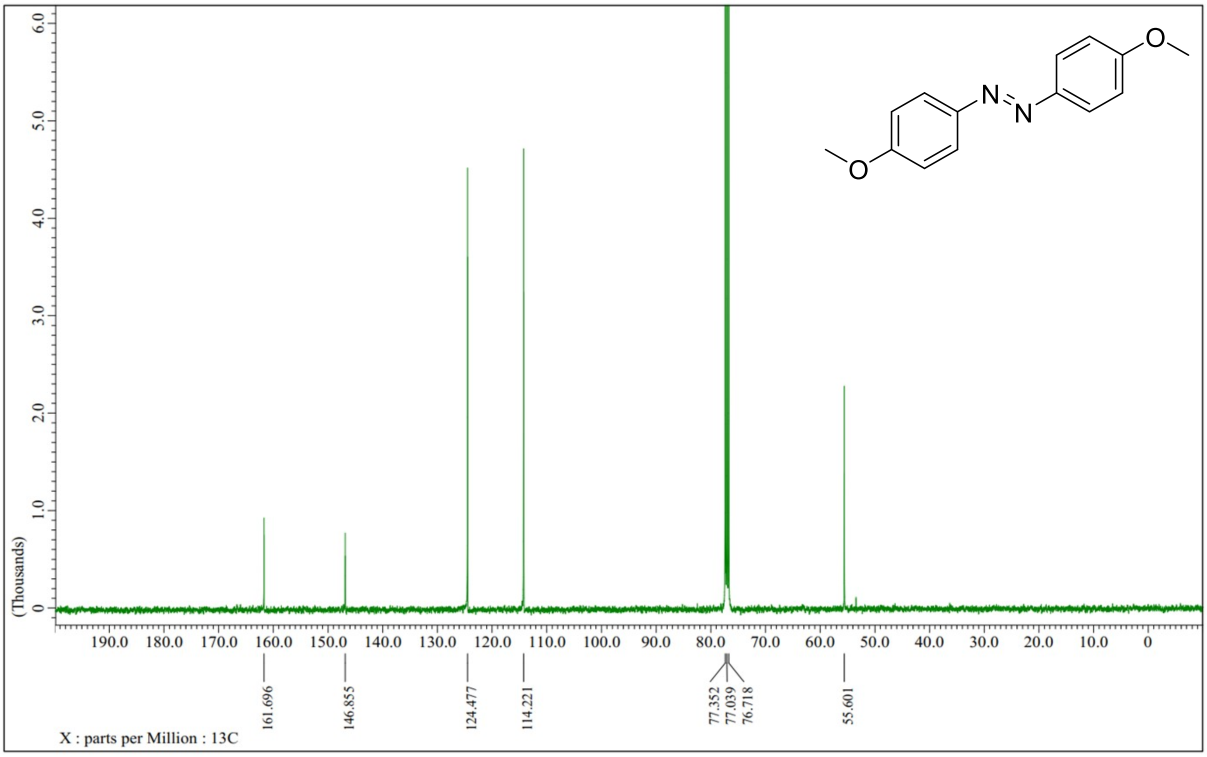

^{13}C NMR spectrum of (E)-1,2-bis(4-methoxyphenyl)diazene (**4b**)

(E)-1,2-bis(4-bromophenyl)diazene (4c)

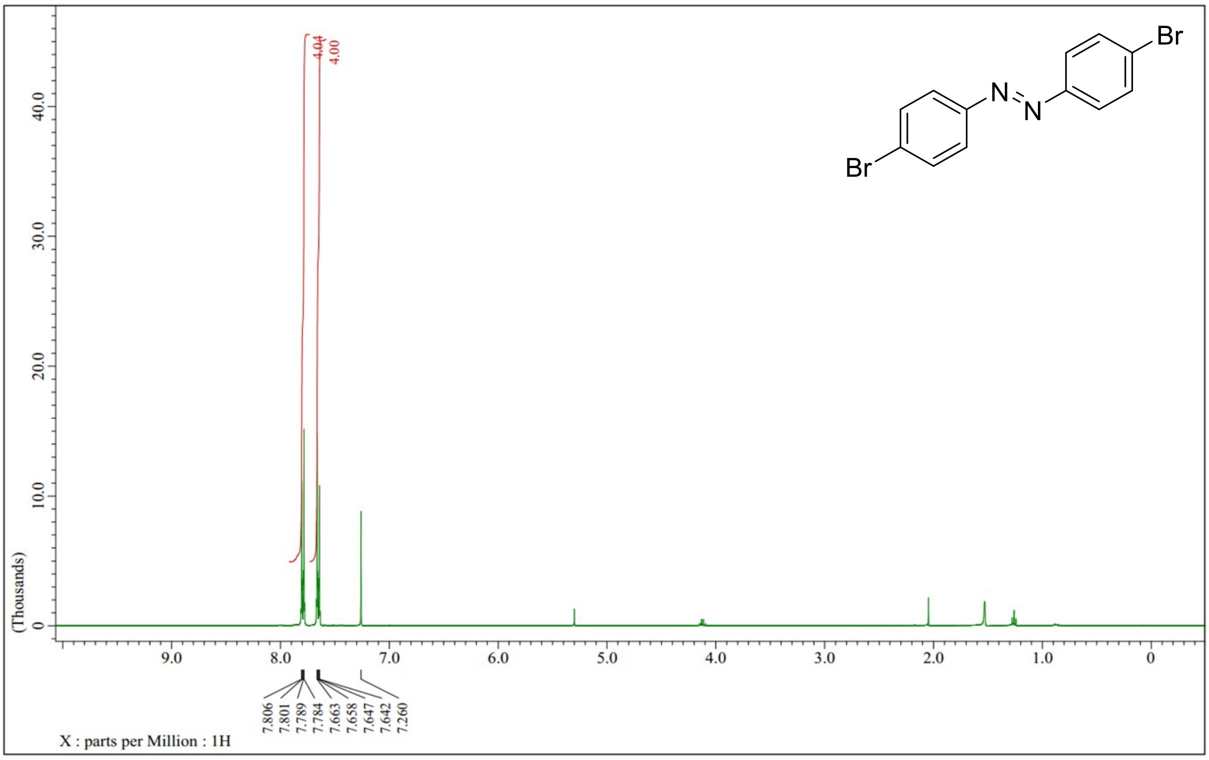

^{1}H NMR spectrum of (E)-1,2-bis(4-bromophenyl)diazene (**4c**)

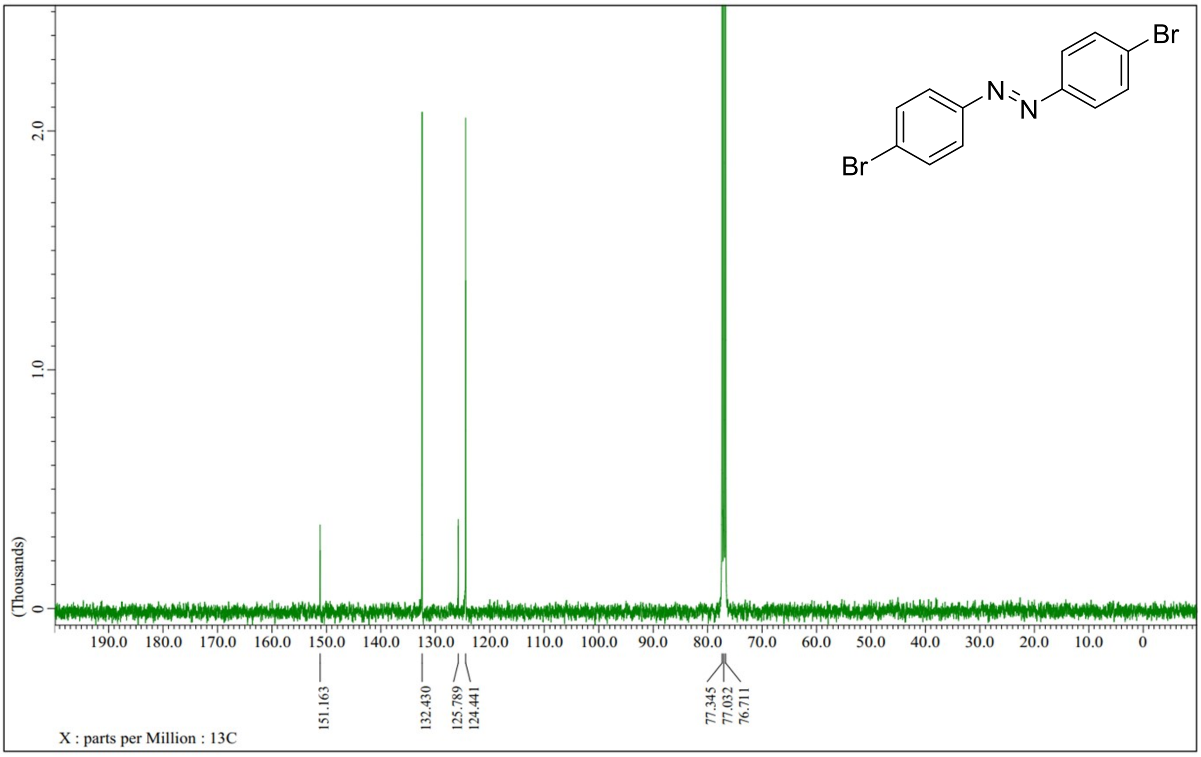

^{13}C NMR spectrum of (E)-1,2-bis(4-bromophenyl)diazene (**4c**)

(E)-1-phenyl-2-(p-tolyl)diazene (4d)

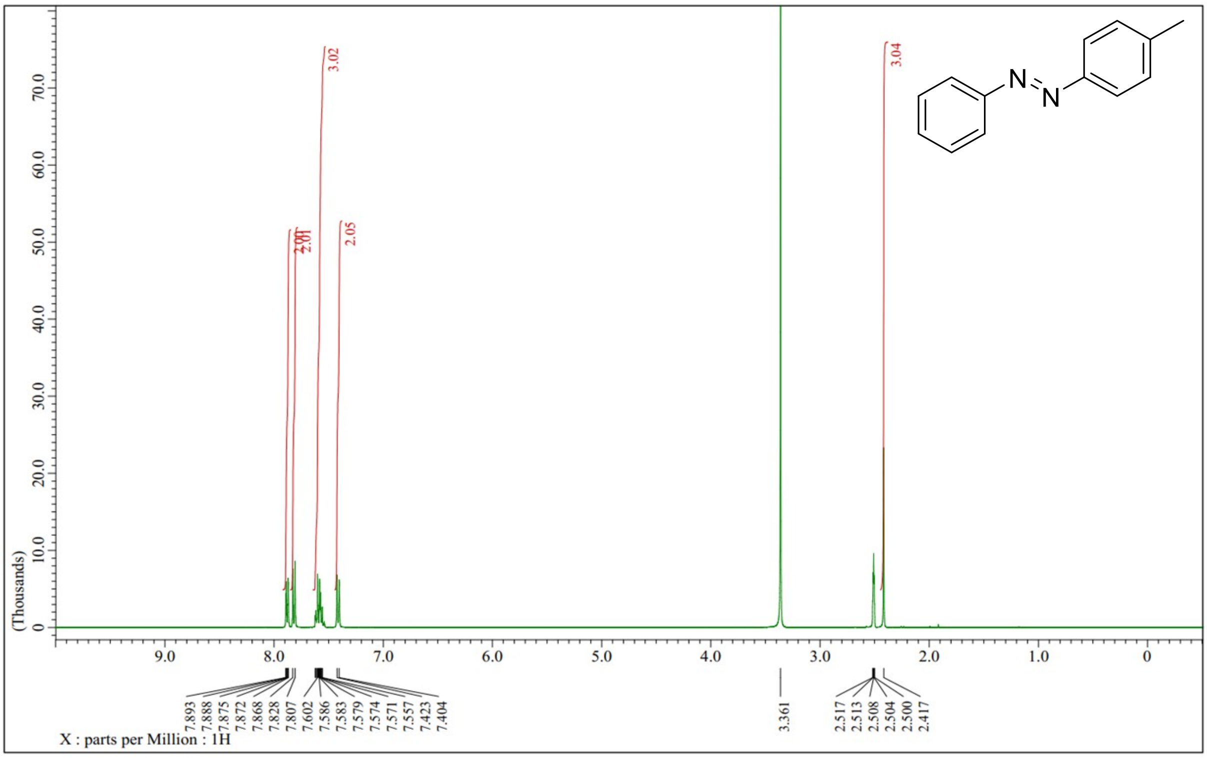

^{1}H NMR spectrum of (E)-1-phenyl-2-(p-tolyl)diazene (**4d**)

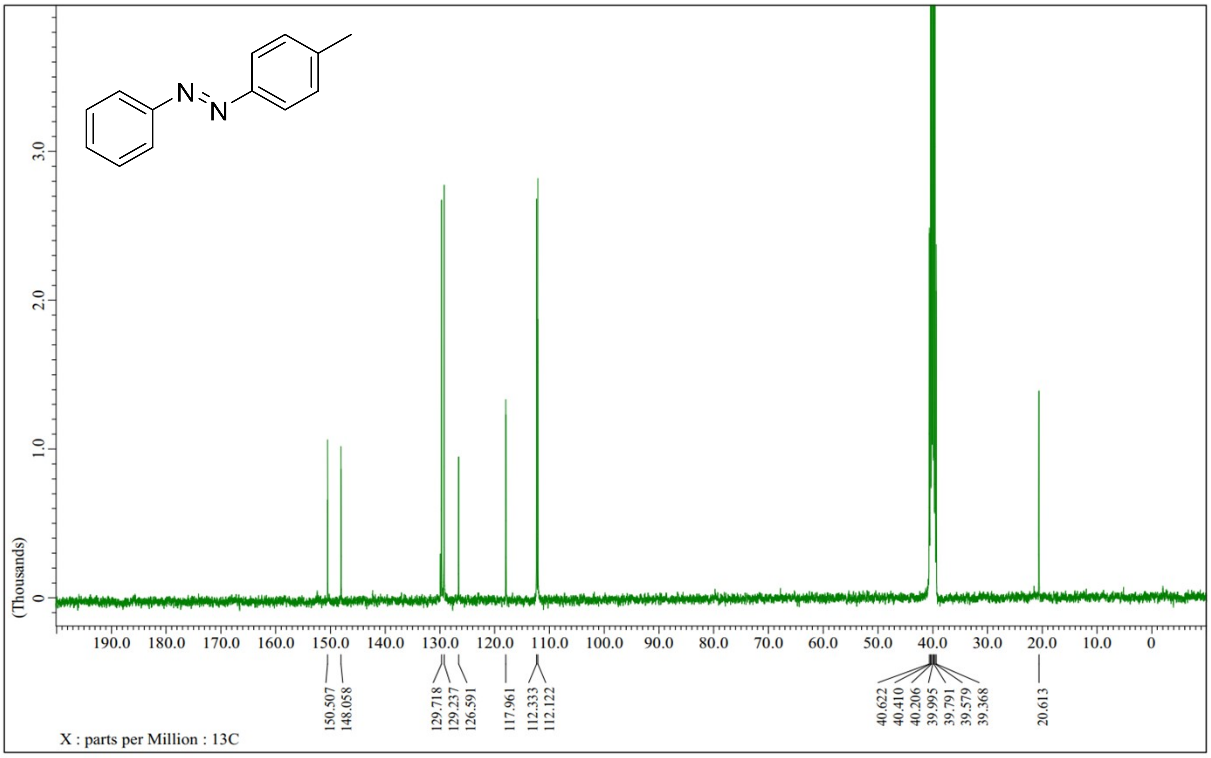

^{13}C NMR spectrum of (E)-1-phenyl-2-(p-tolyl)diazene (**4d**)

(E)-1-(4-chlorophenyl)-2-phenyldiazene (4e)

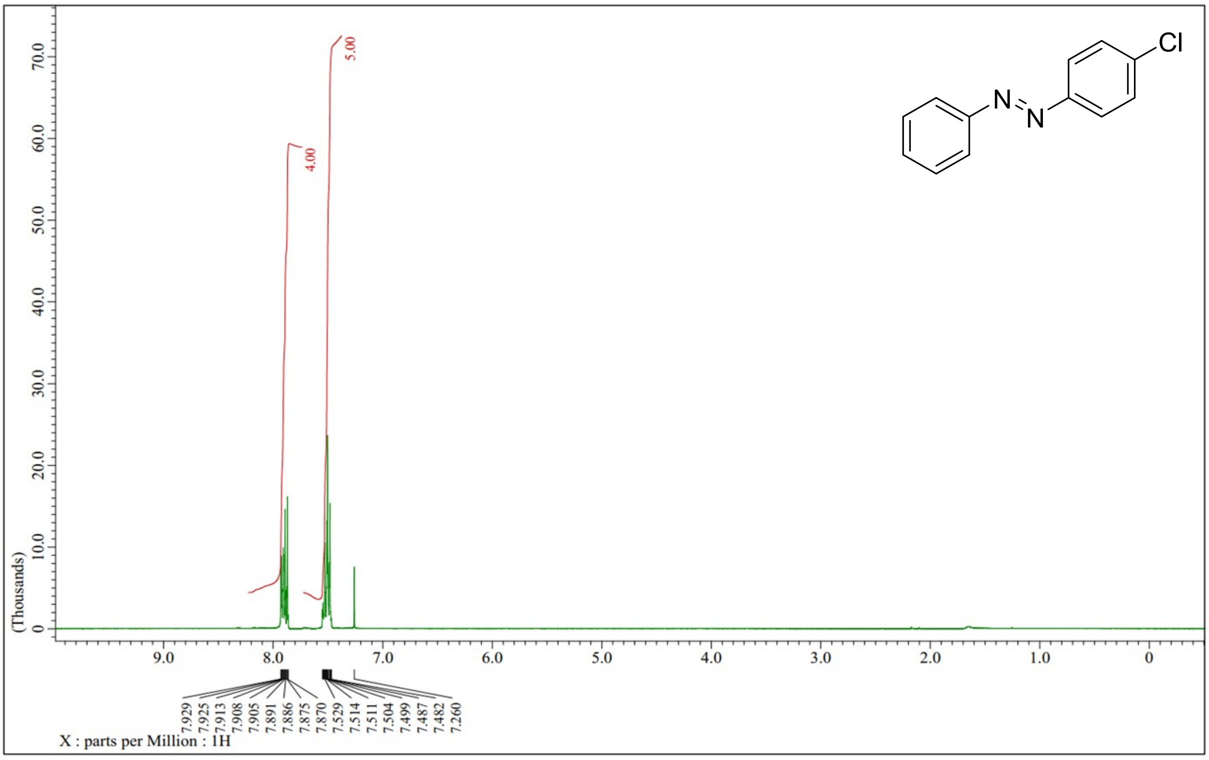

^{1}H NMR spectrum of (E)-1-(4-chlorophenyl)-2-phenyldiazene (**4e**)

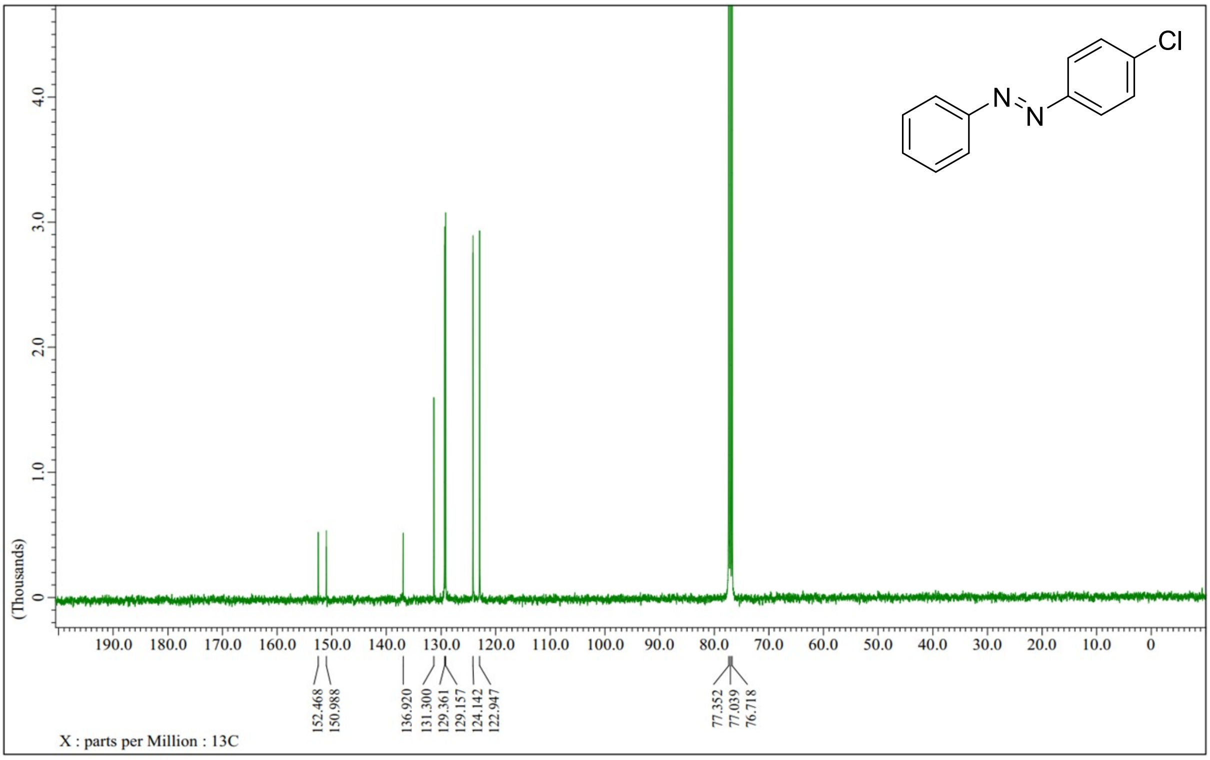

^{13}C NMR spectrum of (E)-1-(4-chlorophenyl)-2-phenyldiazene (**4e**)

Methyl (E)-4-(phenyldiazenyl)benzoate (4f)

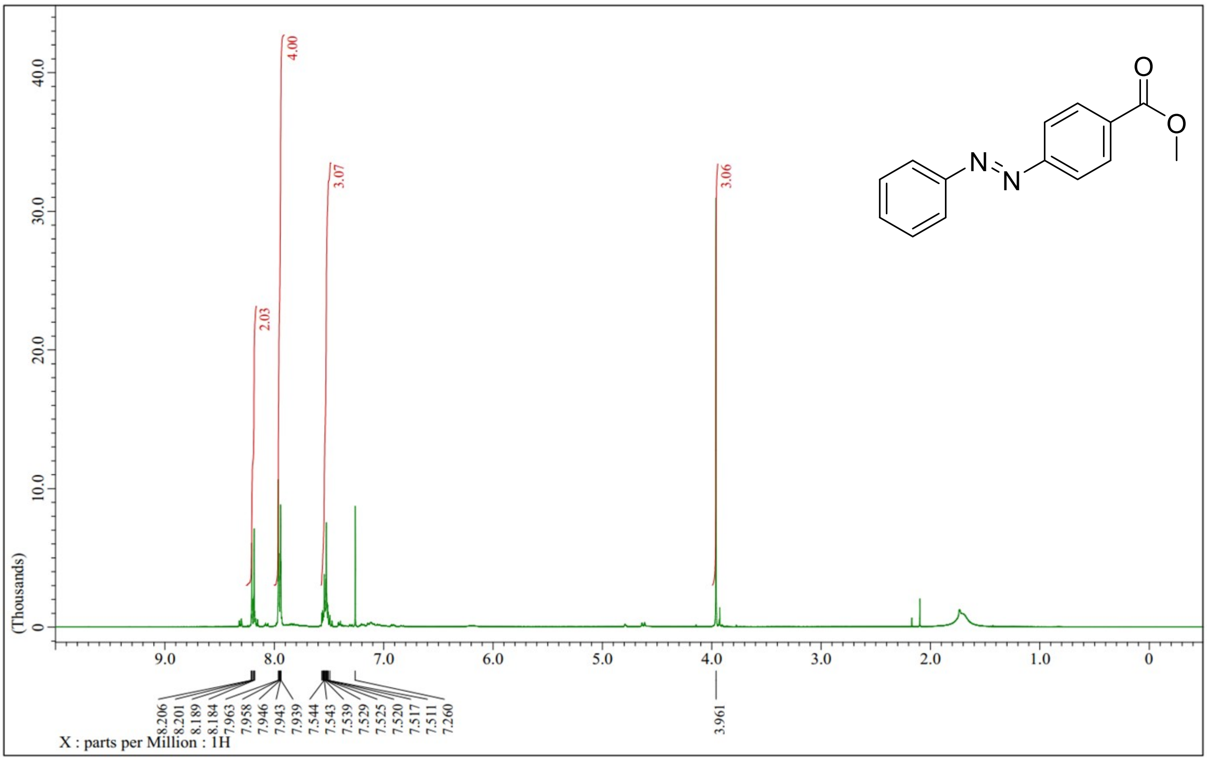

^{1}H NMR spectrum of methyl (E)-4-(phenyldiazenyl)benzoate (**4f**)

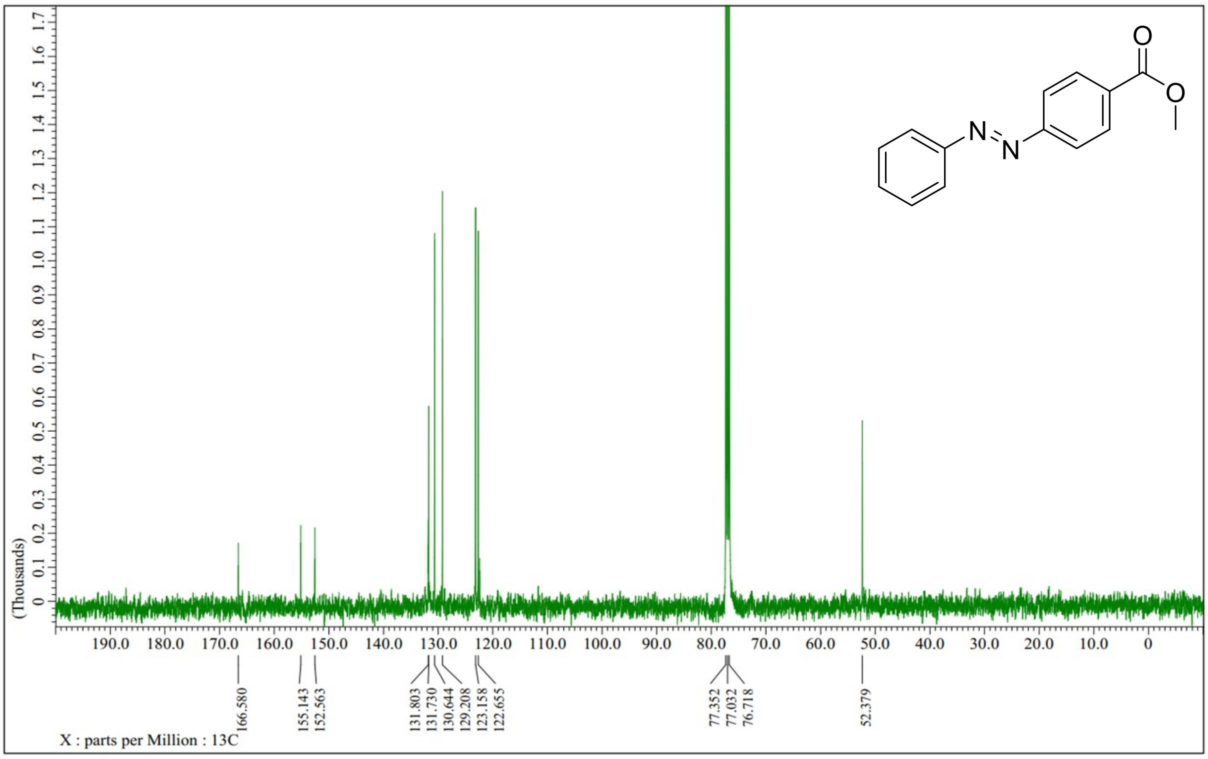

^{13}C NMR spectrum of methyl (E)-4-(phenyldiazenyl)benzoate (**4f**)

(E)-1-benzyl-2-phenyldiazene (6a)

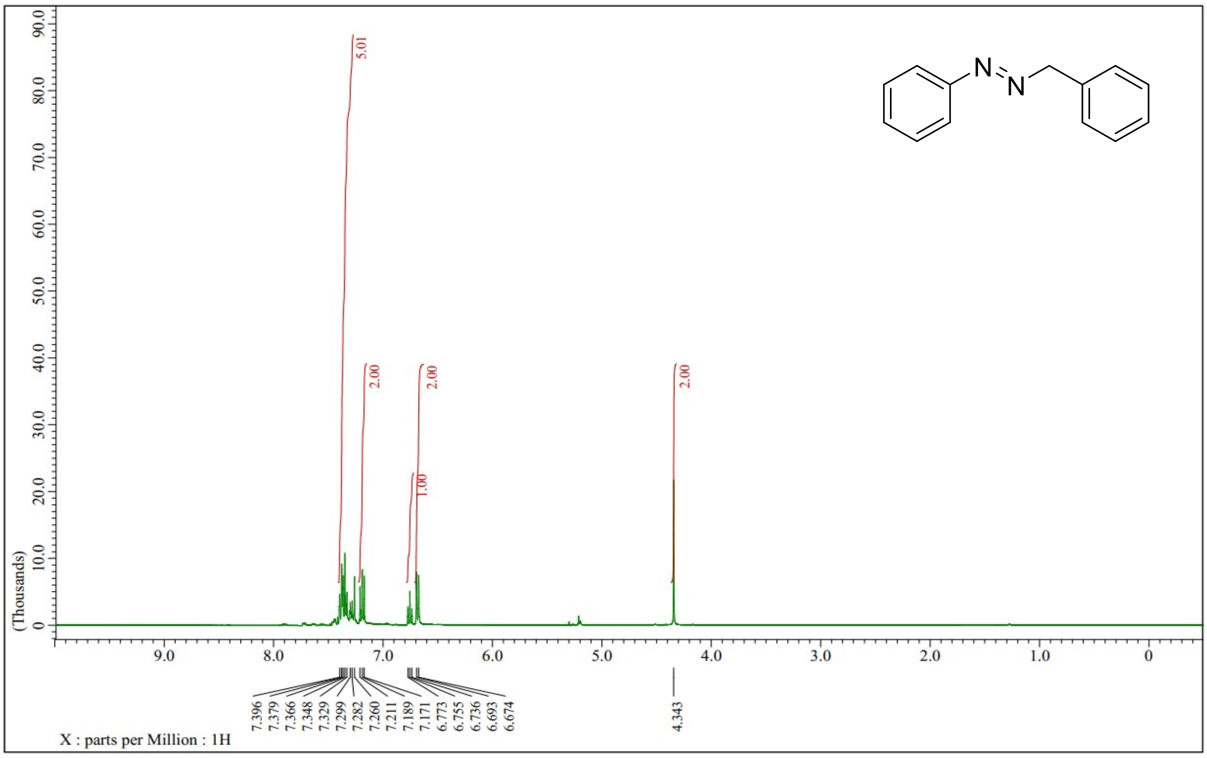

^{1}H NMR spectrum of (E)-1-benzyl-2-phenyldiazene (**6a**)

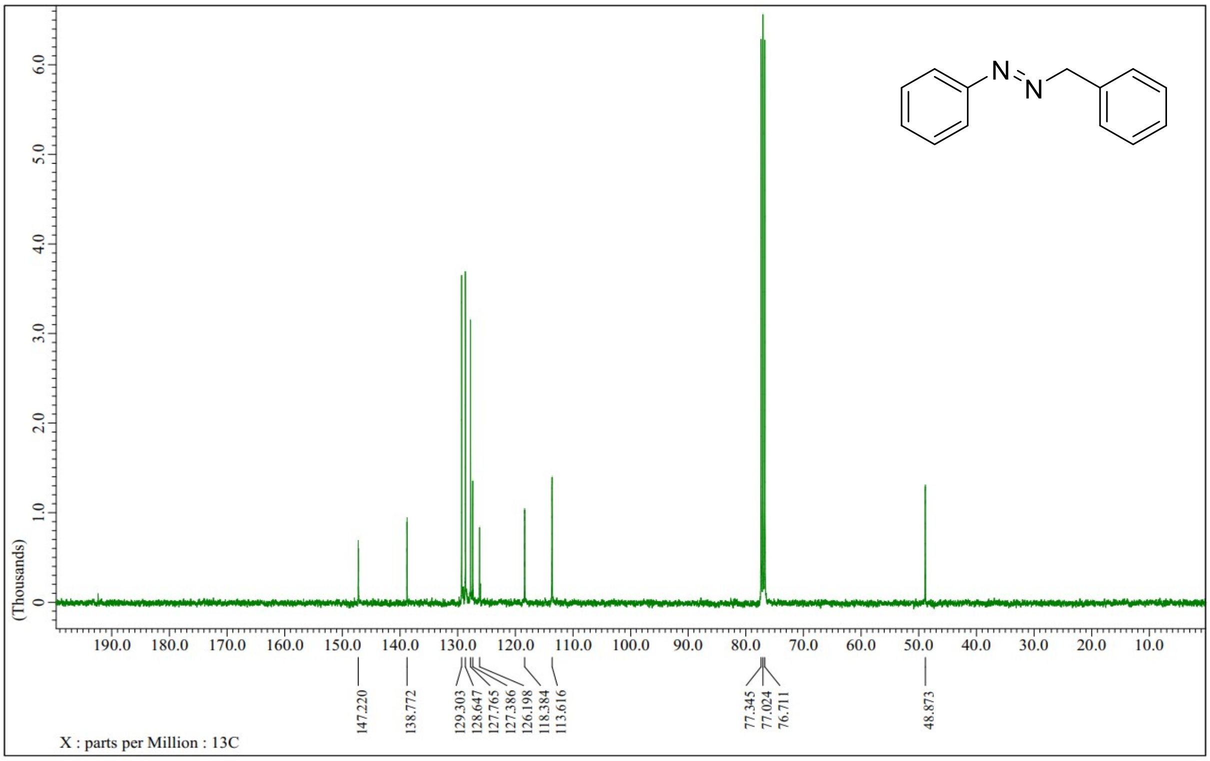

^{13}C NMR spectrum of (E)-1-benzyl-2-phenyldiazene (**6a**)
